# Maternal and perinatal death surveillance and response in low- and middle-income countries: a scoping review of implementation factors

**DOI:** 10.1093/heapol/czab011

**Published:** 2021-03-13

**Authors:** Mary V Kinney, David Roger Walugembe, Phillip Wanduru, Peter Waiswa, Asha George

**Affiliations:** School of Public Health, University of the Western Cape, Bellville, South Africa; School of Health Studies and Faculty of Information and Media Studies, The University of Western Ontario, London, ON, Canada; School of Public Health, Makerere University College of Health Sciences, Kampala, Uganda; Global Health Department of Public Health Sciences, Karolinska Institutet, Stockholm, Sweden; School of Public Health, University of the Western Cape, Bellville, South Africa

**Keywords:** maternal health, maternal and child health, implementation, audit, surveillance, health systems, health systems research

## Abstract

Maternal and perinatal death surveillance and response (MPDSR), or any form of maternal and/or perinatal death review or audit, aims to improve health services and pre-empt future maternal and perinatal deaths. With expansion of MPDSR across low- and middle-income countries (LMIC), we conducted a scoping review to identify and describe implementation factors and their interactions. The review adapted an implementation framework with four domains (intervention, individual, inner and outer settings) and three cross-cutting health systems lenses (service delivery, societal and systems). Literature was sourced from six electronic databases, online searches and key experts. Selection criteria included studies from LMIC published in English from 2004 to July 2018 detailing factors influencing implementation of MPDSR, or any related form of MPDSR. After a systematic screening process, data for identified records were extracted and analysed through content and thematic analysis. Of 1027 studies screened, the review focuses on 58 studies from 24 countries, primarily in Africa, that are mainly qualitative or mixed methods. The literature mostly examines implementation factors related to MPDSR as an intervention, and to its inner and outer setting, with less attention to the individuals involved. From a health systems perspective, almost half the literature focuses on the tangible inputs addressed by the service delivery lens, though these are often measured inadequately or through incomparable ways. Though less studied, the societal and health system factors show that people and their relationships, motivations, implementation climate and ability to communicate influence implementation processes; yet their subjective experiences and relationships are inadequately explored. MPDSR implementation contributes to accountability and benefits from a culture of learning, continuous improvement and accountability, but few have studied the complex interplay and change dynamics involved. Better understanding MPDSR will require more research using health policy and systems approaches, including the use of implementation frameworks.

**KEY MESSAGES**

- Using an implementation framework allows for deeper understanding of factors influencing implementation of maternal and perinatal death surveillance and response (MPDSR), which is a complex intervention process aimed at preventing maternal and perinatal deaths.
- The literature on MPDSR implementation primarily focuses on tangible inputs from a service delivery lens, though few of these inputs were adequately documented or measured.
- Studies show that people, their relationships and communication channels are at the heart of the implementation process; yet their subjective experiences and relationships are inadequately focused on in the current literature.
- Understanding the complex interplay and change dynamics of MPDSR implementation requires health policy and systems approaches, which includes but is broader than the current programmatic focus of MPDSR evidence.

## Introduction

Maternal and perinatal death surveillance and response (MPDSR), or any form of maternal and/or perinatal death review or audit, is a process used to improve health services and pre-empt future avoidable deaths (Hounton et al. 2013; Independent Expert Review Group 2014; Every Woman Every Child 2015). As an intervention, it is a continuous cycle of identification, notification and review of maternal and/or perinatal deaths followed by actions to address identified contributing factors and to prevent future deaths through acting on gaps identified in the audit (Kinney et al. 2019). With an aim to influence health professional behavior, health system functioning and patient health as well as improve maternal and perinatal health outcomes, MPDSR functions at multiple levels of the health system to capture information on the number and causes of deaths and to undertake systematic, critical analysis of the care received (Ivers et al. 2012; Kerber et al. 2015; WHO 2016a).

In the past 15 years, there has been growing momentum to strengthen and expand the intervention (WHO 2004; WHO 2016c; E4A 2017; WHO 2017), culminating in World Health Organization (WHO) global technical guidelines (WHO 2013a; WHO 2016a; Supplementary File 1). As a result, many low- and middle-income countries (LMIC) have adopted national guidelines, however, few have robust MPDSR systems (Kerber et al. 2015). A growing number of studies have investigated the implementation of MPDSR in selected countries, and some reviews have explored implementation factors separately for maternal death reviews or perinatal death audits (Pattinson et al. 2009; Kerber et al. 2015; Martin et al. 2016; Lusambili et al. 2019). While valuable contributions to the literature, these previous reviews do not consider implementation theory to assess factors influencing MPDSR implementation nor do they consider the full range of types of maternal and/or perinatal death reviews (Kerber et al. 2015; Martin et al. 2016; Smith et al. 2017c). Investigation of quality improvement processes, including audit and feedback (Ivers et al. 2012), benefits from the use of implementation theory and frameworks to understand and explain factors that influence implementation outcomes (Hulscher et al. 2013; Davidoff et al. 2015; Nilsen 2015; Akachi and Kruk 2017; Kruk et al. 2017; Persson 2017; Topp 2017).

**Figure 1:**
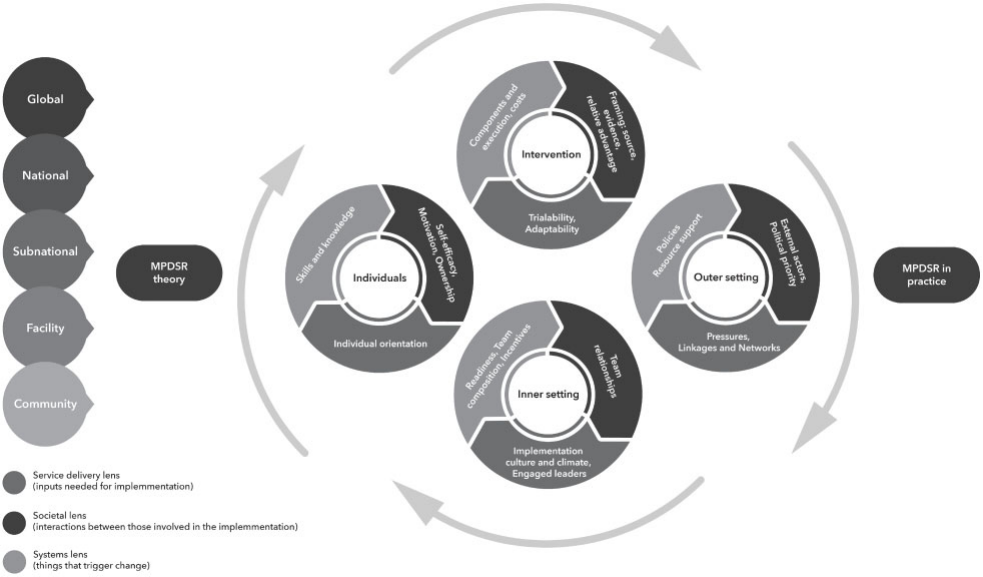
Theoretical framework for studying MPDSR implementation—around here.

The aim of this scoping review is to map and synthesize factors that support or hinder implementation of MPDSR, or related forms, using a theory-based conceptual implementation framework. It also explores common, if any, implementation factors between the types of maternal and/or perinatal death reviews. For MPDSR to function, as intended, the process needs to link across health system levels, adapt to context, enable a learning climate that supports individuals to critically think and collaborate, so that agents can initiate and sustain change. In order to understand the implementation factors identified in the current literature, we developed a theory-based conceptual framework (Kinney et al. 2019), described in Box 1 and visualized in Figure 1, to unpack the different levels and different factors that influence implementation of this complexity intervention process. The framework includes 24 constructs within the four domains (intervention, individual, inner and outer settings) as well as three cross-cutting lenses within each domain that are used to understand and measure health system drivers of women’s and children’s health (George et al. 2019).

## Methods

### Protocol

The protocol for this scoping review presents the methods (Kinney et al. 2019), which were guided by an adapted Arksey and O’Malley approach (Arksey and O'Malley 2005; Levac et al. 2010; Peters et al. 2017) and applied the Preferred Reporting Items for Systematic reviews and Meta-Analyses extension for Scoping Reviews (PRISMA-ScR) Checklist by Tricco et al. (2018; Supplementary 3, Table S3.1). The scoping review followed six stages: (1) identify the research question; (2) identify relevant studies; (3) study selection; (4) data collection; (5) data summary and synthesis of results; and (6) consultation.

The research questions included: first ‘What do we know about implementation of maternal death audit, perinatal death audit or combined audit approaches and the factors that either support or hinder the implementation process?’; and second, ‘How can an implementation framework help to explain the implementation factors and their interactions?’

### Eligibility, information sources and search

We included all literature that reports on the implementation of maternal and/or perinatal death surveillance and responses or maternal and/or perinatal death audit published in English between 2004 and July 2018 from LMICs. The start year was selected to coincide with the first WHO maternal death review guideline (WHO 2004). The literature included peer-reviewed publications as well as published and unpublished (grey) literature, such as reports. We also considered reviews and commentaries in the screening process.

We piloted and determined the search terms using PubMed (Supplementary 3, Table S3.2). In August 2018, literature was drawn from academic databases and online search engines (PubMed, CINAHL, SCOPUS, Web of Science, JSTOR, LILACS, the WHO Library, Maternal Death Surveillance and Response Action Network, and Google) using specific search terms (Table 1). From August 2018 to January 2019, we also identified literature through expert consultation, including members of the WHO’s MPDSR Technical Working Group. Finally, we searched the reference lists of all identified records for any additional records, not previously identified.

**Table 1:** Overview of search strategy components

Summary of search terms	(‘maternal mortality’ OR ‘perinatal death’ OR ‘maternal death’ OR ‘perinatal mortality’ OR ‘fetal mortality’ OR ‘stillbirth’) AND (‘audit’ OR ‘surveillance and response’).
Concept component	All forms of maternal and perinatal death review including obstetric audit, MPDSR, maternal death surveillance and response (MDSR), maternal death review (MDR) Limited to studies or perspectives that identify factors that influence the implementation process Excluded near miss audits as well as other forms of maternal and perinatal death surveillance, e.g. confidential inquiries, social autopsy and verbal autopsy.^a^
Context component	Limited to LMICs listed by the World Bank in 2018.

a For definitions of these terms, please see Lewis (2014a).

### Selection of sources of evidence (screening)

Reviewers (M.V.K., D.R.W., P.Wanduru) initially screened 20 titles together to ensure consistency in approach. Then two reviewers independently screened the titles, abstracts and full text. In the cases where abstracts were not available, the full text was screened. A third party resolved all discrepancies between reviewers independently; the third party for the full text screening was conducted by A.S.G and P.Waiswa. The reviewers met on a weekly basis during the screening process to discuss any issues arising from the process and revolved disagreements by consensus.

### Data charting process

A data-charting tool was conceptualized by the research team collectively, developed in Microsoft Excel and piloted during a workshop in August 2018 (Supplementary 3, Table S3.3). The three reviewers (M.V.K, D.R.W., P.Wanduru) independently extracted data from three studies, and the results were discussed with the full team. This piloting process led to revisions to the tool as well as cohesion in the team around the data extraction process. The three reviewers then independently charted the data; discussed issues in weekly meetings; and continuously updated the data-charting form in an iterative process. A record of changes was documented in the Excel file.

### Data items

Data extracted included key reference characteristics, e.g. type of record (i.e. document type, methods, level of study), background to the record (i.e. country, type of organization) and content of record (i.e. focus of intervention, history, scale of study—cross-country national, subnational, facility; the full tool is available in Supplementary 3, Table S3.3). The components of the framework were organized by domain and entered in as ‘not described’ or ‘described’. A short explanation on how it was described was then entered in, when applicable.

### Synthesis of results

We grouped the records by studies (including academic journal articles and reports), academic reviews and academic commentaries. We then analyzed the reference characteristics and framework components by group. Data analysis of the framework components involved qualitative thematic and content analysis (Vaismoradi et al. 2013).

### Consultation

We engaged key stakeholders throughout the process, including the WHO’s MPDSR Technical Working Group and the Countdown to 2030 Drivers Technical Working Group, to identify any additional literature, to input on the implementation framework and to review the findings to support interpretation (Supplementary 3, Table S3.4). Additional meetings were set up with targeted experts to receive further inputs.

## Results

### Selection of records

**Figure 2:**
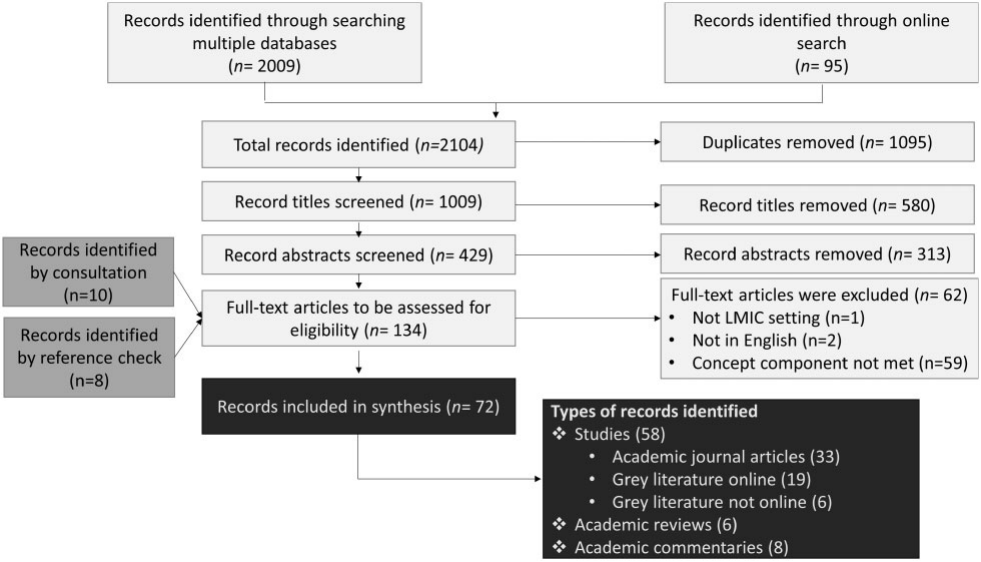
Flow diagram—around here.

Figure 2 shows the flow diagram documenting the screening process; Table 2 provides the results of the search process by source. The systematic database and online search yielded 2104 records. After removing duplicates, 1009 records were screened by title followed by 429 records screened by abstract. Consultation and checking the references of identified papers resulted in 18 additional records screened (totaling 1027 records screened between the online systematic process and the consultation process). A total of 134 records underwent full text review. Of the 72 records meeting inclusion criteria, 58 were studies (either academic journal articles or reports), 6 were academic reviews and eight were academic commentaries.

**Table 2:** Results of search by source

Database	Number of articles
PubMed	434
CINAHL	264
SCOPUS	658
Web of Science	432
JSTOR	214
LILACS	7
Database search	2009
MDSR Network	16
WHO IRIS	50
Google	29
Online search	95
Consultation	8
Reference list	10
Additional search	18
Total identified	2122
Duplicates	1095
Total screened	1027

### Characteristics of sources of evidence

Supplementary 4, Table S4.1 provides an overview of the record characteristics. Among the 58 studies, 24 LMICs are represented including six from Tanzania, four from Malawi and three each from Ethiopia, Nigeria, South Africa, Uganda and Zimbabwe. Two-thirds of the studies are from the sub-Saharan African region (66%); 12% from South Asia region, 3% from East Asia and the Pacific region, 3% from the Middle East and North Africa region, and 12% described as international. The level of study varies greatly with 33% being a combination of geographic levels, 26% at national level, 24% from selected facilities, 9% at subnational level and 7% at global or multicountry level. We found 10 studies published from 2004 to 2010 and 48 studies from 2011 to July 2018.

Half of the studies (53%) focus on maternal death audit processes with 20 and 11 studies concentrating on maternal death review and MDSR, respectively. Combined maternal and perinatal death reviews have 12 studies (21%), and there are 7 studies specific to MPDSR (12%). Five studies focus on perinatal death audit (9%) and another four studies on other related forms of audit (e.g. obstetric audit). The studies mostly consider a combination of macro, meso and micro levels (64%); although 11 studies focus specifically at the macro level, eight studies at the meso level and two studies at the micro level. The majority of the studies were qualitative (45%) or mixed methods (28%) with only 5% using quantitative approaches and 22% of studies not indicating research methods.

Nearly half of the studies do not specify funding support (41%); of those that do, 24% report bilateral support, 12% report funding from nongovernmental or academic organizations and 10% report funding from foundations. Half of the author teams include a mixture of organizations including national governments (52%); academics comprise a quarter of the studies (26%) and the reminder of the studies include authors from government (2%), nongovernment (7%), a mix of organizations not including government (7%), or independent or other (6%). Over half of the first author affiliation comes from LMIC (69%), although the top two countries of author affiliation are the UK (21%) and USA (9%).

### Understanding MPDSR

Using the constructs from the implementation framework, 601 data points from the 58 primary studies were extracted (e.g. construct was described) and analysed. The outer setting, intervention and inner setting domains have the greatest number of data points (27, 29 and 30% respectively). In contrast, the individual domain has the fewest data points (13%; Supplementary 4, Table S4.2). Nearly half of the data points are from constructs considered in the service delivery lens (44%); the societal lens comprises 30% of the studies, and the system lens comprises 26% (Supplementary 4, Table S4.3). We present a summary of the results by domain and construct below (Table 3). Supplementary 4 provides specific details on the results with references to the identified studies (Supplementary Table S4.4).

**Table 3:** Synthesis of results by theoretical conceptual framework

Domain	Construct	# Records described^a^	Summary of results
Domain 1: Intervention/MPDSR	Components and execution: steps of the audit cycle described and reported on by level (a descriptive analysis)	34	Three-fourths of papers described the audit cycle (43/58); but only 17 studies described all steps of the audit cycle; half of the papers reported data collection, the review process and implementation of the recommendations (52, 53 and 52%, respectively); notification and evaluation received the least amount of attention (39% each) Literature reflects evolution of intervention over time, i.e. clinical audits to maternal and/or perinatal death reviews to MDSR to MPDSR.
	Cost relating to the audit process including collecting data, meeting related costs such as transport, specific training, running secretariat, time	34	Described as funds for training, transport and dissemination of results; human resources such as staff workload, staff shortages, staff turnover and staff skills Few studies reported on budgets and actual costs; where studied, no standard costing approach used Barrier identified as limited financial resources (without quantification)
	Framing—intervention source: ownership of implementation guideline and stakeholder perceptions on whether the intervention is externally or internally developed	41	Described as government initiated, externally driven by partners or embedded in the system One study reported as ‘top down’ approach being problematic Reported that countries adapt from the global WHO guidelines applying and adapting the recommendations to their context, but not explored Stakeholder perceptions of legitimacy not explored
	Framing—evidence strength and quality: Evidence supporting the belief that the intervention will have desired outcomes (reduced mortality; changes undertaken to improve quality of care/‘response’)	31	Described from the perspective of stakeholders that MPDSR resolves critical gaps in quality of care but little documentation of actual changes made
	Framing—relative advantage: Perception of the advantage of implementing the intervention versus an alternative solution	0	Not described
	Trialability: Ability to test/pilot the intervention on a small scale, learn and revise if warranted	22	Described a phased approach, but little documentation of learning from the phasing Identified nine pilot studies, most conducted at facility level (only one was at subnational level); no reporting of modifications or expansion after these pilots Enablers included local leadership and initial external support Barriers included sustained implementation beyond projects
	Adaptability: Degree to which an intervention can be tailored to meet the needs of an organization (core vs. peripheral elements)	15	Described as MPDSR processes adapting and changing over time but no evidence to show which factors were essential vs. peripheral to change Variations in implementation observed across facilities in same country, subnational levels and countries with different drivers of the process or frequency of review meetings
Domain 2: Individual	Technical skills and knowledge: Individual staff knowledge and competencies including skills for data collection and data use	31	Described as important to complete MPDSR process, with most studies making broad based statements about skills. Barriers included record keeping, data entry, identification and reporting of deaths, use of data for routine analysis, and familiarity with audit process Level of knowledge assessed in four studies
	Self-efficacy: Individual belief in their own capabilities to execute courses of action to achieve implementation goals.	8	Described with mixed results on individual confidence to implement MPDSR (e.g. confident or not). Enablers included supportive supervision, appropriate tools and oversight from management or health specialists.
	Individual motivation: A broad construct related to factors that motivate individuals to implement both extrinsic and intrinsic	23	Described extrinsic motivation as measures to improve quality of care, adhering to expectation from subnational teams, gaining skills or knowledge and incentives Described intrinsic motivation as consciousness for self-improvement linked to the underlying value of life Demotivating factors included lack of resources to support M/PDSR processes, lack of implementation of MPDSR-related recommendations, hierarchical nature of meetings, the process perceived as time consuming and arduous
	Individual identification with intervention: A broad construct related to how individuals perceive the intervention, and their relationship and degree of commitment to the sustained use of the intervention.	18	Described as important but not explored adequately Enablers included link between individual commitment to jobs and general quality improvement as well as individuals seeing the benefit of process improving quality over time Barrier included ‘passing the buck’ to other staff
	Individual orientation to collaboration: Personal traits such as tolerance of ambiguity, team player, flexibility, problem solving, critical thinking	0	Not described
Domain 3: Inner setting	Readiness for implementation: Tangible and immediate indicators of organizational commitment to its decision to implement an intervention	48	Enablers described as formation and or existence of MPDSR committees, a designated focal person, regularly scheduled meetings, available tools and appropriate forms for MPDSR, and ‘audit charters’, training Barriers described as shortage and capacity of health workers and disengaged leadership and inadequate management capacity
	Team composition and characteristics including who comprises the team, e.g. size, interdisciplinary nature, membership regulation	36	Described as multidisciplinary, though some studies noted low participation of nurses. Barriers identified included high staff turnover, competing priorities, lack of interest by staff and hierarchical nature of meetings.
	Organizational incentives and rewards (or disincentives/sanctions) such as goal-sharing awards, performance reviews/promotions, training, tea or the consequences	11	Enabler described as refreshments, extra training, financial motivation (per diems), and transportation. Described removal of funding that financed incentives as a demotivating factor Not adequately investigated for impact
	Team relationship: nature and quality of communication within audit team (including hierarchies, mentorship, teamwork and management)	19	Described as both positively and negatively affected by the nature of communication, collaboration, management and networking within and across teams and among stakeholders involved in the implementation process Enablers included continuous engagement, a teamwork approach, support from hospital management, invested deliberate efforts and strategies such as mentorship, as well as upholding certain norms and values to nurture a conducive atmosphere Teamwork approach involving consensus building, inclusiveness, delegation of responsibility and continuity of MPDSR as important factors Hierarchies within teams can both positively and negatively influence relationships.
	Implementation culture and climate: explanation of environment including organizational culture, learning climate, if there are things mentioned that are tensions/triggers for change	47	Enabler described as an implementation culture of accountability, learning and improvement; effective strategies included the mandatory attendance of audit meetings as well as codes of conduct or ‘audit charters’ Barriers described as a blame culture and punitive measures against frontline health providers Blame culture explored at individual level, as well as between levels of the health system and between units with mixed observations around blame-free and blame culture.
	Engaged leaders: Individuals who have formal or informal influence on the attitudes and beliefs of their colleagues with respect to implementing the intervention or on the implementation process overall, e.g. ‘champions’ or ‘agents of change’	21	Described as a critical factor for successful implementation Strong leaders are described as highly motivated individuals who can facilitate the process well Individual traits and motivations not investigated
Domain 4: Outer setting	Policy and planning: MPDSR policy and guidelines, death notification requirements, Legal mandate, litigation/legal protection	41	Described as the type of policy or guideline in place, i.e. integrated, standalone and M/PDSR related guidelines; few studies reported on the presence of a legal framework or protocol around death notification Descried as implementation factor the uptake of national policies and technical guidance and the presence of legal framework or protocol around death notification, but not explored
	Resource support: funding or resource support for MPDSR (e.g. sponsors, budgets)	29	Described as funding source, e.g. government budget line, government commitment, development partner support Barrier to implementation included lack of a budget Budgets linked to spending explored in some studies with mixed findings
	External actors: The role of external actors on the process (e.g. Local party, Union affiliations, Professional associations, Community organisations) as well as community or CHW engagement and participation in MPDSR	31	Described as the roles of key external actors, including national government, international development partners, professional associations and civil society, having influence at a subnational or facility level from strong national or subnational actors and influence at a national level from externally partners, e.g. WHO, UNFPA and donor agencies Supportive supervision reported as an implementation factor Barrier identified as absence of external actor engagement The role of development partners (UN agencies and NGOs) and professional associations at all levels described and explored, e.g. developing guidelines, training facility staff and mobilizing resources as well as pressuring governments (mostly at national level) to implement Engagements with private sector, communities, civil society and local authorities described but not explored adequately
	Political prioritization: national mobilization and awareness of issue	10	Described as pressure to implement MPDSR but not explored adequately
	Pressure: to implement from actors and other implementers	17	Described as peer pressure for system wide uptake especially from subnational structures to facility level Barrier identified as lack of national and subnational pressure to implement Perceptions around pressure to implement explored by only one study
	Linkages and networks between levels: Level of connectedness and networks with other health system levels, organizations and therefore openness or resistance to change	34	Described as the level of connectedness and networks between health system levels, different sites and different role players influences implementation Enablers identified as existing strong communication channels between and within levels; well-defined pathways around the flow of data and information relating to MPDSR; and well implemented supportive supervision Barrier identified as lack of an adequate and coherent guidance or framework to channel communication of MPDSR recommendations across levels

a See Supplementary 3, Table S3.4 for references to records by construct.

#### Domain 1: Intervention

The first domain features the intervention characteristics and process. Many studies describe the intervention components, including some elements relating to the cost of implementation, but there is no consistency in reporting all elements of the intervention process or comparable costing methods. The framing of the intervention is described as primarily externally driven; however, stakeholders view that the intervention does address critical gaps in the quality of care. While some studies report on pilots or phased implementation, few report on what has been learned from the phased approach. The breadth of the studies reflect investigations are taking place across levels and components of the intervention, with little evidence showing which factors are essential for implementation. Table 3 provides high-level findings for each construct and below details by construct.

##### Components and execution

Most studies explain the intervention process, or audit cycle in theory (74%); yet there is uneven reporting between the different components. Only 29% report on all components of the audit cycle according to the six-step audit cycle of (1) identifying cases; (2) collecting information; (3) analysing information; (4) recommending solutions; (5) implementing solutions; and (6) evaluating and refining (Kerber et al. 2015; Supplementary 4, Table S4.5). A mapping of the audit cycle steps found that over half of the studies describe the data collection process (52%), the review process (53%) and the recommendation process (52%). Fewer records report on the notification process and the evaluation of the process (or feedback loop; 40% for both).

The literature demonstrates the evolution of the intervention process over time from clinical obstetric audits to maternal and/or perinatal death reviews to MDSR to MPDSR. The studies prior to 2011 focus on maternal and/or perinatal death reviews. A WHO regional report in 2011 is the first to expand maternal death review to include surveillance (WHO 2011); another WHO regional report in 2016 is the first to present information on ‘MPDSR’ (WHO 2016b). From January 2016 until July 2018, 7 of 21 studies use the term MPDSR, though most note that the perinatal component is aspirational (WHO 2016b; Koblinsky et al. 2017; MCSP 2017c; MCSP 2017b; 2017a; Karamagi et al. 2018; MCSP 2018). Four studies, during this time period, still focus on maternal death review (without surveillance; WHO 2014a; Congo et al. 2017; de Kok et al. 2017; Du Châtelet et al. 2019). We did not find any differences in implementation factors between the different types of reviews, e.g. maternal death review, perinatal death audit, maternal death surveillance and response, or MPDSR.

##### Cost

Resources for MPDSR is recognized as an important facilitator for implementation (Kerber et al. 2015; WHO 2016b; Smith et al. 2017c); yet the literature shows mixed findings on whether specifically allocated resources are needed for MPDSR and if so, how much and how it is budgeted (Supplementary Table S4.4). Costs relating to the audit and reporting process, such as collecting data, meeting related costs (i.e. transport, specific training, running secretariat, time), information systems, etc., are often described as a barrier (Supplementary Table S4.4). Several studies specifically mention the challenge of not having funds to implement recommendations from the audit process.

Few studies report on costs, as found previously (Kerber et al. 2015), and those that do report on costs use different approaches (Pattinson et al. 2009; De Brouwere et al. 2014; Tapesana et al. 2017). The initial costs of starting MPDSR are reportedly higher than the running costs because starting requires setting up new systems and training whereas continuous costs would be nominal, such as transport to regional meetings (Grellier and Shome 2011; Nam 2011; De Brouwere et al. 2014; Biswas 2017; MCSP 2017c). The different study designs and varied contexts of the studies prevent comparability in terms of input requirements and related costs (Supplementary Table S4.4).

##### Framing

From a societal lens, implementation research theory suggests that the framing of an intervention, particularly as externally or internally developed, is critical (Damschroder et al. 2009). A study from South Africa reveals that the implementation of perinatal death audits was perceived as ‘top down’ without ownership at the facility level (Belizan et al. 2011; Bergh et al. 2011); as found in another study from Sudan (Balogun and Musoke 2014). Beyond these studies, stakeholder perceptions of legitimacy around the intervention are not explored specifically. The framing of the intervention is described in a number of studies as government initiated, externally driven by partners, or legitimate due to the embedded nature of the intervention (Supplementary Table S4.4). Some report applying and adapting national approaches from the global WHO guidelines, with two studies recognizing the importance of global guidelines in standardizing national practice (Scott and Danel 2016; Smith et al. 2017b). One study shows initiation from within a facility without influence from external partners (including Ministry of Health; Nyamtema et al. 2011).

Another framing of the intervention comes from the belief that the intervention will have the desired outcome (Damschroder et al. 2009). While studies report that MPDSR resolved critical gaps in quality of care, few document these changes with evidence beyond perceptions of those interviewed or the authors (Supplementary Table S4.4). Two studies from Ethiopia observe that once MDSR started, the level of documentation improved resulting in better communication and organized care, ultimately leading to more buy-in by stakeholders in the process (Ethiopia Federal Ministry of Health et al. 2016; Abebe et al. 2017). The final framing of the intervention considers the relative advantage of MPDSR over another process. We did not find any studies that explored perceptions of MPDSR versus other quality improvement activities.

##### Trialability

From a systems lens, the ability to test or adapt the intervention process warrants consideration. The literature reflects implementation through a phased approach, as recommended by WHO, with many studies reporting on small-scale implementation efforts (Supplementary Table S4.4). Of the nine pilot studies identified, implementation approaches and results vary (Day 2006; Dumont et al. 2009; Richard et al. 2009; Nam 2011; Nyamtema et al. 2011; Hofman and Mohammed 2014; Bayley et al. 2015; Biswas et al. 2015; Bandali et al. 2016); although local leadership and initial external support are common facilitators. Two pilots demonstrate that the death audit process can be destabilizing or even threatening, especially in settings where staff are not used to self-evaluation and critical review (Dumont et al. 2009; Richard et al. 2009). The challenge of sustained implementation beyond projects is recognized in several studies, not just the pilots (Muffler et al. 2007; Grellier and Shome 2011; Nam 2011; Hofman and Mohammed 2014; WHO 2014c).

The literature does not provide any evidence that a phased approach led to application of learning. Even when pilot experiences are very well-documented with clear lessons learned, such as FIGO LOGIC (Richard et al. 2009; Lewis 2014b; 2014a), we did not find direct application of these lessons recorded in identified studies later on. Additionally, the lessons from studies published pre-2011 (Pearson et al. 2009; Richard et al. 2009) demonstrate similar lessons learned and recommendations from studies published in the past 5 years (Scott and Danel 2016; Koblinsky 2017; MCSP 2017b; 2017a; 2017c; 2018; Du Châtelet et al. 2019).

##### Adaptability

Studies that reflect adaptability around implementation of MPDSR recognize the notion of ‘different sites, different modalities’ (Belizan et al. 2011) whereby MPDSR processes vary between facilities, subnational and national levels (Supplementary Table S4.4). Documentation of change overtime is documented in some studies, such as a shift in culture from a blame to a learning environment due to continuous and improved practice of audits overtime (Bakker et al. 2011). Observed variations include different drivers of the process (e.g. facility manager, head of department, midwife, clinical outreach person, etc.), the nature of review meetings (e.g. frequency, standalone vs. integrated, format), and composition of participants. A South African study reports that facilities determine the key role players or drivers (Belizan et al. 2011; Bergh et al. 2011). A study from Nigeria compares different MDR facility-level meetings showing that the process and approach can slightly vary due to different role players (de Kok et al. 2017). Implementation processes also vary at the national and subnational levels, including oversight and surveillance as well as national variations in processes. A study from Burkina Faso reports that the presentation of findings varied across the district level audit meetings (Congo et al. 2017). A study from South Sudan finds that the lack of the overall system being able to adapt to the local needs identified through the review process prevented uptake of MDR (Balogun and Musoke 2014). While variability between processes across facilities assumes local adaption of the intervention, we did not find any studies that identify which elements are core verses peripheral to change.

#### Domain 2: Individual characteristics

The second domain considers the characteristics of the individuals involved in implementation. Studies describe the individual’s role as important for implementation and include broad statements about the skills needed; yet few actually assess the level of knowledge required or investigate individual confidence to implement. Half of the studies consider individual motivation and identified factors that reflect both extrinsic and intrinsic motivation with some important demotivating factors identified such as hierarchy, lack of resources, lack of follow through to implement recommendations and capacity. Individual traits required for implementation are not investigated. Details by construct are below.

##### Technical skills and knowledge

Many studies acknowledge the importance of individual technical skills and knowledge to complete MPDSR processes (Supplementary Table S4.4). For the most part, these studies make broad-based statements around lack of skills as a barrier to implementation. Only four studies actually assess the level of knowledge, and their findings vary greatly as they use different methods and questions to assess technical skills and knowledge of individuals (Day 2006; Richard et al. 2009; van Hamersveld et al. 2012; Tapesana et al. 2017). Nonetheless, the literature shows that skills development takes time and goes beyond one training session. One study from South Africa reports:

*It took one day of training but on average 3–6 months before management understood the value of PPIP and up to 3 years before staff members fully appreciated the full benefit that PPIP provided to a facility* (Rhoda et al. 2014).

##### Self-efficacy

Individual confidence to implement MPDSR has mixed results depending on the study (Supplementary Table S4.4). Four studies find that staff are confident to implement with oversight (Muffler et al. 2007; Richard et al. 2009; Belizan et al. 2011; Armstrong et al. 2014); whereas other studies show mixed levels of confidence among study participants (Abebe et al. 2017; Tapesana et al. 2017) and the lack of confidence (van Hamersveld et al. 2012; Balogun and Musoke 2014). Identified enablers supporting self-efficacy include supportive supervision, appropriate tools and oversight from management or health specialists.

##### Individual motivation

*The success of audit largely depends on the motivation of the healthcare providers themselves. If they are able to evaluate the care they are giving, and willing and able to give praise where this is due, as well as make amendments where needed, then this should lead to improved motivation, ownership and sense of responsibility for delivering good quality care* (Kongnyuy and van den Broek 2009).

Studies that examine individual motivation mostly identify extrinsic factors, such as measures to improve quality of care, adhering to expectations from subnational teams, gaining skills or knowledge, or incentives (Supplementary Table S4.4). Positive outcomes from the MPDSR process also motivate health workers. The lack of resources to support MPDSR processes as well as the non-implementation of MPDSR-related recommendations is specifically cited as a demotivating factor for staff. The hierarchical nature of meetings may demotivate personnel from participating in the process in some contexts. The literature also reveals that some perceive the process as time consuming and arduous, resulting in inefficiencies in the process and lack of commitment to implement.

Intrinsic motivation described suggests that individuals find MPDSR helpful, especially for learning (Supplementary Table S4.4). Some studies reveal individual appreciation of the intervention for enabling self-reflection and self-improvement. Some argue that MPDSR is linked to professionalism of maternity care itself (Richard et al. 2009; Belizan et al. 2011; Bergh et al. 2011; de Kok et al. 2017). A study from Bangladesh demonstrates the underlying value of life as a motivator, reporting that they observe ‘one minute [of] silence for dead babies and mothers in [a] meeting’ (Day 2006). Only a few studies link individual motivation and ownership of MPDSR to inner setting elements, such as culture (de Kok et al. 2017) and team structures (Dumont et al. 2009; Kerber et al. 2015).

##### Individual identification with intervention.

While the importance of ownership and commitment to the intervention is described, few studies explore the reasons behind individual identification with the intervention (Supplementary Table S4.4). Health workers who are committed to their jobs and to quality improvement are more willing to identify with and accept MPDSR. A multi-country report from the South-East Asia Region states: *‘The commitment of physicians and supervisors is found to be a strength of the system; they have been encouraged by the fact that recommendations made at the audit meeting have been used as inputs for district planning, and have resulted in tangible improvements in the health system (WHO 2014c).’*

Ownership of the intervention can evolve over time as people see the benefits of change. One study from Ethiopia mentions a shift in individual willingness to complete case notes accurately since it was seen as having a useful purpose for MDSR rather than being an additional burden (Abebe et al. 2017). The literature supports the notion that the lack of ownership prevents effective implementation (Supplementary Table S4.4). For example, a study from Nigeria found that the lack of personal accountability for an honest process resulted in shifting responsibility or ‘passing the buck’ to other staff (de Kok et al. 2017).

##### Individual orientation to collaboration

We did not find any literature about how individual traits and critical thinking or problem solving skills support or hinder MPDSR implementation (although some aspects are described under the leadership construct).

#### Domain 3: Inner setting

The inner setting focuses on implementation factors internal to the organization. Many studies report the tangible factors required for implementation including organizational commitments and team compositions and characteristics. Organizational incentives are less reported or investigated. The nature and quality of communication within audit teams as well as the implementation climate and organization culture are identified as key implementation factors and are both positively and negatively described. Leadership is described as a critical factor for successful implementation; though individual traits and motivations are less investigated. Details by construct are below.

##### Readiness for implementation

Tangible inputs that facilitate MPDSR implementation include formation and or existence of MPDSR committees, a designated focal person, regularly scheduled meetings, available tools and appropriate forms for MPDSR, and ‘audit charters’ (Supplementary Table S4.4). The importance of training on MPDSR processes at national, subnational and facility levels is highlighted as another facilitating input in the literature.

Factors that hinder effective implementation include challenges of human resource and health management, including shortage and capacity of health workers, disengaged leadership, and inadequate management capacity. Some have argued that a minimum level of human and material resources is required before the system implements MPDSR (Muffler et al. 2007; Richard et al. 2009; Koblinsky 2017), but we did not find an agreed standard of minimum requirements in the review.

##### Team composition and characteristics

The composition and characteristics of MPDSR committees that facilitated implementation of MPDSR are described as multidisciplinary, comprising of various cadres of health workers at facility level and external stakeholders from ministries of health and MPDSR implementing partners at subnational and national levels (Supplementary Table S4.4). Restricted participation is reported as a barrier in some studies, especially when there is low participation of nurses, support staff and management. One pilot study started implementation only with the midwives and auxiliary midwives in order to establish a culture of evaluation in a blame-free setting and only broadened membership after they were comfortable with the practice (Richard et al. 2009).

##### Organizational incentives and rewards

Provision of organizational incentives, such as refreshments, extra training, financial motivations, is described mostly as an enabler although no study systematically examines the impact of such incentives. Yet still, organizational incentives are often included as recommendations for strengthening implementation, even just the provision of food or tea (Agaro et al. 2016; MCSP 2017c). Some studies recognize the negative consequences of incentives when projects are terminated, resulting in demotivation (van Hamersveld et al. 2012; Agaro et al. 2016). We did not find any studies that examined the use of sanctions for lack of implementation.

##### Team relationship

MPDSR implementation positively and negatively affects and is affected by the nature of communication, collaboration, management and networking within and across teams and among stakeholders involved in the implementation process. Many studies describe the team relationships among health facility staff and surrounding clinics (Supplementary Table S4.4). Identified approaches that nurture team relationships include continuous engagement, a teamwork approach, support from hospital management, deliberate efforts and strategies, such as mentorship, as well as upholding certain norms and values to create a conducive atmosphere. Two studies report that the MPDSR process itself nurtured team spirit and collaboration (Purandare et al. 2014; WHO 2014b). For example, ‘sharing regular updates on the program’s progress ensured timely help and kept the team motivated to deliver high-level performance (Purandare et al. 2014)’. In contexts where a teamwork approach to implementing MPDSR was adopted, studies report that there was consensus, inclusiveness, monitoring of staff performance, delegation of responsibility and continuity of the MPDSR implementation processes. Strong communication, involvement and support from hospital management are also found to strengthen team relationships for MPDSR.

Studies also report that the lack of management, communication and coordination across teams, including poorly functioning teams are formidable barriers (Supplementary Table S4.4). Three studies report that the existence of hierarchies within teams and across various contexts have positively influenced team relationships through provision of leadership and mentorship (Dumont et al. 2009; Bakker et al. 2011; de Kok et al. 2017). However, more records show the negative influence of professional hierarchies between health cadres, notably the silencing of the more junior staff and nurses in the process (Supplementary Table S4.4). Structural hierarchies may also constrain the performance of teams in cases where the senior members are absent or unable to perform their duties. Not many studies examine the effect of hierarchies and teamwork on implementation of MPDSR beyond the health facility level. The few that do, only describe the institutional reporting structures rather than the inner team dynamics (MCSP 2017b; 2017a; 2017c; 2018).

##### Implementation culture and climate

Some studies demonstrate how MPDSR functions well in settings with a culture of accountability, learning and improvement (Supplementary Table S4.4). A culture of trust is nurtured by strong leadership and continuous reassurance of a ‘blame-free culture’ (Belizan et al. 2011; Grellier and Shome 2011; Kerber et al. 2015; Du Châtelet et al. 2019). Open and enabling environments, which encourage active participation of all participants during meetings, are reported to improve implementation (Dartey 2012; MCSP 2017a). Some studies provide useful resources and tips on how to promote positive culture for MPDSR implementation (Dumont et al. 2009; Richard et al. 2009; Lewis 2014b).

On the opposite spectrum, a blame culture and punitive measures against frontline health providers are widely recognized as barriers to the implementation of the MPDSR (Supplementary Table S4.4). The most common reasons cited are feeling threatened during the review meetings and fearing legal action or punitive repercussions; although some records note a general culture of blame around MDPSR (Koblinsky et al. 2017; MCSP 2017a). The continued prevalence of the blame culture may partially be attributed to lack of clarity around the process when first implemented (van Hamersveld et al. 2012; Agaro et al. 2016; Du Châtelet et al. 2019). In some cases, there are mixed results whereby different individuals report different experiences, with some reporting a blame-free culture and others not. A few studies report shifts in culture from a blame to a learning environment due to continuous and improved practice of audits overtime (Bakker et al. 2011; Belizan et al. 2011; Bergh et al. 2011). The blame culture is reported mostly at the facility level with a focus on individuals or teams e.g. different health cadres or units (obstetrics vs. pediatrics). Only one study reported on blame culture across districts (Congo et al. 2017); blame culture and its effect at the subnational and national levels is not adequately studied (de Kok et al. 2017). At the facility level, identified strategies to minimizing acrimony, avoiding blame and recriminations include the mandatory attendance of audit meetings as well as codes of conduct or ‘audit charters’ (Dartey 2012; MCSP 2017a; 2017b; Richard et al. 2009; Dumont et al. 2009; Lewis 2014b).

Another factor identified as contributing to the fear among health workers is the absence of a strong MPDSR legal framework across all levels (WHO 2014c; Agaro et al. 2016; Koblinsky et al. 2017), although the explicit aspects of fear about litigation are not described or explored. Amidst this however, studies also describe fear for litigation as having a positive effect on the implementation climate as a form of accountability (Bakker et al. 2011; Abebe et al. 2017; MCSP 2017c).

##### Engaged leaders

Strong leadership is described as a critical factor for successful implementation of MPDSR, with some studies showing positive influence while others note the lack of leadership as a barrier (Supplementary Table S4.4). The importance of leadership as an implementation factor cross cuts the levels of the health system. At a national level, change agents may include individuals within the Ministries of Health, professional associations and partners such as UNFPA, WHO. At a subnational level, the buy in and dedication to MPDSR by district managers can support or hinder implementation. At facility level, change agents include individual health workers or teams; who have additional responsibilities, such as being in-charges of department/units.

A few studies describe the attributes of strong leadership, their critical tasks and/or the perceived quality of leaders for MPDSR (Bakker et al. 2011; Belizan et al. 2011; Bergh et al. 2011; Lewis 2014b; Rhoda et al. 2014; WHO 2014a; 2014c). Champions or engaged leaders are described as highly motivated individuals (Supplementary Table S4.4) but no study specifically explores their motivations. Five studies highlight the important role of facilitation in terms of having a good chairperson or a person who is able to steer the conversation to be blame-free and productive (Dumont et al. 2009; Richard et al. 2009; Bakker et al. 2011; Hofman and Mohammed 2014; de Kok et al. 2017). As noted in one study, ‘“true leaders” of the audit session … usually are the first to ask questions and start discussions (Bakker et al. 2011).’

#### Domain 4: Outer setting

The outer setting includes factors external to the organization that influence implementation of MPDSR. The tangible inputs, such as policies and legal frameworks or resource support, are mentioned in a number of studies but their actual impact is not explored. Many studies reveal the influence of external actors on implementation at multiple levels, and this also links to the pressure to implement, though perceptions around external pressure is rarely reported. The response component of MPDSR, a key purpose to the intervention, requires linkages and communication across and between levels of the health system; therefore, this is a key area described in the literature with enablers and barriers identified for improving implementation.

##### Policy and planning

Policy and planning for MPDSR include related guidelines, national plans, death notification requirements and legal protection. Studies report various approaches, such as integrated policies or standalone national policies on maternal and/or perinatal deaths notification or national MPDSR related guidelines, with lack of national guidelines hindering implementation (Supplementary Table S4.4). There has been an increase in the number of countries with policies overtime, yet the limitations of the global tracking process is recognized as not sufficient for measuring implementation of MPDSR (Bandali et al. 2016; Kerber et al. 2015; Magoma et al. 2015; Smith et al. 2017b; Muffler et al. 2007; Pearson et al. 2009). Specific benefits of having a national guidelines identified in the literature include unifying fragmented MPDSR-related processes, institutionalizing practice and informing the implementation process, e.g. how to set up a committee.

Some studies describe the presence of a legal framework or protocol around maternal and perinatal death notification, which obligates clinicians and managers to report on the deaths to a central system (Supplementary Table S4.4). Obligatory notification may demonstrate maternal mortality as a government priority adding additional pressure on practitioners (Scott and Danel 2016; Mutsigiri-Murewanhema et al. 2017). Legal measures linked to the MPDSR process, particularly around liability and punitive measures, may also hinder implementation (see inner setting; Lewis 2014a; Hadush et al. 2016; Koblinsky 2017). Only one study discusses the types of legal frameworks or safeguards required for MPDSR (Smith et al. 2017c); a brief by E4A further describes types of legal frameworks (E4A 2012).

##### Resource support

We consider the source of funding as an external influence on implementation; whereas the actual costs are described under the intervention domain. Settings with established MPDSR related processes report government financial support, such as in Malaysia and South Africa (Pearson et al. 2009; Bandali et al. 2016; Koblinsky et al. 2017; Smith et al. 2017c). A national budget line for MPDSR also shows promise in studies from Burkina Faso, South Africa, Sri Lanka and Indonesia (Belizan et al. 2011; Bergh et al. 2011; Rhoda et al. 2014; WHO 2014b; Bandali et al. 2016; Congo et al. 2017; Koblinsky et al. 2017). Though the lack of budgets for MPDSR at various levels is described generally as a barrier; national or regional budgets specific to MPDSR do not necessarily increase spending for MPDSR as found in studies from Nigeria, Tanzania and Indonesia (Magoma et al. 2015; Koblinsky et al. 2017; MCSP 2017b). The link between levels of funding and political commitment as well as buy-in from government to maternal and newborn health is acknowledged (Pearson et al. 2009; Abebe et al. 2017; Smith et al. 2017c), but not studied. One study finds MPDSR itself is used to mobilize resources for the process (Hofman and Mohammed 2014). The literature recognizes the importance of international mobilization of resources for MPDSR, and that dependence on development partners cannot sustain practice, as reflected in this quote:

*Without government commitment and funds to scale-up, countries are unable to continue strengthening capacity of staff at all levels to conduct MDR – i.e. training on the MDR method in all facilities, and training for assessors on completing MDR forms, maternal death classification (using ICDMM) and formulating recommendations* (Smith et al. 2017c).

##### External actors

From a societal lens, the influence of external actors on the implementation of MPDSR are widely discussed or observed in the literature with varying findings by study including scope and level of engagement (see mapping in Supplementary File 4, Table S4.4). At a subnational or facility level, strong national actors influence implementation through ministries of health, often with a strong national committee. At a national level, there is a critical role of WHO, UNFPA and donor agencies. At all levels, many studies report that development partners (UN agencies and NGOs) and professional associations play a role in both supporting implementation processes, e.g. developing guidelines, training facility staff and mobilizing resources as well as pressuring governments (mostly at national level) to implement. The absence of external actor engagement may also imped implementation. Though not investigated in the studies identified in this review, arguments are made for benefiting from engagement with private sector, community, professional associations and others (Pearson et al. 2009; Bayley et al. 2015; Kerber et al. 2015; Hadush et al. 2016; Du Châtelet et al. 2019) as well as cautioning against expanding external engagement (Bandali et al. 2016; Ministry of Health and Sanitation [Sierra Leone] 2017), such as to private sector (Balogun and Musoke 2014), communities, civil society and local authorities (Tapesana et al. 2017), partially due to legal risks (WHO 2014c; Du Châtelet et al. 2019).

Different types of community links to facility-based MPDSR are mentioned but not studied. Low levels of community engagement or participation in the MPDSR process are proposed barriers of implementation, with specific challenges noted around data collection of deaths in the community (Supplementary Table S4.4).

##### Political prioritization

National political commitment and government leadership are possible pressures on the health system to implement MPDSR. Gaps relating to actual political prioritization of MPDSR remain glaring for some in terms of inadequate funding for MPDSR across all health system levels (Agaro et al. 2016; Du Châtelet et al. 2019). While global commitments to development goals or regional commitments may have led to additional pressure on national governments to implement (WHO 2013b; Kerber et al. 2015; Bandali et al. 2016), this is not systematically assessed in the literature.

##### Pressures to implement

From a systems lens, few studies specifically look at perceptions around pressure to implement. Regular reporting to WHO and other agencies on policy uptake could be seen as a form of pressure or accountability, but this is not studied. At the facility level, peer pressure for system wide uptake came in the form of outreach visits from regional specialists and reporting requirements by subnational structures. It can also be from within the team (Supplementary Table S4.4). The lack of national and subnational pressure to implement is recognized as barrier to implementation (Nam 2011; Balogun and Musoke 2014).

##### Linkages and networks between levels

The level of connectedness and networks between health system levels, different sites and different role players influences implementation. Overall, the literature describes communication across levels, e.g. notification forms shared, dissemination of findings and actions, data and information shared through clear communication channels (Supplementary Table S4.4). When functional, MDPSR processes appear to strengthen communication across the levels of the health system and between stakeholders. Supportive supervision serves as a link between levels, but its influence depends on the actors, approach and context. Existing strong communication channels and well-defined pathways around the flow of data and information relating to MPDSR support implementation. A study from Ethiopia shows that better data and reporting improves communication across the health system as well as between team members (Abebe et al. 2017). Effective dissemination of the benefits that MPDSR implementation achieves can be a trigger for change (Lewis 2014b), but further research is needed (Dumont et al. 2009).

Some studies find that the absence of an adequate and coherent framework to guide both local and national communication and dissemination of MPDSR recommendations can be a barrier to implementation. As a result, there exists lack of clarity of roles and duplication of activities among stakeholders at the subnational and national levels in some settings (WHO 2014c; MCSP 2018; Du Châtelet et al. 2019). The lack of connectivity is also identified as a barrier to implementation in other studies, even when systems and guidelines were in place.

The linkage to existing health system structures may also influence implementation, such as integrating surveillance into other health programming or integrating activities into other maternal and newborn health programmes (Pearson et al. 2009; Bandali et al. 2016; Abebe et al. 2017). Vertically designed programmes prevent uptake and sustainability, as demonstrated in a study from Sudan (Balogun and Musoke 2014).

## Discussion

This scoping review reveals the complexity of MPDSR as an intervention process requiring many steps, engagement of multiple individuals with differing roles, and information sharing across levels of the health system. The review also shows that research on MPDSR implementation is growing in LMIC settings, especially in Africa. Many of the studies describe the ‘hardware’ or tangible inputs to MPDSR implementation, which have been previously recognized (Pattinson et al. 2005; Pattinson et al. 2009; De Brouwere et al. 2014; Lewis 2014a; Hussein et al. 2016; Martin et al. 2016; Scott and Danel 2016; Biswas 2017). Among the fewer studies that explore the ‘software’ elements in the health system, such as the power dynamics, ideas, values and norms (Sheikh et al. 2011), it is clear that people, their relationships and communication channels are at the heart of the implementation process; yet their subjective experiences and relationships are inadequately focused on in the literature. The complex interplay and change dynamics of MPDSR implementation, such as the pressures or underlying motivations behind why people implement or not, require further research. In an effort to unpack the complexities of the MPDSR implementation process, we discuss the findings according to each lens: service delivery, societal and systems (Figure 1; George et al. 2019).

### Service delivery lens: inputs that are needed for implementation

Tangible inputs required for implementation include skills and knowledge of the individuals involved, policies and guidelines, system inputs, trainings and consideration of its costs and resource support. The review confirms the importance of staffs’ technical knowledge around how to implement MPDSR (Kongnyuy and van den Broek 2009; Raven et al. 2011; Hussein et al. 2016; Biswas 2017), but we did not find a list of required competencies needed at technical and management levels for implementation or many investigations into individual competencies.

The review also validates the already identified system inputs e.g. focal person, committees, multidisciplinary teams, regularly scheduled meetings, available tools, audit charters, training, human resource (Pattinson et al. 2005; Pattinson et al. 2009; De Brouwere et al. 2014; Hussein et al. 2016; Martin et al. 2016; Biswas 2017). Organizational incentives require further investigation to look at impact (positive/negative) in different contexts. Securing funds for the implementation of MPDSR as a process as well as to finance the response activities is needed to sustain implementation (Kerber et al. 2015); yet there is no standard approach to costing the intervention. Gaps in knowledge still exist on the actual cost of audit teams meeting, the opportunity cost to people involved in an audit, the cost of collecting data, data analysis, conducting MPDSR training and running a secretariat. South Africa is the only country with literature on the cost of the national perinatal death audit programme, as well as guidance on how to allocate resources for the implementation process to function (Pattinson et al. 2009; Baleta 2011).

The tracking of policies and guidelines, including legal frameworks and protocols around death notification may be helpful (Martin et al. 2016), but policy analyses are also needed to strengthen implementation efforts and address gaps. The global WHO guidelines and related support mechanisms, such as the regional technical meetings, may also influence standardizing and improving national MPDSR process, but these have not been studied for impact on implementation. The literature also does not systematically report on all steps of the audit cycle, with most studies focusing on different components of the intervention and only a few studies attempting to verify and measure the full intervention process. If the audit cycle must be completed and effectively implemented overtime in order to trigger iterative cycles of improvement and improve outcomes (Pattinson et al. 2009; Kerber et al. 2015), then further study of the complete audit cycle will be required to identify implementation factors for the overall process and measure impact. Implementation of the ideal format, as promoted by WHO and national guidelines, is not adequately documented or reported on in the studies, though the review confirms that countries not implementing according to the WHO or national guidelines (Martin et al. 2016; Lusambili et al. 2019). Part of the challenge perhaps is that the MPDSR process varies by level, by intervention step, by time point in the evolution process (Lewis 2014a; Koblinsky 2017), making it difficult to measure. The continuous adaptation to the intervention itself, evolving from facility death review, MDSR, MPDSR is also recognized (Lewis 2014a; Biswas 2017; Koblinsky 2017), but not been studied. For example, perinatal death audits and notification seem to have taken on a similar shape as MDSR, where reported, but actual implementation of ‘MPDSR’ (with demonstration of the perinatal and surveillance components) appears nascent in the identified studies. The central question of what are core elements of MPDSR versus the adaptable periphery is not answered by any of the literature.

### Societal lens: the interactions between those involved in the implementation

The review shows the important role of external actors at all levels, especially in terms of developing guidelines and implementation support and funding (Pattinson et al. 2009; De Brouwere et al. 2014; Lewis 2014a; Martin et al. 2016; Biswas 2017; Koblinsky 2017). External influence, either from development partners or through the health system (e.g. national influence on subnational implementation), has previously been linked to the legitimacy of MPDSR (Pattinson et al. 2009; De Brouwere et al. 2014; Lewis 2014a; Hussein et al. 2016; Scott and Danel 2016; Biswas 2017; Koblinsky 2017; Smith et al. 2017a); yet these links and the nature of external actor involvement require more systematic investigation and likely depend on context. For example, a country with greater political prioritization of maternal and perinatal health may lead to more external pressure on those who are implementing MPDSR, as demonstrated by a recent study from Ethiopia (Melberg et al. 2019).

Successful implementation of MPDSR requires an individual’s willingness to ‘self-correct’ (Pattinson 2005), and commitment of staff to conducting audit themselves, to accept open discussion with peers and to take forward the actions recommended (Johnston et al. 2000; Pattinson et al. 2005; van Hamersveld et al. 2012). The literature reflects the importance of individual perspectives, values, experiences and motivation as implementation factors. We found that the outcome of MPDSR influences perception about the intervention, including buy-in to the belief that the intervention will make a difference. Previous reviews and commentaries have also described evidence of the impact of MPDSR as an implementation factor (Pattinson et al. 2005; Kongnyuy and van den Broek 2009; Pattinson et al. 2009; Raven et al. 2011; Buchmann 2014; De Brouwere et al. 2014; Lewis 2014a; Hussein et al. 2016; Biswas 2017; Smith et al. 2017a). Likewise, self-efficacy is a critical component in most individual behavior change theories (Damschroder et al. 2009), but it is understudied for MPDSR.

Lewis (2014a) argues that an environment open to learning requires individual responsibility and ownership of the process, whereby clinicians need to want to improve their practice and change their behaviour for the betterment of maternal and perinatal health. Our review shows that individuals found the MPDSR process to be helpful, especially for learning, a first step towards individual change. Factors that build individual confidence to implement MPDSR align with other quality improvement efforts for maternal and newborn health, such as supportive supervision, appropriate tools and oversight from subnational management or health specialists (Raven et al. 2011; Kerber et al. 2015; Zamboni et al. 2019).

Kongnyuy and van den Broek (2008) claim ‘the success of audit largely depends on the motivation of the healthcare providers themselves.’ The review supports this theory. Extrinsic motivation, such as expectations from subnational teams, skills or knowledge and incentives, improved quality as well as intrinsic motivation, such as consciousness for self-improvement and value of life, play a role. Individual motivation and buy-in also relates to ownership of the implementation as individuals see the benefits of change overtime (Baleta 2011; Lewis 2014a; Koblinsky 2017). Beyond users, maintaining stakeholder confidence and commitment has been recommended for implementation (Hadush et al. 2016), but this has not been studied for MPDSR, specifically.

MPDSR is often included as part of a package of interventions implemented for testing or strengthening quality improvement efforts, as in the QUARITE Trial identified in this review (Dumont et al. 2013). Since MPDSR, or any form of maternal and perinatal death review or audit, often falls under clinical governance (McSherry and Pearce 2011), audit becomes one of the multiple tools and practices used as a measure for and means to improve quality of health care (Amelia et al. 2015). It acts as a trigger to facilitate behaviour change at the provider level (Bauer et al. 2015). Therefore, the presumption that MPDSR should be implemented along with other clinical governance practices is supported (Pattinson et al. 2005; Kongnyuy and van den Broek 2009; Pattinson et al. 2009; Raven et al. 2011), even though relative advantage has not been established. However, there is very little research or documentation of how MPDSR relates to ongoing quality improvement processes and what health workers see as the relative advantage (Mukinda et al. 2020a; 2020b).

The nature and quality of communication within teams, such as hierarchies, mentorship, teamwork, and management, also reveal to be an important determinant of implementation (Raven et al. 2011; Hussein et al. 2016; Koblinsky 2017). The effects of these components vary across different contexts within communities as well as across different levels of the health system. For example, the review found that there are both positive and negative influences of hierarchies on MPDSR implementation, even if not investigated in depth. Hierarchies relate to leadership approaches, and optimal teamwork relies on effective leadership approaches that create an enabling environment (Cornthwaite et al. 2013; Gilson 2016).

### Systems lens: things that trigger change

Proven quality improvement interventions depend on an enabling environment at the national, subnational, and facility-levels with consideration of both everyday culture and broader healthcare improvements (Mensah Abrampah et al. 2018; Zamboni et al. 2019). MPDSR is considered an accountability mechanism (Martin et al. 2016) as well as a pathway towards individual and collective accountability (Johnston et al. 2000; Pattinson et al. 2005; O'Hagan and Persaud 2009; van Hamersveld et al. 2012). Even though fear of blame is a widely recognized barrier to implementation (Kongnyuy and van den Broek 2009; Raven et al. 2011; Lewis 2014a; Scott and Danel 2016), our review exposes the complexity of blame, including different explanatory reasons for it and different types. Future research needs to go beyond identifying blame as a barrier to understanding how to create a culture of accountability, learning and improvement through strengthening leadership, improving teamwork and communication, driving motivation while considering context (Khatri et al. 2009). More focus on investing in and researching the software elements of the health system may support an effort towards a no-blame, no-shame implementation environment (Sheikh et al. 2011; Lusambili et al. 2019). Using theory allows for exploration of issues, such as trust, credibility and hierarchies shaped by the power relations between MPDSR stakeholders, and have been used by others when investigating quality improvement processes (Hulscher et al. 2013; Davidoff et al. 2015; Akachi and Kruk 2017; Kruk et al. 2017; Persson 2017; Topp 2017), including audit and feedback (Ivers et al. 2012).

Often described as the most influential factor in shaping organizational culture, effective leadership is critical at all levels (Mathole et al. 2018). Though engaged leaders are widely recognized as an enabler, we did not find much literature specifically looking at the necessary individual leadership traits and critical thinking or problem solving skills needed for MPDSR. Skills in facilitation are one trait identified but not specifically investigated. For successful MPDSR implementation, more needs to be understood on what motivates these leaders, what skills are needed, how to grow new champions. There is a wealth of knowledge already about leadership in health (Gilson 2016; Mathole et al. 2018), which may be applicable to MPDSR.

The complex interplay of connectedness and networks between health system levels, different sites and different role players influences MPDSR implementation (Raven et al. 2011; Lewis 2014a). Connected systems with clear channels of communication, a clear pathway of information flow, as well as accountability mechanisms, such as supportive supervision, enable completion of the audit cycle. Not only is this important for implementation of MPDSR, but operational feedback loops also encourage individual commitment to the process as more stakeholders come to see the benefits of MPDSR. The review finds that subnational structures play a vital role in implementation for accountability and quality control (e.g. supportive supervision; clear pathway of information flow); yet few studies investigate their role and influence. A governance perspective more broadly for maternal and newborn health, especially at the meso-level of the health system, may be useful in helping to strengthen implementation (George et al. 2019; Mukinda et al. 2020b; Schneider et al. 2020). Especially as one must also take into account that MPDSR is among many other accountability or quality improvement initiatives being implemented (Mukinda et al. 2020a). MPDSR cannot be a short-term investment or a vertical intervention to promote. Successful implementation of this complex intervention process is linked to other health system strengthening efforts (Dumont et al. 2013) but these linkages appear to be understudied.

WHO guidelines encourage learning from past and current experiences to inform the future of MPDSR implementation (WHO 2016c; Koblinsky 2017). While a phased approach is widely promoted (Kongnyuy and van den Broek 2009; Pattinson et al. 2009; De Brouwere et al. 2014; Lewis 2014a; Hussein et al. 2016; Biswas 2017; Koblinsky 2017), the lack of literature on how learning from pilots or a phased implementation approach leads to improved implementation efforts is of concern, especially given the findings around the influence of external actors. Future implementation and research on MPDSR may also benefit from considering the vast literature more broadly on adaptability and sustainability in development (Bopp et al. 2013; Spicer et al. 2018; Zamboni et al. 2019).

### Applying an implementation framework

The Consolidated Framework for Implementation Research served as a useful framework to understand the complex nature of MPDSR by allowing us to consider the different levels and different factors that influence implementation (Kinney et al. 2019). By collapsing the intervention and the process together as one domain, we were able to reduce some of the overlap in concepts applicable to MDPSR. Incorporating the three lenses—service delivery, societal and systems—furthered our ability to understand the different approaches and measurement of implementation factors from a health systems perspective. The review found that implementation frameworks and health systems thinking are rarely used in the original studies and reports, therefore our application of the framework required significant interpretation by the study team and continuous reflection and discussion. Using frameworks from implementation and health systems research in understanding complex health intervention processes, such as MPDSR, will create much more room for growth in future studies, as flagged by the numerous gaps found by applying such frameworks.

## Limitations

The literature on MPDSR is vast and complex with different terminologies used to describe the intervention. At the time of the review, there was no standard definition or reporting global guidelines on how to describe MPDSR, we use the WHO definitions and guidelines for maternal death review, maternal death surveillance and response, and perinatal death audit (WHO 2004; WHO 2013a; WHO 2016a). Despite our attempt to capture related processes, referred to as obstetric audits, clinical audits or facility-based maternal and perinatal morbidity and mortality audits, some relevant literature may have been missed in the search. The inclusion criteria excluded confidential inquires, maternal near-miss reviews, verbal autopsies and social autopsies (Lewis 2014a); and we recognize that many of the elements central to this review may also be relevant to this literature. Much of the MPDSR-related literature looked at outcomes of the intervention, such as causes of death, modifiable factors and recommendations, and therefore, it took time to identify articles that document the actual implementation process as some studies included this information but it was not a main objective of the study. The scoping review is limited by language and time span but it is comprehensive in the inclusion of grey literature through consultation with experts in the field. While we present quantifications to characterize the literature, e.g. number of pilot studies, the decision-making, abstraction and interpretation of findings is subjective. In addition, the development and application of the implementation framework required continuous discussion and revisions by the team. The team had regular meetings to discuss our understanding of the concepts and documented our decisions.

## Conclusion

This scoping review identifies and describes implementation factors relating to MPDSR in LMIC settings applying an implementation framework and health systems thinking, allowing for deeper understanding of implementation. The literature mostly identifies factors influencing implementation related to MPDSR as an intervention and its inner and outer setting, with less attention to the individuals involved. Much attention is paid to implementation factor involving tangible inputs from the service delivery lens; however, we found no agreed minimum requirements or standard approach to measuring implementation of these components. Though less studied, the societal and health systems implementation factors show that people (external actors, leaders and team members), their relationships, their motivations, their implementation climate and their ability to communicate influence implementation processes; yet their subjective experiences and relationships are inadequately focused on in the current literature. MPDSR implementation benefits from a culture of accountability, learning and continuous improvement as well as contributes to accountability at all levels; but few have studied the complex interplay and change dynamics of implementation in relation to other quality improvement and accountability mechanisms. Understanding of MPDSR will require more research using health policy and systems approaches, including the use of implementation frameworks.

## Supplementary data

Supplementary data are available at *Health Policy and Planning* online

## Supplementary Material

czab011_Supp

## Data Availability

The data underlying this article will be shared on reasonable request to the corresponding author. **Box 1** Overview of the conceptual implementation framework for MPDSR The theoretical conceptual framework developed for this review is adapted from the Consolidated Framework for Implementation Research (Damschroder et al. 2009), and well described in the protocol paper (Kinney et al. 2019). The visual of the framework (Figure 1) shows that MPDSR functions at multiple levels of the health system—national, subnational, facility (and for some countries community level components are included in the process). The communication system and interconnectedness between the different levels is an important component of M/PDSR since the process is a reporting mechanism moving continuously from bottom up—facility to national—and also from top down—national to facility. It also shows that there is MDPSR in theory, e.g. how it should work based on guidelines, and that there is MPDSR in practice, e.g. how it actually works. The framework includes three different lenses through which to understand and measure health system drivers of women’s and children’s health (George et al. 2019). A service delivery lens includes the tangible inputs needed for MPDSR implementation; a societal lens includes constructs that focus on social understanding and relationships; and a systems lens includes constructs that emphasis change dynamics, which includes adaptive learning to contexts in ways that are not always anticipated. The factors within each domain are categories by lens, which are denoted by grey-shading in the figure. The framework considers four domains with 24 constructs in total: Intervention: The first domain is MPDSR or any related form of maternal and/or perinatal death review or audit. Factors within this domain for MPDSR include the components of the audit cycle and costs relating to the audit process from a service delivery lens, framing of the intervention source, evidence strength and quality and relative advantage from a societal lens, and the perceived ability to test and adapt it from a systems lens. Individual: The next domain considers the characteristics of the individuals involved in implementation. From a service delivery lens, factors include their technical skills and knowledge; from a societal lens, factors include their self-efficacy, motivations and identification with the intervention; and from a systems lens, factors include their ability to move from orientation to collaboration. Inner setting: The third domain considers factors internal to the organization. From a service delivery lens, this includes the readiness to implement, team composition and characteristics, and incentives to implement; from a societal lens, this includes team relationships; and from a systems lens, this includes the organizational culture and implementation climate, and engagement of leaders (often called ‘champions’). Outer setting: The final domain considers factors external to the organization that influence implementation of MPDSR. These factors include policy and planning and resource support or funding for MPDSR from a service delivery lens; the role of external actors (such as professional association) and political prioritization from a societal lens; and from the pressures to implement and the linkages and networks between levels from a systems lens. Supplementary 2 further describes the framework and includes an overview of how the framework was adapted and evolved during the data extraction and analysis process of the scoping review.

## References

[czab011-B1] Abebe B , Busza J, Hadush A et al 2017. ‘ We identify, discuss, act and promise to prevent similar deaths’: a qualitative study of Ethiopia's Maternal Death Surveillance and Response system. BMJ Global Health 2: e000199. 10.1136/bmjgh-2016-000199 PMC5435261 28589016

[czab011-B2] Agaro C , Beyeza-Kashesya J, Waiswa P et al 2016. The conduct of maternal and perinatal death reviews in Oyam District, Uganda: a descriptive cross-sectional study. BMC Womens Health 16: 38. 27418127 10.1186/s12905-016-0315-5 PMC4944522

[czab011-B3] Akachi Y , Kruk ME. 2017. Quality of care: measuring a neglected driver of improved health. Bulletin of the World Health Organization 95: 465–72. 28603313 10.2471/BLT.16.180190 PMC5463815

[czab011-B4] Amelia D , Suhowatsky S, Baharuddin M et al 2015. Case study: clinical governance as an approach to improve maternal and newborn health in 22 hospitals in Indonesia. World Health & Population 16: 16–23. 26860759 10.12927/whp.2016.24497

[czab011-B5] Arksey H , O'Malley L. 2005. Scoping studies: towards a methodological framework. International Journal of Social Research Methodology 8: 19–32.

[czab011-B6] Armstrong CE , Lange IL, Magoma M et al 2014. Strengths and weaknesses in the implementation of maternal and perinatal death reviews in Tanzania: perceptions, processes and practice. Tropical Medicine & International Health 19: 1087–95. 25039579 10.1111/tmi.12353

[czab011-B7] Bakker W , van den Akker T, Mwagomba B et al 2011. Health workers' perceptions of obstetric critical incident audit in Thyolo District, Malawi. Tropical Medicine & International Health 16: 1243–50. 21767335 10.1111/j.1365-3156.2011.02832.x

[czab011-B8] Baleta A. 2011. South Africa takes steps to reduce perinatal mortality. The Lancet 377: 1303–4. 10.1016/s0140-6736(11)60523-0 21504086

[czab011-B9] Balogun HA , Musoke SB. 2014. The Barriers of Maternal Death Review Implementation in Sudan - A Qualitative Assessment. Masters of Medical Science, Karolinska Institutet.

[czab011-B10] Bandali S , Thomas C, Hukin E et al 2016. Maternal Death Surveillance and Response Systems in driving accountability and influencing change. International Journal of Gynecology & Obstetrics 135: 365–71. 27836470 10.1016/j.ijgo.2016.10.002

[czab011-B11] Bauer MS , Damschroder L, Hagedorn H et al 2015. An introduction to implementation science for the non-specialist. BMC Psychology 3: 32. 26376626 10.1186/s40359-015-0089-9 PMC4573926

[czab011-B12] Bayley O , Chapota H, Kainja E et al 2015. Community-linked maternal death review (CLMDR) to measure and prevent maternal mortality: a pilot study in rural Malawi. BMJ Open 5: e007753. 10.1136/bmjopen-2015-007753 PMC4410129 25897028

[czab011-B13] Belizan M , Bergh AM, Cilliers C et al, the Synergy Group. 2011. Stages of change: a qualitative study on the implementation of a perinatal audit programme in South Africa. BMC Health Services Research 11: 243. 21958353 10.1186/1472-6963-11-243 PMC3195711

[czab011-B14] Bergh AM , Pattinson R, Belizan M et al, for the Synergy Group. 2011. Completing the Audit Cycle for Quality Care in Perinatal, Newborn and Child Health. Pretoria: Medical Research Council of South Africa.

[czab011-B15] Biswas A. 2017. Shifting paradigm of maternal and perinatal death review system in Bangladesh: a real time approach to address sustainable developmental goal 3 by 2030. F1000Research 6: 1120. 28944044 10.12688/f1000research.11758.1 PMC5585875

[czab011-B16] Biswas A , Rahman F, Eriksson C et al 2015. Facility death review of maternal and neonatal deaths in Bangladesh. PLoS One 10: e0141902. 26540233 10.1371/journal.pone.0141902 PMC4634754

[czab011-B17] Bopp M , Saunders RP, Lattimore D. 2013. The tug-of-war: fidelity versus adaptation throughout the health promotion program life cycle. The Journal of Primary Prevention 34: 193–207. 23526141 10.1007/s10935-013-0299-y

[czab011-B18] Buchmann EJ. 2014. Towards greater effectiveness of perinatal death audit in low- and middle-income countries. BJOG 121 Suppl 4: 134–6. 25236647 10.1111/1471-0528.12904

[czab011-B19] Congo B , Sanon D, Millogo T et al 2017. Inadequate programming, insufficient communication and non-compliance with the basic principles of maternal death audits in health districts in Burkina Faso: a qualitative study. Reproductive Health 14: 121. 28969656 10.1186/s12978-017-0379-1 PMC5623962

[czab011-B20] Cornthwaite K , Edwards S, Siassakos D. 2013. Reducing risk in maternity by optimising teamwork and leadership: an evidence-based approach to save mothers and babies. Best Practice & Research Clinical Obstetrics & Gynaecology 27: 571–81. 23647702 10.1016/j.bpobgyn.2013.04.004

[czab011-B21] Damschroder LJ , Aron DC, Keith RE et al 2009. Fostering implementation of health services research findings into practice: a consolidated framework for advancing implementation science. Implementation Science 4: 50. 19664226 10.1186/1748-5908-4-50 PMC2736161

[czab011-B22] Dartey AF. 2012. The Role of Midwives in the Implementation of Maternal Death Review (MDR) in Health Facilities in Ashanti Region, Ghana. Belville: University of the Western Cape.

[czab011-B23] Davidoff F , Dixon-Woods M, Leviton L et al 2015. Demystifying theory and its use in improvement. BMJ Quality & Safety 24: 228–38. 10.1136/bmjqs-2014-003627 PMC4345989 25616279

[czab011-B24] Day LT. 2006. Evaluation of Perinatal and Maternal Death Audit in Pilot Trained Facilities in Bangladesh. Dinajpur: Internal report for Saving Newborn Lives, Save the Children.

[czab011-B25] De Brouwere V , Delvaux T, Leke RJ. 2014. Achievements and lessons learnt from facility-based maternal death reviews in Cameroon. BJOG 121 Suppl 4: 71–4. 25236637 10.1111/1471-0528.12902

[czab011-B26] de Kok B , Imamura M, Kanguru L et al 2017. Achieving accountability through maternal death reviews in Nigeria: a process analysis. Health Policy Plan 32: 1083–1091. 28666342 10.1093/heapol/czx012

[czab011-B27] Du Châtelet A , Zamboni K, Fornah F et al 2019. Barriers and enablers to the implementation of maternal death reviews to improve quality of care in Sierra Leone. *draft paper*.

[czab011-B28] Dumont A , Fournier P, Abrahamowicz M et al, QUARITE research group. 2013. Quality of care, risk management, and technology in obstetrics to reduce hospital-based maternal mortality in Senegal and Mali (QUARITE): a cluster-randomised trial. The Lancet 382: 146–57. 10.1016/S0140-6736(13)60593-0 23721752

[czab011-B29] Dumont A , Tourigny C, Fournier P. 2009. Improving obstetric care in low-resource settings: implementation of facility-based maternal death reviews in five pilot hospitals in Senegal. Human Resources for Health 7: 61. 19627605 10.1186/1478-4491-7-61 PMC2728704

[czab011-B30] E4A 2012. *Maternal Death Surveillance and Response Systems: Overcoming Legal Challenges and Creating an Enabling Environment.* MDSR Action Network. http://mdsr-action.net/wp-content/uploads/2015/08/E4A_2012_FIGO-legal-briefing.pdf, accessed 26 February 2021.

[czab011-B31] Ethiopia Federal Ministry of Health, WHO & E4A 2016. National Report on MDSR Data from 2006-2007 EFY. Addis Ababa: Ethiopian Public Health Institute.

[czab011-B32] Every Woman Every Child 2015. The Global Strategy for Women's, Children's and Adolescents' Health. New York, NY: Every Woman Every Child.

[czab011-B33] E4A 2017. *MDSR Action Network*. Evidence 4 Action. http://mdsr-action.net/, accessed 5 September 2019.

[czab011-B34] George A , LeFevre AE, Jacobs T et al 2019. Lenses and levels: the why, what and how of measuring health system drivers of women’s, children’s and adolescents’ health with a governance focus. BMJ Global Health 4: e001316. 10.1136/bmjgh-2018-001316 PMC6590975 31297255

[czab011-B35] Gilson L. 2016. Everyday politics and the leadership of health policy implementation. Health Systems & Reform 2: 187–93. 31514600 10.1080/23288604.2016.1217367

[czab011-B36] Grellier R , Shome P. 2011. *FIGO Saving* *Mothers* *and* *Newborn Project: Summary Evaluation*. London: Options.

[czab011-B37] Hadush A , Ibro A, Abebe B et al 2016. Global experience with Maternal Death Surveillance and Response: building for the long-term. E4A Ethiopia implemented by WHO, the University of Aberdeen and Options with funding from the UK Department for International Development and the Bill and Melinda Gates Foundation.

[czab011-B38] Hofman JJ , Mohammed H. 2014. Experiences with facility-based maternal death reviews in northern Nigeria. International Journal of Gynecology & Obstetrics 126: 111–4. 24834852 10.1016/j.ijgo.2014.02.014

[czab011-B39] Hounton S , De Bernis L, Hussein J et al 2013. Towards elimination of maternal deaths: maternal deaths surveillance and response. Reproductive Health 10: 1. 23279882 10.1186/1742-4755-10-1 PMC3562216

[czab011-B40] Hulscher ME , Schouten LM, Grol RP et al 2013. Determinants of success of quality improvement collaboratives: what does the literature show? BMJ Quality & Safety 22: 19–31. 10.1136/bmjqs-2011-000651 22879447

[czab011-B41] Hussein J , Hirose A, Owolabi O et al 2016. Maternal death and obstetric care audits in Nigeria: a systematic review of barriers and enabling factors in the provision of emergency care. Reproductive Health 13: 47. 27102983 10.1186/s12978-016-0158-4 PMC4840864

[czab011-B42] Independent Expert Review Group 2014. A Post-2015 Vision. The Third Report of the Independent Expert Review Group on Information and Accountability for Women’s and Children’s Health. Geneva: World Health Organization.

[czab011-B43] Ivers N , Jamtvedt G, Flottorp S et al 2012. Audit and feedback: effects on professional practice and healthcare outcomes. Cochrane Database of Systematic Review 6: CD000259. 10.1002/14651858.CD000259.pub3 PMC11338587 22696318

[czab011-B44] Johnston G , Crombie IK, Davies HT et al 2000. Reviewing audit: barriers and facilitating factors for effective clinical audit. Quality in Health Care 9: 23–36. 10848367 10.1136/qhc.9.1.23 PMC1743496

[czab011-B45] Karamagi E , Sensalire S, Chakura A et al 2018. Maternal Perinatal Death Surveillance Review (MPDSR): strengthening reviews to save more lives in Uganda. Uganda: USAID ASSIST Project.

[czab011-B46] Kerber KJ , Mathai M, Lewis G et al 2015. Counting every stillbirth and neonatal death through mortality audit to improve quality of care for every pregnant woman and her baby. BMC Pregnancy Childbirth 15 Suppl 2: S9. 26391558 10.1186/1471-2393-15-S2-S9 PMC4577789

[czab011-B47] Khatri N , Brown GD, Hicks LL. 2009. From a blame culture to a just culture in health care. Health Care Management Review 34: 312–22. 19858916 10.1097/HMR.0b013e3181a3b709

[czab011-B48] Kinney MV , Walugembe DR, Wanduru P et al 2019. Implementation of maternal and perinatal death reviews: a scoping review protocol. BMJ Open 9: e031328. 10.1136/bmjopen-2019-031328 PMC6886965 31780590

[czab011-B49] Koblinsky M. 2017. Maternal death surveillance and response: a tall order for effectiveness in resource-poor settings. Global Health: Science and Practice 5: 333–7. 10.9745/GHSP-D-17-00308 PMC5620330 28963168

[czab011-B50] Koblinsky M , Kaptiningsih A. Fitriyani 2017. Indonesia: reducing maternal & perinatal deaths through MPDSR – mapping the possibilities. Draft report for USAID prepared by Management Systems International.

[czab011-B51] Kongnyuy E , van den Broek N. 2009. Audit for maternal and newborn health services in resource-poor countries. BJOG: An International Journal of Obstetrics & Gynaecology 116: 7–10. 10.1111/j.1471-0528.2008.01994.x 19087075

[czab011-B52] Kruk ME , Marchant T, Doubova S et al 2017. The Lancet Global Health Commission on High Quality Health Systems-where's the complexity? - authors' reply. The Lancet Global Health 5: e572. 28495258 10.1016/S2214-109X(17)30178-X

[czab011-B53] Levac D , Colquhoun H, O'Brien KK. 2010. Scoping studies: advancing the methodology. Implement Sci 5: 69. 20854677 10.1186/1748-5908-5-69 PMC2954944

[czab011-B54] Lewis G. 2014a. The cultural environment behind successful maternal death and morbidity reviews. BJOG 121 Suppl 4: 24–31. 25236630 10.1111/1471-0528.12801

[czab011-B55] Lewis G. 2014b. Emerging lessons from the FIGO LOGIC initiative on maternal death and near-miss reviews. Int J Gynaecol Obstet 127 Suppl 1: S17–20. 25128930 10.1016/j.ijgo.2014.07.007

[czab011-B56] Lusambili A , Jepkosgei J, Nzinga J et al 2019. What do we know about maternal and perinatal mortality and morbidity audits in sub-Saharan Africa? A scoping literature review. International Journal of Human Rights in Healthcare 12: 192–207.

[czab011-B57] Magoma M , Massinde A, Majinge C et al 2015. Maternal death reviews at Bugando hospital north-western Tanzania: a 2008-2012 retrospective analysis. BMC Pregnancy and Childbirth 15: 333. 26670664 10.1186/s12884-015-0781-z PMC4681083

[czab011-B58] Martin H , Blake A, Bohle C et al 2016. Strengthening accountability for improved maternal and newborn health: a mapping of studies in Sub-Saharan Africa. International Journal of Gynecology & Obstetrics 135: 345–57. 27802869 10.1016/j.ijgo.2016.09.008

[czab011-B59] Mathole T , Lembani M, Jackson D et al 2018. Leadership and the functioning of maternal health services in two rural district hospitals in South Africa. Health Policy and Planning 33: ii5–15. 30053038 10.1093/heapol/czx174 PMC6037108

[czab011-B60] McSherry R , Pearce P. 2011. Clinical Governance: A Guide to Implementation for Health Care Professionals*.* Hoboken, NJ: Wiley.

[czab011-B61] MCSP 2017a. Assessment of Maternal and Perinatal Death Surveillance and Response Implementation in Nigeria. Washington, DC: Maternal Child Survival Program.

[czab011-B62] MCSP 2017b. Assessment of Maternal and Perinatal Death Surveillance and Response Implementation in Rwanda. Washington, DC: Maternal Child Survival Program.

[czab011-B63] MCSP 2017c. Assessment of Maternal and Perinatal Death Surveillance and Response Implementation in Zimbabwe. Washington, DC: Maternal Child Survival Program.

[czab011-B64] MCSP 2018. Assessment of Maternal and Perinatal Death Surveillance and Response (MPDSR) Implementation in Kagera and Mara Region, Tanzania. Washington, DC: Maternal Child Survival Program.

[czab011-B65] Melberg A , Mirkuzie AH, Sisay TA et al 2019. Maternal deaths should simply be 0': politicization of maternal death reporting and review processes in Ethiopia. Health Policy and Planning 34: 492–8. 31365076 10.1093/heapol/czz075 PMC6788214

[czab011-B66] Mensah Abrampah N , Syed SB, Hirschhorn LR et al 2018. Quality improvement and emerging global health priorities. International Journal for Quality in Health Care 30: 5–9. 29873793 10.1093/intqhc/mzy007 PMC5909628

[czab011-B67] Ministry of Health and Sanitation [Sierra Leone] 2017. Maternal Death Surveillance and Response: Annual Report 2016. Free Town, Sierra Leone: Directorate of Reproductive & Child Health, Ministry of Health and Sanitation [Sierra Leone].

[czab011-B68] Muffler N , Trabelssi MEH, De Brouwere V. 2007. Scaling up clinical audits of obstetric cases in Morocco. Tropical Medicine & International Health 12: 1248–57. 17956508 10.1111/j.1365-3156.2007.01911.x

[czab011-B69] Mukinda FK , Van Belle S, George A et al 2020a. The crowded space of local accountability for maternal, newborn and child health: a case study of the South African health system. Health Policy and Planning 35: 279–90. 31865365 10.1093/heapol/czz162 PMC7152728

[czab011-B70] Mukinda FK , Van Belle S, Schneider H. 2020b. Perceptions and experiences of frontline health managers and providers on accountability in a South African health district. International Journal for Equity in Health 19: 110. 32611355 10.1186/s12939-020-01229-w PMC7328263

[czab011-B71] Mutsigiri-Murewanhema F , Mafaune PT, Juru T et al 2017. Evaluation of the maternal mortality surveillance system in Mutare district, Zimbabwe, 2014-2015: a cross sectional study. Pan African Medical Journal 27: 204. 10.11604/pamj.2017.27.204.7210 PMC5579423 28904729

[czab011-B72] Nam S. 2011. *Final Evaluation: FIGO Saving Mothers and Newborns Project in Uganda: reduction of Maternal and Newborn Mortality in Uganda.* London: Options.

[czab011-B73] Nilsen P. 2015. Making sense of implementation theories, models and frameworks. Implementation Science 10: 53. 25895742 10.1186/s13012-015-0242-0 PMC4406164

[czab011-B74] Nyamtema AS , de Jong AB, Urassa DP et al 2011. Using audit to enhance quality of maternity care in resource limited countries: lessons learnt from rural Tanzania. BMC Pregnancy and Childbirth 11: 94. 22088168 10.1186/1471-2393-11-94 PMC3226647

[czab011-B75] O'Hagan J , Persaud D. 2009. Creating a culture of accountability in health care. The Health Care Manager 28: 124–33. 19433930 10.1097/HCM.0b013e3181a2eb2b

[czab011-B76] Pattinson R , Kerber K, Waiswa P et al 2009. Perinatal mortality audit: counting, accountability, and overcoming challenges in scaling up in low- and middle-income countries. International Journal of Gynecology & Obstetrics 107 Suppl 1: S113–21, S121–2. 19815206 10.1016/j.ijgo.2009.07.011

[czab011-B77] Pattinson RC , Say L, Makin JD et al. 2005. Critical incident audit and feedback to improve perinatal and maternal mortality and morbidity. Cochrane Database Syst Rev 4: CD002961. 16235307 10.1002/14651858.CD002961.pub2 PMC4171456

[czab011-B78] Pearson L , deBernis L, Shoo R. 2009. Maternal death review in Africa. International Journal of Gynecology & Obstetrics 106: 89–94. 19428010 10.1016/j.ijgo.2009.04.009

[czab011-B79] Persson LA. 2017. Bridging the quality chasm in maternal, newborn, and child healthcare in low- and middle-income countries. PLoS Medicine 14: e1002465. 29232389 10.1371/journal.pmed.1002465 PMC5726613

[czab011-B80] * *Peters, M. D. J., Godfrey, C., McInerney, P., Baldini Soares, C., Khalil, H. & Parker, D. 2017. Scoping Reviews.* *In: Aromataris E, Munn Z (Editors). Joanna Briggs Institute Reviewer's Manual.* The Joanna Briggs Institute. https://reviewersmanual.joannabriggs.org/, accessed 26 February 2021.*

[czab011-B81] Purandare C , Bhardwaj A, Malhotra M et al. 2014. Every death counts: electronic tracking systems for maternal death review in India. International Journal of Gynecology & Obstetrics 127 Suppl 1: S35–9. 25262442 10.1016/j.ijgo.2014.09.003

[czab011-B82] Raven J , Hofman J, Adegoke A et al. 2011. Methodology and tools for quality improvement in maternal and newborn health care. International Journal of Gynecology & Obstetrics 114: 4–9. 21621681 10.1016/j.ijgo.2011.02.007

[czab011-B83] Rhoda NR , Greenfield D, Muller M et al 2014. Experiences with perinatal death reviews in South Africa–the Perinatal Problem Identification Programme: scaling up from programme to province to country. BJOG: An International Journal of Obstetrics and Gynaecology 121 Suppl 4: 160–6. 25236651 10.1111/1471-0528.12997

[czab011-B84] Richard F , Ouedraogo C, Zongo V et al 2009. The difficulty of questioning clinical practice: experience of facility-based case reviews in Ouagadougou, Burkina Faso. BJOG: An International Journal of Obstetrics & Gynaecology 116: 38–44. 18503575 10.1111/j.1471-0528.2008.01741.x

[czab011-B85] Schneider H , George A, Mukinda F et al. 2020. District governance and improved maternal, neonatal and child health in South Africa: pathways of change. Health Systems & Reform 6: e1669943. 32040355 10.1080/23288604.2019.1669943

[czab011-B86] Scott H , Danel I. 2016. Accountability for improving maternal and newborn health. Best Practice & Research Clinical Obstetrics & Gynaecology 36: 45–56. 27473405 10.1016/j.bpobgyn.2016.05.009

[czab011-B87] Sheikh K , Gilson L, Agyepong IA et al 2011. Building the field of health policy and systems research: framing the questions. PLoS Medicine 8: e1001073. 21857809 10.1371/journal.pmed.1001073 PMC3156683

[czab011-B88] Smith H , Ameh C, Godia P et al 2017a. Authors' response to editorial: maternal death surveillance and response: a tall order for effectiveness in resource-poor settings. Global Health: Science and Practice 5: 697–8. 10.9745/GHSP-D-17-00407 PMC5752615 29284703

[czab011-B89] Smith H , Ameh C, Godia P et al 2017b. Implementing maternal death surveillance and response in Kenya: incremental progress and lessons learned. Global Health: Science and Practice 5: 345–54. 10.9745/GHSP-D-17-00130 PMC5620333 28963171

[czab011-B90] Smith H , Ameh C, Roos N et al. 2017c. Implementing maternal death surveillance and response: a review of lessons from country case studies. BMC Pregnancy and Childbirth 17: 233. 28716124 10.1186/s12884-017-1405-6 PMC5513145

[czab011-B91] Spicer N , Hamza YA, Berhanu D et al 2018. ‘ The development sector is a graveyard of pilot projects!’ Six critical actions for externally funded implementers to foster scale-up of maternal and newborn health innovations in low and middle-income countries. Globalization and Health 14: 74. 30053858 10.1186/s12992-018-0389-y PMC6063024

[czab011-B92] Tapesana S , Chirundu D, Juru T et al 2017. Evaluation of the maternal death surveillance and response system, Sanyati, Zimbabwe 2017. Texila International Journal of Public Health 5: 1–10.

[czab011-B93] Topp SM. 2017. The Lancet Global Health Commission on High Quality Health Systems-where's the complexity? The Lancet Global Health 5: e571. 28495257 10.1016/S2214-109X(17)30176-6

[czab011-B94] Tricco AC , Lillie E, Zarin W et al 2018. PRISMA Extension for Scoping Reviews (PRISMA-ScR): checklist and explanation. Annals of Internal Medicine 169: 467–73. 30178033 10.7326/M18-0850

[czab011-B95] Vaismoradi M , Turunen H, Bondas T. 2013. Content analysis and thematic analysis: implications for conducting a qualitative descriptive study. Nursing & Health Sciences 15: 398–405. 23480423 10.1111/nhs.12048

[czab011-B96] van Hamersveld KT , den Bakker E, Nyamtema AS et al 2012. Barriers to conducting effective obstetric audit in Ifakara: a qualitative assessment in an under-resourced setting in Tanzania. Tropical Medicine & International Health 17: 652–7. 22469464 10.1111/j.1365-3156.2012.02972.x

[czab011-B97] WHO 2004. Beyond the Numbers: Reviewing Maternal Deaths and Complications to Make Pregnancy Safer. Geneva, World Health Organization. 10.1093/bmb/ldg009 14711752

[czab011-B98] WHO 2011. Summary Report on the Consultative Meeting on Strengthening Maternal, Perinatal and Neonatal Health Surveillance Systems, Beirut, Lebanon, 28–30 October 2010. Beirut: World Health Organization Regional Office for the Eastern Mediterranean.

[czab011-B99] WHO 2013a. Maternal Death Surveillance and Response: Technical Guidance. Geneva: World Health Organization.

[czab011-B100] WHO 2013b. Summary Report on the Regional Meeting on Maternal Death Surveillance and Response, Rabat, Morocco, 7–9 October 2013. Rabat: World Health Organization Regional Office for the Eastern Mediterranean.

[czab011-B101] WHO 2014a. Case Study Nepal: Study on the Implementation of Maternal Death Review in Five Countries in the South-East Asia Region of the World Health Organization. New Dehli: World Health Organization for South-East Asia.

[czab011-B102] WHO 2014b. Case Study Sri Lanka: Study on the Implementation of Maternal Death Review in Five Countries in the South-East Asia Region of the World Health Organization. New Dehli: World Health Organization for South-East Asia.

[czab011-B103] WHO 2014c. Study on the Implementation of Maternal Death Review in Five Countries in the South-East Asia Region of the World Health Organization. New Dehli: World Health Organization for South-East Asia.

[czab011-B104] WHO 2016a. Making Every Baby Count: Audit and Review of Stillbirths and Neonatal Deaths. Geneva: World Health Organization.

[czab011-B105] WHO 2016b. Strengthening Country Capacity on Maternal and Perinatal Death Surveillance and Response: Report of a South-East Asia Regional Meeting, 16-18 February 2016. Maldives: World Health Organization Regional Office for South-East Asia.

[czab011-B106] WHO 2016c. Time to Respond: A Report on the Global Implementation of Maternal Death Surveillance and Response (MDSR). Geneva: World Health Organization.

[czab011-B107] WHO. 2017. *Global Monitoring of Implementation of Maternal Death Surveillance and Response (MDSR)*. http://www.who.int/maternal_child_adolescent/epidemiology/maternal-death-surveillance/global-monitoring/en/, accessed 15 November 2019.

[czab011-B108] Zamboni K , Schellenberg J, Hanson C et al 2019. Assessing scalability of an intervention: why, how and who? Health Policy and Planning 34: 544–52. 31365066 10.1093/heapol/czz068 PMC6788216

