# Supplementary material for: Maternal and perinatal death surveillance and response in low- and middle-income countries: a scoping review of implementation factors
Source: Health Policy Plan. 2021 Mar 13;36(6):955–73. doi: 10.1093/heapol/czab011 (PMC8227470; doi:10.1093/heapol/czab011)
Supplement: czab011_Supp [file czab011_supp.zip › Supplementary 1.docx]

**Supplementary 1: Maternal and Perinatal Death Surveillance and Response Terminology**

MPDSR is a systematic process used to understand the medical causes and the modifiable factors that contribute to maternal and perinatal deaths to identify actions to prevent future deaths. It is a complex intervention process with multiple steps, involving multiple actors, and engagement at multiple levels of the health system. The intervention has also evolved over time, resulting in different approaches and understandings of the intervention. The aim of this supplementary file is to provide a brief history of MPDSR, an explanation for how it was conceptualized in this scoping review, as well as key terms and definitions related to MPDSR in order to clarify the authors’ perspectives around the intervention.

## Brief summary of MPDSR evolution

The intervention “MPDSR” has evolved in the past two decades resulting in the expansion of the definitions and scope of the intervention (Figure S1.1). Clinical audits, including those relating to maternal or perinatal deaths, are well-known and, in some settings, a well-established clinical practice. Clinical audits specific to maternal and/or perinatal deaths have been called maternal death reviews (MDR), perinatal death reviews, obstetric audits, facility death reviews/audits, confidential inquiries, and verbal autopsies. These different processes are similar in terms of aim but may vary in scope and approach.

In 2004, the World Health Organization (WHO) published *Beyond the numbers: reviewing maternal deaths and complications to make pregnancy safer*, a guide to the different approaches for investigating maternal deaths, notably: community death reviews or verbal autopsies, facility death reviews and confidential inquiries (WHO, 2004). Table S1.1 shows how these approaches vary in terms of level and description. The document recommended that all countries should established maternal death audit systems to help reduce maternal deaths, and many countries took forward this recommendation and develop national guidelines and implemented MDR programs.

**Table S1.1: Key methodologies used for action and improvement through the use of maternal death or severe morbidity/near-miss reviews**

| Level | Methodology | Description |
| --- | --- | --- |
| National/  regional/  district | Confidential enquiries into maternal deaths | A qualitative and quantitative systematic, anonymous, review of all or a representative sample of maternal deaths occurring at an area, regional (state) or national level and learning lessons for action and improvement. A slower response. |
| Facility | Facility-based maternal death  reviews | Learning lessons for action and improvement through an in-depth assessment of the causes and circumstances surrounding the deaths of pregnant or recently delivered mothers in health facilities. These may also include identifying community-based factors. A quick response to local issues. |
| Facility | Near-miss or sever morbidity reviews | Learning lessons for action and improvement through the assessment of cases where pregnant or recently delivered women survive severe life-threatening complications, so called ‘near-misses’. These can be less threatening than death reviews as the mothers survive. Mothers can also participate in the evaluation of their care. A quick response to local issues. |
| Community | Community death reviews, social autopsies, or verbal autopsies | Learning lessons for action and improvement through the assessment of cases where pregnant or recently delivered women die outside hospital. This includes identifying the probable medical causes for this as well as any underlying personal, family, community or other factors. A quick response at community level. |

*Source: methodology and descriptions from WHO Beyond the Numbers (WHO, 2004) and adapted by Lewis (2014)*

In 2011, the Commission on Information and Accountability (CoIA) for Women’s and Children’s Health called call for accountability of maternal health through a continuous monitor–review–act cycle, which includes national oversight, monitoring of results, multi-stakeholder reviews, and action—all ingredients of surveillance and response systems (Commission on Information and Accountability, 2011). In 2012, the United Nations Commission on the Status of Women passed a resolution calling for the elimination of preventable maternal mortality, placing additional political pressure on counting all maternal deaths. As a result, WHO convened a new a technical working group to advance the understanding and implementation of maternal death surveillance and response (MDSR). Considered as “a relatively new approach to investigating maternal deaths and taking action based on the findings,” MDSR evolved from the established system of maternal death review but put greater importance on the follow-up action (response) and the continual monitoring, or surveillance (WHO, 2020). WHO and the technical working group released the *Maternal Death Surveillance and Response: Technical Guidance, Information for Action to Prevent Maternal Death* in 2013, which provided guidance for establishing and implementing MDSR systems (World Health Organization, 2013). Expanding maternal death reviews to include surveillance, MDSR sought to identify most—if not all—maternal deaths, and uses facility and community based reviews, together with other surveillance methods, to feed a national database with a more limited analysis of causes (medical and other), circumstances, and determinants of maternal mortality (Hounton et al., 2013). The argument for expansion of MDR to MDSR was to enable greater accountability by creating a system whereby the data would be used at district and national levels with a feedback mechanism in place to address system level issues identified, such as organization of national blood banks or addressing human resources gaps. However, when it was released, some experts heeded caution that this new system may limited the ability of the maternal death reviews to enable local improvement of quality of care and feared confidentiality might be threatened (De Brouwere et al., 2013). Mortality audit for maternal mortality became more widespread with over two-thirds of countries globally reporting to have in place, fully or partially, MDSR with significant investments by countries into their audit systems by 2015 (iERG, 2015).

Around this time, there was also rising attention to end preventable newborn deaths and stillbirths (WHO, 2014, iERG, 2015, de Bernis et al., 2016). As a result, the WHO released *Making every baby count: audit and review of stillbirths and neonatal deaths* in 2016 (WHO, 2016). This guideline provided support for conducting mortality audits for stillbirths and neonatal deaths (i.e. identifying cases, collecting information, analysing the data collected to recommend solutions to improve the quality of care, and implementing the changes within a continuous evaluation and response cycle) as well as tools for adaptation at national, sub-national or facility level. The guidelines was released at the same time as the *WHO application of ICD-10 to deaths during the perinatal period: ICD-PM (ICD-perinatal mortality)* (WHO, 2016 ), which provided a new system for classifying causes of death that linked stillbirths and neonatal deaths to contributing maternal conditions, where applicable, in a way that is consistent across all settings.

In 2017, WHO expanded the MDSR Working Group to include perinatal in order to harmonize activities, becoming the “MPDSR Technical Working Group”. WHO MPDSR operational guidelines are currently in development; however, other partners have release operational guidelines for MDSR and perinatal death reviews (UNICEF, 2018, MCSP, 2020).

**Figure S1.1: WHO visual showing transition to MDSR**
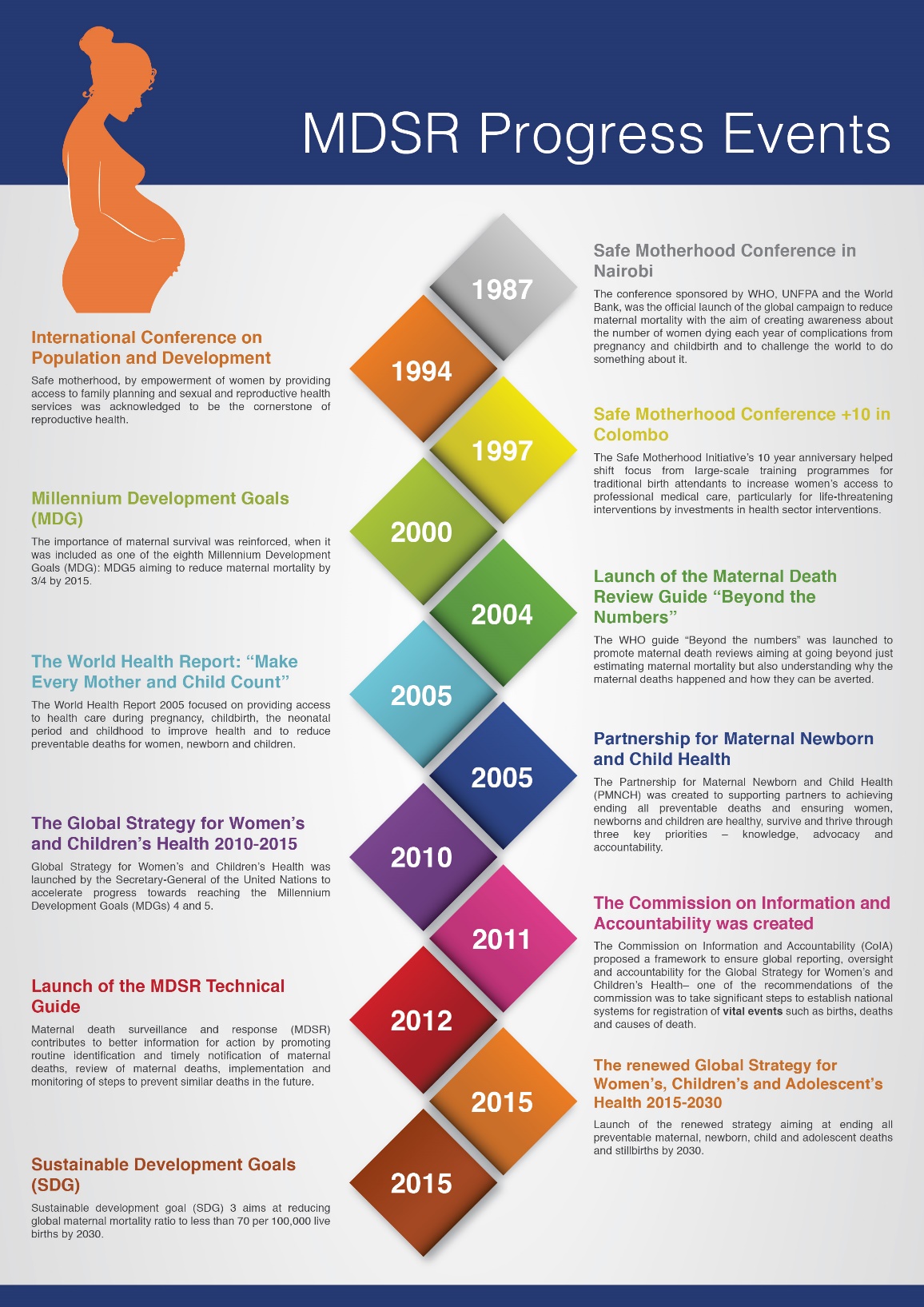


Source: Figure from WHO Webpage (accessed 11 December 2020)

<https://www.who.int/maternal_child_adolescent/epidemiology/maternal-death-surveillance/mdsr-timeline.jpg?ua=1>

## Defining MPDSR

Since there is no official WHO definition of MPDSR, for the purposes of this review, we defined MPDSR as “a continuous cycle of identification, notification and review of maternal and/or perinatal deaths followed by actions to address identified contributing factors and to prevent future deaths based on acting on gaps identified in the audit.” The protocol paper for this review conceptualizes M/PDSR as the following (Kinney et al., 2019):

*M/PDSR is a continuous action cycle for quality improvement that links maternal and perinatal mortality data from the local to the national level. M/PDSR can be considered as an intervention as well as an implementation process since it is a set of efforts geared towards facilitating change (Bauer et al., 2015). At all levels, the process relies on the effective reporting and assigning causes to deaths, on identifying actions that may contribute to the prevention of further deaths, assigning those actions to particular groups or individuals within a specified timeframe, and following up to ensure that those actions have been implemented. At the facility level, a six-step cycle of auditing deaths is recommended whereby: 1) cases for review are identified; 2) information on these cases is collected; 3) the information is analyzed and discussed by the MPDSR committee; 4) solutions are recommended based on the findings of the analysis; 5) solutions are implemented; and 6) feedback or reflection on if solutions were implemented and what worked or didn’t in order to inform the process moving forward (WHO, 2016).*

*In a well-functioning health system, the information from the facility-level audits feeds up into a sub-national level process whereby information about maternal and perinatal deaths is received, compiled, reviewed for completeness and any relevant actions at that level or above. The information is further analyzed and then disseminated to appropriate stakeholders, including other sub-national entities who would have their own processes (e.g. District to Province). Information from the sub-national level is compiled and sent to national level whereby further synthesis and analyses are conducted. This often leads to a national annual report that is then disseminated back to subnational and facility levels.*

*As a concept, M/PDSR functions at multiple levels of the health system – national, subnational, facility (and for some countries community level components are included in the process). The communication system and inter-connectedness between the different levels is an important component of M/PDSR since the process is a reporting mechanism moving continuously from bottom up – facility to national – and also from top down – national to facility. For example, recommendations to the national Ministry of Health could be identified during a facility-level audit process. This information should be fed up through the system to reach the national level decision makers. Likewise, the national level needs information from the facility-level and sub-national level in order to assess the situation of maternal and perinatal mortality in the country in order to make recommendations at sub-national and facility levels.*

In terms of types of MDRs, this review only included studies that focused on facility-death reviews (see categories in Table 1). Confidential inquiries are less common in LMIC settings, where maternal mortality remains high. As facility births have increased substantially in recent decades, most deaths occur at facility level. While social and verbal autopsies are important processes in some settings, they were excluded from this review.

## Other terms and definitions

**Mortality and morbidity audits:** a means for improving the quality of patient care and outcomes by systematic review of clinical management and comparing outcomes against criteria or accepted standards of care. This allows identification of gaps in quality of care and changes to be made. The objective is to determine whether patient care is consistent with best practices (WHO, 2018). “Mortality audit” is a well-known and well-established clinical practice (Pattinson et al., 2005), while “death review” is a term used in the maternal death surveillance and response guidance. Therefore, these terms are often used interchangeably (WHO, 2016).

**Maternal Death Surveillance and Response (MDSR)**: a form of continuous surveillance linking the health information system and quality improvement processes from local to national levels. It includes the routine identification, notification, quantification, and determination of causes and avoidability of all maternal deaths, as well as the use of this information to respond with actions that will prevent future deaths. Elimination of preventable maternal mortality is the goal of MDSR (WHO, 2013).

**Mortality audit (perinatal)**: A mortality audit is the process of capturing information on the number and causes of stillbirths and neonatal deaths, and then identifying specific cases for systematic, critical analysis of the quality of care received, in a no-blame, interdisciplinary setting, with a view to improving the care provided to all mothers and babies. It is an established mechanism to examine the circumstances surrounding each death including any breakdowns in care that may have been preventable. Applying the audit cycle to the circumstances surrounding deaths is an established quality improvement strategy that can highlight breakdowns in clinical care at the local level as well as breakdowns in processes at the district or national level, and ultimately improve the civil registration and vital statistics (CRVS) system and quality of care overall (WHO, 2016).

**Maternal death**: The death of a woman while pregnant or within 42 days of the termination of pregnancy irrespective of the duration and site of the pregnancy, from any cause related to or aggravated by the pregnancy or its management but not from accidental or incidental causes. Can be direct (resulting from obstetric complications of the pregnancy state) or indirect (resulting from previously existing disease or disease that developed during pregnancy).

**Stillbirth***: A death that occurs before birth in a baby weighing ≥1000g or, if missing, ≥28 completed weeks of gestation or, if missing, body length ≥35cm (this definition is used for international comparison; ICD-10 uses birthweight ≥500g or, if missing, ≥22 completed weeks or, if missing, body length ≥25cm).

**Neonatal death**: A death which occurs in the first 28 days of life.

**Perinatal death**: A foetal death of a foetus born weighing ≥ 1000 g and/or after 28 completed weeks of gestation, plus neonatal deaths through the first 7 completed days after birth.

**Modifiable factor**: Circumstances that may have prevented a death if a different course of action was taken (missed opportunity). Using “modifiable” instead of “avoidable” or “substandard” helps limit opportunities for blame and presents potential for positive change. Approaches for classifying modifiable factors range from simple to more analytical and complex.

S*ources for unreferenced definitions: World Health Organization. 2016*

**The definition for developed countries includes foetuses born weighing ≥ 500 g and/or after 22 completed weeks.*

**REFERENCES**

Bauer, M. S., Damschroder, L., Hagedorn, H., Smith, J. & Kilbourne, A. M. 2015. An introduction to implementation science for the non-specialist. *BMC Psychol,* 3**,** 32.

Commission on Information and Accountability 2011. *Keeping promises, measuring results: Commission on Information and Accountability for Women's and Children's Health,* Geneva, World Health Organization.

de Bernis, L., Kinney, M. V., Stones, W., Ten Hoope-Bender, P., Vivio, D., Leisher, S. H., Bhutta, Z. A., Gulmezoglu, M., Mathai, M., Belizan, J. M., Franco, L., McDougall, L., Zeitlin, J., Malata, A., Dickson, K. E., Lawn, J. E., Lancet Ending Preventable Stillbirths Series study, g. & Lancet Ending Preventable Stillbirths Series Advisory, G. 2016. Stillbirths: ending preventable deaths by 2030. *Lancet,* 387**,** 703-16.

De Brouwere, V., Lewis, G., Filippi, V., Delvaux, T., Beyeza-Kashesya, J., Gebrehiwot, Y., Bique, C. & Taylor, D. 2013. Maternal Death Reviews. *Lancet,* 381**,** 1718-9.

Hounton, S., De Bernis, L., Hussein, J., Graham, W. J., Danel, I., Byass, P. & Mason, E. M. 2013. Towards elimination of maternal deaths: maternal deaths surveillance and response. *Reprod Health,* 10**,** 1.

iERG 2015. *Every woman, every child, every adolescent: achievements and prospects: the final report of the independent Expert Review Group on Information and Accountability for Women’s and Children’s health,* Geneva, Switzerland, World Health Organization.

Kinney, M. V., Walugembe, D. R., Wanduru, P., Waiswa, P. & George, A. S. 2019. Implementation of maternal and perinatal death reviews: a scoping review protocol. *BMJ Open,* 9**,** e031328.

MCSP 2020. *Maternal and perinatal death surveillance and response (MPDSR) Capacity-Building Materials: MDSR module: Facilitator's guide. ,* Washington, DC, MCSP.

Pattinson, R. C., Say, L., Makin, J. D. & Bastos, M. H. 2005. Critical incident audit and feedback to improve perinatal and maternal mortality and morbidity. *Cochrane Database Syst Rev***,** CD002961.

UNICEF 2018. *Skill Building on Perinatal Death Reviews,* New York, UNICEF.

WHO 2004. *Beyond the numbers: Reviewing maternal deaths and complications to make pregnancy safer,* Geneva, World Health Organization.

WHO 2013. *Maternal death surveillance and response: technical guidance,* Geneva, World Health Organization.

WHO 2014. *Every Newborn: An action plan to end preventable newborn deaths,* Geneva, World Health Organization.

WHO 2016. *Making every baby count: audit and review of stillbirths and neonatal deaths,* Geneva, World Health Organization.

WHO 2016 *The WHO Application of ICD-10 to perinatal deaths: ICD-PM,* Geneva, Switzerland, World Health Organization.

WHO 2018. *Improving the quality of paediatric care: an operational guide for facility-based audit and review of paediatric mortality* Geneva, Switzerland, World Health Organization.

WHO. 2020. *Maternal Death Surveillance and Response - background* [Online]. Geneva, Switzerland: World Health Organization. Available: <https://www.who.int/maternal_child_adolescent/epidemiology/maternal-death-surveillance/background/en/> [Accessed 11 December 2020].

World Health Organization 2013. *Maternal death surveillance and response: technical guidance. Information for action to prevent maternal death,* Geneva, Switzerland, World Health Organization.
