# Supplementary material for: Maternal and perinatal death surveillance and response in low- and middle-income countries: a scoping review of implementation factors
Source: Health Policy Plan. 2021 Mar 13;36(6):955–73. doi: 10.1093/heapol/czab011 (PMC8227470; doi:10.1093/heapol/czab011)
Supplement: czab011_Supp [file czab011_supp.zip › Supplementary 2.docx]

Supplementary 2: Description of changes to the conceptual framework

The theoretical conceptual framework used for this scoping review includes four domains (intervention^[[1]](#footnote-1)^, outer setting, inner setting, and individual) with 24 constructs. Within each domain, we consider three different lenses through which to understand and measure health system drivers of women’s and children’s health (George and al. 2019 ). A service delivery lens includes the tangible inputs needed for MPDSR implementation; a societal lens includes constructs that focus on social understanding and relationships; and a systems lens includes constructs that emphasis change dynamics, which includes adaptive learning to contexts in ways that are not always anticipated.

The framework underwent some minor revisions during the data extraction and analysis process. Table 2.1 shows the modifications made and final list of constructs. The framework domains remained the same; however, we modified some constructs to better reflect the elements contributing to MPDSR in practice based on the review findings (Kinney et al. 2019). We designed a new figure to help visually capture the essence of the framework. The dark grey items are the inputs that are needed for implementation (service delivery lens); these are things that the World Health Organization is already monitoring/measuring/describing. The black shaded items are the interactions between those involved in the implementation (societal lens). The light grey shaded items are things that trigger change (systems lens). For MPDSR to function, as intended, the intervention process needs to link through the health system, fit context (adaptable), enable a learning climate and agents to push change;, and allow individuals to critically think and collaborate.

The below table shows the evolution of the constructs for each domain and how these link to the original domains and constructs of the CFIR. The first domain features the intervention characteristics and process. The complexity of MPDSR as a process intervention with multi-faceted components and steps meant that we did not think two separate domains for intervention and process were needed and thus were combined. As with most interventions, there will be some adaptability at each level of MPDSR as it is implemented in different settings and at different levels. Factors within the intervention domain for MPDSR may include the steps of the audit cycle, cost of implementation, perceived legitimacy of the process as resulting in change, and the perceived ability to test, adapt and implement it. We moved the individual domain next in the framework in order to emphasize the importance of the characteristics of the individuals involved in implementation. Factors include their individual capacity and knowledge, their motivations and commitments to the implement MPDSR, as well as their commitment to the team or organization, and their willingness towards adapting to the intervention. The final two domains, the inner and outer setting, continuously interface and influence each other; thus the line between them is porous. The outer setting includes factors external to the organization that influence implementation of MPDSR; whereas the inner setting includes factors internal to the organization. As outer setting factors influence implementation, change occurs in the inner setting. For MPDSR implementation, the outer setting factors include policy and planning, linkages to other actors (such as professional association), pressures to implement, community links, and communication channels. For the inner setting, implementation factors include readiness to implement, the structural characteristics of the organization implementing MPDSR, the organizational culture, and the quality of communication and relationships, and engaged leaders (also called champions in some settings).

**Figure S1.1: Theoretical framework for studying MPDSR implementation**
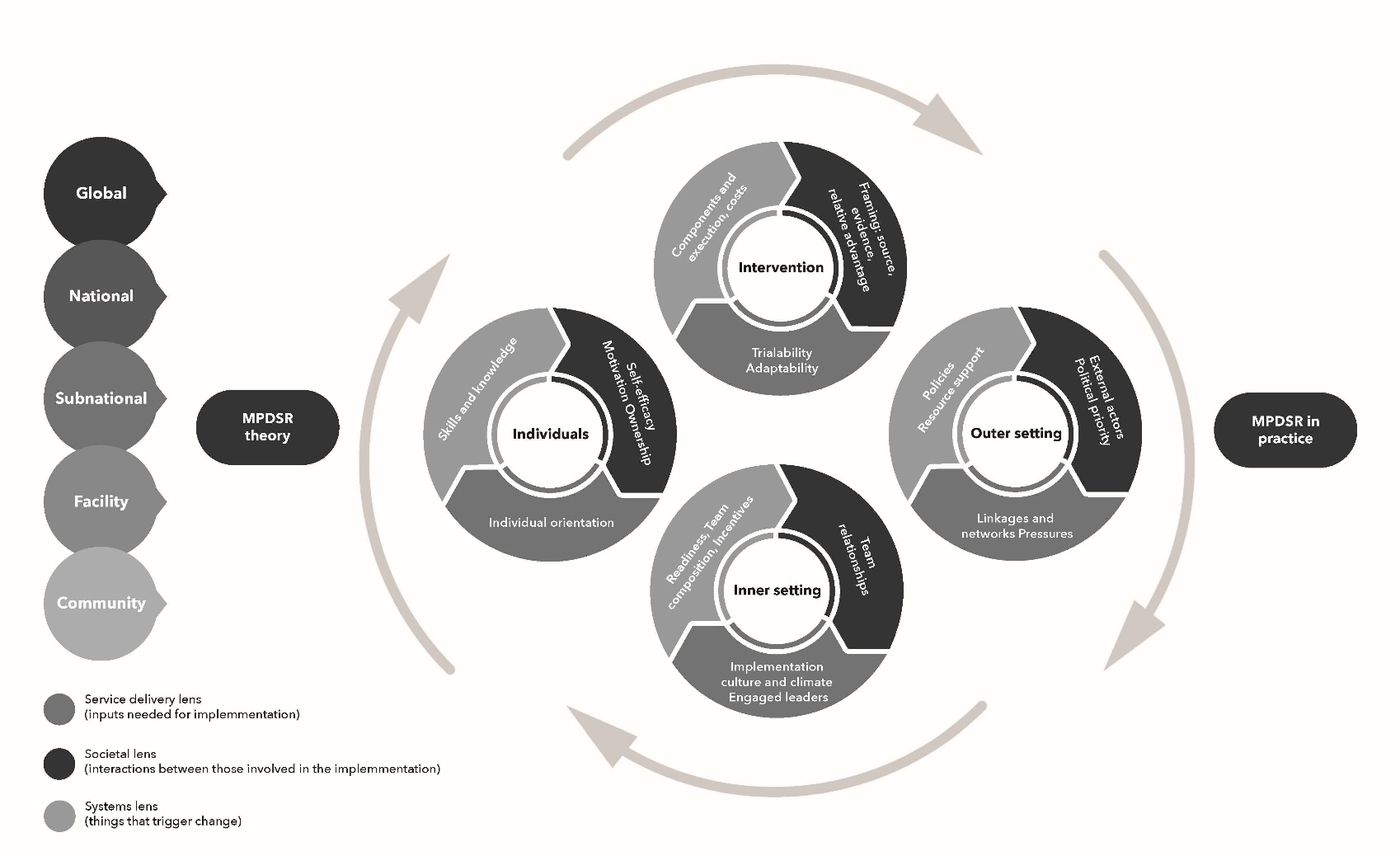


**Table S2.1: Overview of changes the theoretical implementation framework (from the original CFIR constructs to the revisions undertaken during the review process)**

| **ORIGINS OF CONSTRUCT – CFIR** | **PRESENTED IN PROTOCOL (Kinney et al. 2019)** | **REVISED FEBURARY 2020** | **REVISED MAY 2020**  ***(reason for edit in italics)*** |
| --- | --- | --- | --- |
|  | **Domain 1: Intervention/ MPDSR** | **Domain 1: Intervention/ MPDSR** | **Domain 1: Intervention/ MPDSR** |
|  | SERVICE DELIVERY LENS (tangible inputs) | SERVICE DELIVERY LENS (tangible inputs) | SERVICE DELIVERY LENS (tangible inputs) |
| **Intervention process**  Executing: Carrying out or accomplishing the implementation according to plan. | Executing audit: steps of cycle implemented | Operationalisation of audit cycle: steps of cycle reported on by level (a descriptive analysis) | Components and execution: steps of the audit cycle described and reported on by level (a descriptive analysis)  *[changed name of construct to align better with original construct]* |
| **Intervention characteristics**  Cost: Costs of the intervention and costs associated with implementing the intervention including investment, supply, and opportunity costs. | Cost and funding for the audit process including collecting data, meeting related costs incl transport, specific training, running secretariat, time | Cost for the audit process including collecting data, meeting related costs incl transport, specific training, running secretariat, time | Cost relating to the audit process including collecting data, meeting related costs such as transport, specific training, running secretariat, time  *(Clarified definition)* |
|  | SOCIETAL LENS (social understanding and relationships) | SOCIETAL LENS (social understanding and relationships) |  |
| **Intervention characteristics**  Intervention Source: Perception of key stakeholders about whether the intervention is externally or internally developed. | Intervention source: Legitimacy depending on whether intervention is externally or internally developed | Framing - Intervention source: ownership of implementation guideline and stakeholder perceptions on whether the intervention is externally or internally developed | Framing - Intervention source: ownership of implementation guideline and stakeholder perceptions on whether the intervention is externally or internally developed  *No change* |
| **Intervention characteristics**  Evidence Strength & Quality: Stakeholders’ perceptions of the quality and validity of evidence supporting the belief that the intervention will have desired outcomes.investment, supply, and opportunity costs. | Evidence strength & quality: Evidence supporting the belief that the intervention will have desired outcomes (reduced mortality; changes undertaken to improve quality of care / "response") | Framing - Evidence strength & quality: Evidence supporting the belief that the intervention will have desired outcomes (reduced mortality; changes undertaken to improve quality of care / "response") | Framing - Evidence strength & quality: Evidence supporting the belief that the intervention will have desired outcomes (reduced mortality; changes undertaken to improve quality of care / "response")  *No change* |
| **Intervention characteristics**  Relative Advantage: Stakeholders’ perception of the advantage of implementing the intervention versus an alternative solution. | Relative advantage: Perception of the advantage of implementing the intervention versus an alternative solution | Framing - Relative advantage: Perception of the advantage of implementing the intervention versus an alternative solution | Framing - Relative advantage: Perception of the advantage of implementing the intervention versus an alternative solution  *No change* |
|  | SYSTEMS LENS (change dynamics) | SYSTEMS LENS (change dynamics) |  |
| **Intervention characteristics**  Trialability The ability to test the intervention on a small scale in the organization, and to be able to reverse course (undo implementation) if warranted. | Trialability: Ability to test/ pilot the intervention on a small scale, learn and revise if warranted | Trialability: Ability to test/ pilot the intervention on a small scale, learn and revise if warranted | Trialability: Ability to test/ pilot the intervention on a small scale, learn and revise if warranted  *No change* |
| **Intervention characteristics**  Design Quality & Packaging: Perceived excellence in how the intervention is bundled, presented, and assembled. | Reflectivity: feedback about the progress and quality of implementation accompanied with regular personal and team debriefing about progress and experience. | *Decided to remove because concepts overlapped with other constructs* |  |
| **Intervention characteristics**  Adaptability: The degree to which an intervention can be adapted, tailored, refined, or reinvented to meet local needs. | Adaptability: Degree to which an intervention can be tailored to meet the needs of an organization (core vs. peripheral elements) | Adaptability: Degree to which an intervention can be tailored to meet the needs of an organization (core vs. peripheral elements) | Adaptability: Degree to which an intervention can be tailored to meet the needs of an organization (core vs. peripheral elements)  *No change* |
| **Intervention characteristics**  Complexity: Perceived difficulty of implementation, reflected by duration, scope, radicalness, disruptiveness, centrality, and intricacy and number of steps required to implement. | Complexity: Perceived difficulty of implementation **by the implementers** (extent of disruption, number of elements/steps, extent of discretion, health system levels, actors) | *Decided to remove because concepts overlapped with other constructs* |  |
|  | **Domain 2:** **Outer Setting/ Broader context** |  | **Domain 4:** **Outer Setting/ Broader context** |
|  | SERVICE DELIVERY LENS (tangible inputs) | SERVICE DELIVERY LENS (tangible inputs) |  |
| **Outer setting**  External Policy & Incentives: A broad construct that includes external strategies to spread interventions, including policy and regulations (governmental or other central entity), external mandates, recommendations and guidelines, pay-for-performance, collaboratives, and public or benchmark reporting. | Policy and planning: MPDSR policy and guidelines, Death notification requirements (legal framework for notifying deaths), Legal mandate, litigation/legal protection | Policy and planning: MPDSR policy and guidelines, Death notification requirements (legal framework for notifying deaths), Legal mandate, litigation/legal protection | Policy and planning: MPDSR policy and guidelines, Death notification requirements (legal framework for notifying deaths), Legal mandate, litigation/legal protection  *No change* |
| **Inner setting**  Available Resources: The level of resources dedicated for implementation and on-going operations, including money, training, education, physical space, and time. | Resource flows: any mention of funding support or resources for MPDSR (e.g. sponsors, related costs being funded/budgeted) | Resource support: any mention of funding support or resources for MPDSR (e.g. sponsors, related costs being funded/budgeted) | Resource support: funding or resource support for MPDSR (e.g. sponsors, budgets)  *(Clarified definition)* |
|  | SOCIETAL LENS (social understanding and relationships) | SOCIETAL LENS (social understanding and relationships) |  |
| **Intervention process**  Engaging – sub construct External Change Agents: Individuals who are affiliated with an outside entity who formally influence or facilitate intervention decisions in a desirable direction. | Linkages to other actors: Local party, Union affiliations, Professional associations, Community organisations | External actors: The role of external actors on the process (e.g. Local party, Union affiliations, Professional associations, Community organisations) as well as community or CHW engagement and participation in MPDSR | External actors: The role of external actors on the process (e.g. Local party, Union affiliations, Professional associations, Community organisations) as well as community or CHW engagement and participation in MPDSR  *No change* |
| **Outer setting**  Peer Pressure: Mimetic or competitive pressure to implement an intervention; typically because most or other key peer or competing organizations have already implemented or are in a bid for a competitive edge. | Pressure: to implement from actors and other implementers | [moved to systems lens] |  |
| **N/A** | Community links: Awareness of MPDSR in the community (grassroots); community or CHW engagement and participation in MPDSR | [removed and incorporated into external actors] |  |
|  |  |  | Political prioritization: national mobilization and awareness of issue  *(We had initially included this construct in original draft version developed before protocol version. Given the results do identify political pressure as a factor – and it was brought up on the TWG discussion as an important factor – we suggest including construct to separate out. )* |
|  | SYSTEMS LENS (change dynamics) |  |  |
| **Outer setting**  Cosmopolitanism: The degree to which an organization is networked with other external organizations. | Cosmopolitanism: Level of connectedness and networks with other health system levels, organizations and therefore openness or resistance to change | Linkages and networks between levels: Level of connectedness and networks with other health system levels, organizations and therefore openness or resistance to change | Linkages and networks between levels: Level of connectedness and networks with other health system levels, organizations and therefore openness or resistance to change  *No change* |
| **Outer setting**  Peer Pressure: Mimetic or competitive pressure to implement an intervention; typically because most or other key peer or competing organizations have already implemented or are in a bid for a competitive edge. |  | Pressure: to implement from actors and other implementers | Pressure: to implement from actors and other implementers  *No change* |
|  | **Domain 3: Inner Setting** |  |  |
|  | SERVICE DELIVERY LENS (tangible inputs) | SERVICE DELIVERY LENS (tangible inputs) |  |
| **Inner setting**  Readiness for Implementation: Tangible and immediate indicators of organizational commitment to its decision to implement an intervention.  Includes   - leadership engagement (Commitment, involvement, and accountability of leaders and managers with the implementation) - available resources (see above)   access to knowledge and information (Ease of access to digestible information and knowledge about the intervention and how to incorporate it into work tasks.) | Readiness to implement: committees formed, training, focal point identified, availability of tools, leadership engagement and management capacity, HRH workload, access to resources | Inputs to implement: committees formed, training, focal point identified, leadership informed, availability of tools, HRH workload | Readiness for Implementation: Tangible and immediate indicators of organizational commitment to its decision to implement an intervention including committees formed, training programmes, focal point identified, leadership informed, availability of tools, HRH workload  *[suggest renaming to align with CFIR and add in details]* |
| **Inner setting**  Structural Characteristics: The social architecture, age, maturity, and size of an organization. | Structural characteristics of social architecture (characteristics of the team e.g. size, interdisciplinary nature, membership regulation) | Team Composition/Structural characteristics of social architecture: characteristics of the team e.g. size, interdisciplinary nature, membership regulation | Team composition and characteristics including who comprises the team e.g. size, interdisciplinary nature, membership regulation  *(Change to name and definition to clarify meaning0* |
| **Inner setting**  Organizational Incentives & Rewards: Extrinsic incentives such as goal-sharing awards, performance reviews, promotions, and raises in salary, and less tangible incentives such as increased stature or respect. | Incentives/rewards (Disincentives/sanctions): Extrinsic incentives such as goal-sharing awards, performance reviews/promotions, training, tea or the consequences | Incentives/rewards (Disincentives/sanctions): Organizational incentives such as goal-sharing awards, performance reviews/promotions, training, tea or the consequences | Organizational incentives & rewards (or disincentives/sanctions)  such as goal-sharing awards, performance reviews/promotions, training, tea or the consequences  *(changed name to align with CFIR)* |
|  | SOCIETAL LENS (social understanding and relationships) |  |  |
| **Inner setting**  Networks & Communications: The nature and quality of webs of social networks and the nature and quality of formal and informal communications within an organization. | Networks & communication: nature and quality of communication within audit team (including hierachries, mentorship, teamwork) | Team relationship: nature and quality of communication within audit team (including hierachries, mentorship, teamwork) | Team relationship: nature and quality of communication within audit team (including hierachries, mentorship, teamwork, and management)  *No change* |
| **Inner setting**  Culture Norms, values, and basic assumptions of a given organization. | Culture: Norms and values, organizational assumptions (blame culture vs. trust) | Culture: Norms and values, organizational assumptions (blame culture vs. trust) | *Removed and incorporated with implementation climate* |
|  | SYSTEMS LENS (change dynamics) |  |  |
| **Inner setting**  Implementation Climate: The absorptive capacity for change, shared receptivity of involved individuals to an intervention, and the extent to which use of that intervention will be rewarded, supported, and expected within their organization.  Includes:   - Tension for change - Compatibility - Relative priority - Org incentives and rewards - Goals and feedback   Learning climate | Implementation climate: explanation of environment e.g. learning climate, relative priority, if there are things mentioned that are tensions/triggers for change | Implementation climate: explanation of environment e.g. learning climate, relative priority, if there are things mentioned that are tensions/triggers for change | Implementation culture and climate: explanation of environment including organizational culture, learning climate, if there are things mentioned that are tensions/triggers for change  [Based on CFIR paper concepts (see quote), these are linked concepts and is best described together under systems lens because is described as a phenomenon that varies across teams and units and is less stable over time – meaning it can change*]* |
| **Process**  Engaging: Attracting and involving appropriate individuals in the implementation and use of the intervention through a combined strategy of social marketing, education, role modeling, training, and other similar activities.  Opinion Leaders: Individuals in an organization who have formal or informal influence on the attitudes and beliefs of their colleagues with respect to implementing the intervention.  Formally Appointed Internal Implementation Leader: Individuals from within the organization who have been formally appointed with responsibility for implementing an intervention as coordinator, project manager, team leader, or other similar role.  Champions: “Individuals who dedicate themselves to supporting, marketing, and ‘driving through’ an [implementation]” [101] (p. 182), overcoming indifference or resistance that the intervention may provoke in an organization. | Agents of change: Individuals who have formal or informal influence on the attitudes and beliefs of their colleagues with respect to implementing the intervention or on the implementation process overall | Agents of change: Individuals who have formal or informal influence on the attitudes and beliefs of their colleagues with respect to implementing the intervention or on the implementation process overall | Engaged leaders: Individuals who have formal or informal influence on the attitudes and beliefs of their colleagues with respect to implementing the intervention or on the implementation process overall, e.g. “champions” or “agents of change”  *(Changed name and definition to clarify meaning)* |
|  | **Domain 4: Individuals** | **Revised Domain 4** | **Domain 2: Individuals** |
|  | SERVICE DELIVERY LENS (tangible inputs) | SERVICE DELIVERY LENS (tangible inputs) |  |
| **Characteristics of individuals**  Knowledge & Beliefs about the Intervention: Individuals’ attitudes toward and value placed on the intervention as well as familiarity with facts, truths, and principles related to the intervention. | Technical skills & Knowledge: Individual staff knowledge and competencies | Technical skills & Knowledge: Individual staff knowledge and competencies including skills for data collection and data use | Technical skills & Knowledge: Individual staff knowledge and competencies including skills for data collection and data use  *No change* |
|  | SOCIETAL LENS (social understanding and relationships) | SOCIETAL LENS (social understanding and relationships) |  |
| **Characteristics of individuals**  Self-efficacy: Individual belief in their own capabilities to execute courses of action to achieve implementation goals. | Individual motivation, Self-efficacy: An individual’s confidence in their capabilities to execute the implementation; individuals who are motivated | Individual motivation, Self-efficacy: An individual’s confidence in their capabilities to execute the implementation; individuals who are motivated | Self-efficacy: Individual belief in their own capabilities to execute courses of action to achieve implementation goals.  *[changed back to original definitions from CFIR]* |
| **Characteristics of individuals**  Individual Identification with Organization: A broad construct related to how individuals perceive the organization, and their relationship and degree of commitment with that organization. | Individual commitment/ownership to team and organization: Individuals’ perception of their commitment to the the organization and their relationship |  | Individual motivation: A broad construct related to factors that motivate individuals to implement both extrinsic and intrinsic  *[split from self-efficacy as was confusing and revised to align with CFIR original language pulling from stage of change]* |
| **Characteristics of individuals**  Individual Stage of Change: Characterization of the phase an individual is in, as he or she progresses toward skilled, enthusiastic, and sustained use of the intervention. | Individual commitment/ownership of intervention: Individuals’ perception of their commitment to the intervention | Individual commitment/ownership: Individuals’ perception of their commitment to the organization and their relationship; individuals’ perception of their commitment to the intervention | Individual identification with intervention: A broad construct related to how individuals perceive the intervention, and their relationship and degree of commitment to the sustained use of the intervention.  *[revised to align with CFIR original language pulling from stage of change]* |
| **Characteristics of individuals**  Other Personal Attributes: A broad construct to include other personal traits such as tolerance of ambiguity, intellectual ability, motivation, values, competence, capacity, and learning style. | Individual orientation: Personal traits such as tolerance of ambiguity, team player, flexibility, problem solving, critical thinking |  |  |
|  | SYSTEMS LENS (change dynamics) | SYSTEMS LENS (change dynamics) |  |
| **Characteristics of individuals**  Other Personal Attributes: A broad construct to include other personal traits such as tolerance of ambiguity, intellectual ability, motivation, values, competence, capacity, and learning style. | Individual state of change: Phase an individual is in as he or she progresses toward skilled, enthusiastic, and sustained use of the intervention | Individual orientation to collaboration: Personal traits such as tolerance of ambiguity, team player, flexibility, problem solving, critical thinking | Individual orientation to collaboration: Personal traits such as tolerance of ambiguity, team player, flexibility, problem solving, critical thinking  *No change* |

REFERENCES

George, A. & al., e. 2019 Lenses and levels: the why, what and how of measuring health system drivers of women’s, children’s and adolescents’ health with a governance focus. *BMJ Glob Health,* 4.

Kinney, M. V., Walugembe, D. R., Wanduru, P., Waiswa, P. & George, A. S. 2019. Implementation of maternal and perinatal death reviews: a scoping review protocol. *BMJ Open,* 9**,** e031328.

1. The intervention is MPDSR or any related form of maternal and/or perinatal death review or audit. For the purposes of this review, we have used the broader term MPDSR. [↑](#footnote-ref-1)
