# Supplementary material for: Maternal and perinatal death surveillance and response in low- and middle-income countries: a scoping review of implementation factors
Source: Health Policy Plan. 2021 Mar 13;36(6):955–73. doi: 10.1093/heapol/czab011 (PMC8227470; doi:10.1093/heapol/czab011)
Supplement: czab011_Supp [file czab011_supp.zip › Supplementary 3.docx]

Supplementary 3: Methods

Contents

[Table S3.1: Preferred Reporting Items for Systematic reviews and Meta-Analyses extension for Scoping Reviews (PRISMA-ScR) Checklist 1](#_Toc52121802)

[Determining search terms 4](#_Toc52121803)

[Table S3.2: Results from testing search terms in PubMed 4](#_Toc52121804)

[Table S3.3: Components of data extraction tool 6](#_Toc52121805)

[Table S3.4: Record of consultation process 11](#_Toc52121806)

## Table S3.1: Preferred Reporting Items for Systematic reviews and Meta-Analyses extension for Scoping Reviews (PRISMA-ScR) Checklist

| **SECTION** | **ITEM** | **PRISMA-ScR CHECKLIST ITEM** | **REPORTED ON PAGE #** |
| --- | --- | --- | --- |
| **TITLE** | | | |
| Title | 1 | Identify the report as a scoping review. | 1 |
| **ABSTRACT** | | | |
| Structured summary | 2 | Provide a structured summary that includes (as applicable): background, objectives, eligibility criteria, sources of evidence, charting methods, results, and conclusions that relate to the review questions and objectives. | 2 |
| **INTRODUCTION** | | | |
| Rationale | 3 | Describe the rationale for the review in the context of what is already known. Explain why the review questions/objectives lend themselves to a scoping review approach. | 4 |
| Objectives | 4 | Provide an explicit statement of the questions and objectives being addressed with reference to their key elements (e.g., population or participants, concepts, and context) or other relevant key elements used to conceptualize the review questions and/or objectives. | 6 |
| **METHODS** | | | |
| Protocol and registration | 5 | Indicate whether a review protocol exists; state if and where it can be accessed (e.g., a Web address); and if available, provide registration information, including the registration number. | 5-6 |
| Eligibility criteria | 6 | Specify characteristics of the sources of evidence used as eligibility criteria (e.g., years considered, language, and publication status), and provide a rationale. | 6 |
| Information sources* | 7 | Describe all information sources in the search (e.g., databases with dates of coverage and contact with authors to identify additional sources), as well as the date the most recent search was executed. | 6 |
| Search | 8 | Present the full electronic search strategy for at least 1 database, including any limits used, such that it could be repeated. | 6 |
| Selection of sources of evidence† | 9 | State the process for selecting sources of evidence (i.e., screening and eligibility) included in the scoping review. | 7 |
| Data charting process‡ | 10 | Describe the methods of charting data from the included sources of evidence (e.g., calibrated forms or forms that have been tested by the team before their use, and whether data charting was done independently or in duplicate) and any processes for obtaining and confirming data from investigators. | 7 |
| Data items | 11 | List and define all variables for which data were sought and any assumptions and simplifications made. | 7 |
| Critical appraisal of individual sources of evidence§ | 12 | If done, provide a rationale for conducting a critical appraisal of included sources of evidence; describe the methods used and how this information was used in any data synthesis (if appropriate). | n/a |
| Synthesis of results | 13 | Describe the methods of handling and summarizing the data that were charted. | 8 |
| **RESULTS** | | | |
| Selection of sources of evidence | 14 | Give numbers of sources of evidence screened, assessed for eligibility, and included in the review, with reasons for exclusions at each stage, ideally using a flow diagram. | 8-9 |
| Characteristics of sources of evidence | 15 | For each source of evidence, present characteristics for which data were charted and provide the citations. | 9-10 |
| Critical appraisal within sources of evidence | 16 | If done, present data on critical appraisal of included sources of evidence (see item 12). | n/a |
| Results of individual sources of evidence | 17 | For each included source of evidence, present the relevant data that were charted that relate to the review questions and objectives. | 10-28 |
| Synthesis of results | 18 | Summarize and/or present the charting results as they relate to the review questions and objectives. | 10 |
| **DISCUSSION** | | | |
| Summary of evidence | 19 | Summarize the main results (including an overview of concepts, themes, and types of evidence available), link to the review questions and objectives, and consider the relevance to key groups. | 28-35 |
| Limitations | 20 | Discuss the limitations of the scoping review process. | 36 |
| Conclusions | 21 | Provide a general interpretation of the results with respect to the review questions and objectives, as well as potential implications and/or next steps. | 37 |
| **FUNDING** | | | |
| Funding | 22 | Describe sources of funding for the included sources of evidence, as well as sources of funding for the scoping review. Describe the role of the funders of the scoping review. | 38 |

JBI = Joanna Briggs Institute; PRISMA-ScR = Preferred Reporting Items for Systematic reviews and Meta-Analyses extension for Scoping Reviews.

* Where *sources of evidence* (see second footnote) are compiled from, such as bibliographic databases, social media platforms, and Web sites.

† A more inclusive/heterogeneous term used to account for the different types of evidence or data sources (e.g., quantitative and/or qualitative research, expert opinion, and policy documents) that may be eligible in a scoping review as opposed to only studies. This is not to be confused with *information sources* (see first footnote).

‡ The frameworks by Arksey and O’Malley (6) and Levac and colleagues (7) and the JBI guidance (4, 5) refer to the process of data extraction in a scoping review as data charting*.*

§ The process of systematically examining research evidence to assess its validity, results, and relevance before using it to inform a decision. This term is used for items 12 and 19 instead of "risk of bias" (which is more applicable to systematic reviews of interventions) to include and acknowledge the various sources of evidence that may be used in a scoping review (e.g., quantitative and/or qualitative research, expert opinion, and policy document).

*From:* Tricco AC, Lillie E, Zarin W, O'Brien KK, Colquhoun H, Levac D, et al. PRISMA Extension for Scoping Reviews (PRISMAScR): Checklist and Explanation. Ann Intern Med. 2018;169:467–473. [doi: 10.7326/M18-0850](http://annals.org/aim/fullarticle/2700389/prisma-extension-scoping-reviews-prisma-scr-checklist-explanation).

# Determining search terms

We tested search terms to determine which would yield the most relevant results. MPDSR has evolved over time and taken on different names in different settings. Table S1.1 provides the results of search terms tested in PubMed. We found that Concept 1 AND concept 2 yielded smaller results and would thus likely be the studies we will be looking for. Searching on the detailed types of audit did not yield different results then when we just searched for audit or surveillance and response (option 3 vs Option 4). Adding “obstetric” to concept 1 doubled the results. We decided to exclude “obstetric” because papers on obstetric audits would be captured under “audit”. If we had added “obstetric”, it would have broadened the search beyond what we were looking for. Similarly, we tested inclusion of “neonatal”. A review of the 67 studies found with “neonatal” search term did not find any studies relevant to this review that would not have also been identified with other search terms “audit”, “perinatal” and “quality of care”.

## Table S3.2: Results from testing search terms in PubMed

| Search terms entered into PubMed | Results |
| --- | --- |
| OPTION 1 – all terms included as individual terms |  |
| **(Concept 1) AND (concept 2)** |  |
| (maternal OR perinatal OR mortality OR death OR fetal OR obstetric OR Stillbirth) AND (audit OR "surveillance and response") | 5208 |
| (maternal OR perinatal OR mortality OR death OR fetal OR obstetric stillbirth OR *neonatal OR newborn*) AND (audit OR "surveillance and response")  (("mothers"[MeSH Terms] OR "mothers"[All Fields] OR "maternal"[All Fields]) OR perinatal[All Fields] OR ("mortality"[Subheading] OR "mortality"[All Fields] OR "mortality"[MeSH Terms]) OR ("death"[MeSH Terms] OR "death"[All Fields]) OR ("fetus"[MeSH Terms] OR "fetus"[All Fields] OR "fetal"[All Fields]) OR obstetric[All Fields] OR ("stillbirth"[MeSH Terms] OR "stillbirth"[All Fields]) OR ("infant, newborn"[MeSH Terms] OR ("infant"[All Fields] AND "newborn"[All Fields]) OR "newborn infant"[All Fields] OR "neonatal"[All Fields]) OR ("infant, newborn"[MeSH Terms] OR ("infant"[All Fields] AND "newborn"[All Fields]) OR "newborn infant"[All Fields] OR "newborn"[All Fields])) AND (audit[All Fields] OR "surveillance and response"[All Fields]) AND ("2004/01/01"[PDAT] : "2018/07/31"[PDAT]) | 5824 |
| OPTION 2 – adapted terms from above list includes obstetric |  |
| **(Concept 1) AND (concept 2)** |  |
| ("maternal mortality" OR "perinatal death" OR "maternal death" OR "perinatal mortality" OR "fetal death" OR "stillbirth" OR "obstetric") **AND** (audit OR "surveillance and response") | 841 |
| ("maternal mortality" OR "perinatal death" OR "maternal death" OR "perinatal mortality" OR "neonatal death" OR "neonatal mortality" OR "fetal death" OR "stillbirth" OR "obstetric") AND (audit OR "surveillance and response") | 869 |
| **OPTION 3 – as per the above search terms in concept 1 and concept 2** |  |
| **(Concept 1) AND (concept 2)** |  |
| ("maternal mortality" OR "perinatal death" OR "maternal death" OR "perinatal mortality" OR "fetal mortality" OR "stillbirth") **AND** (audit OR "surveillance and response")  (maternal mortality OR perinatal death OR maternal death OR perinatal mortality OR fetal mortality OR stillbirth) **AND** (audit OR surveillance and response) | 434 |
| ("maternal mortality" OR "perinatal death" OR "maternal death" OR "perinatal mortality" OR "fetal mortality" OR "stillbirth" OR "neonatal death" OR "neonatal mortality") AND (audit OR "surveillance and response") | 470 |
| **OPTION 4 – as per the above search terms** |  |
| **(Concept 1) AND (concept 2)** |  |
| ("maternal mortality" OR "perinatal death" OR "maternal death" OR "perinatal mortality" OR "fetal mortality" OR "stillbirth") AND ("death audit" OR "clinical audit" OR "obstetric audit" OR "quality of care audit" OR "surveillance and response" OR "audit") AND (("2004/01/01"[PDat] : "2018/07/31"[PDat])) | 434 |
| ("neonatal death" OR "neonatal mortality" OR "maternal mortality" OR "perinatal death" OR "maternal death" OR "perinatal mortality" OR "fetal mortality" OR "stillbirth") AND ("death audit" OR "clinical audit" OR "obstetric audit" OR "quality of care audit" OR "surveillance and response" OR "audit") AND (("2004/01/01"[PDat] : "2018/07/31"[PDat])) | 470 |
| TEST OF NEONATAL and AUDIT |  |
| ("neonatal death" OR "neonatal mortality") AND ("death audit" OR "clinical audit" OR "obstetric audit" OR "quality of care audit" OR "surveillance and response" OR "audit") AND (("2004/01/01"[PDat] : "2018/07/31"[PDat])) | 67 |

## Table S3.3: Components of data extraction tool

|  | Reviewer assigned |  |
| --- | --- | --- |
|  | Inclusion | Yes  No |
| **Reference details** | Ref ID |  |
|  | Author |  |
|  | Title |  |
|  | Year |  |
|  | Abstract |  |
|  | Journal |  |
|  | Volume |  |
|  | Issue |  |
|  | Pages |  |
|  | URL |  |
|  | Source | Database  Online search  Consultation |
| Background to reference | Country of first affiliation |  |
|  | Country of Study | Name country  Multiple  N/A |
|  | Country Setting | Upper MI  Lower MI  LIC  LMIC  Not specified (add to notes) |
|  | Region of World | Sub-Saharan Africa  East Asia & Pacific  Latin America & Caribbean  South Asia  Europe & Central Asia  Middle East & North Africa  International |
|  | Organization Type of authors | Government  NGO  University/academic institution  Independent  Mixed including government  Mixed not including government  Other (add note) |
|  | Funder type (sector) | Government  Multilateral (e.g. WHO, World Bank, UN)  Bilateral (e.g. USAID, DIFD, IDRC)  Foundation (e.g. BMGF, Rockefeller)  Mixed includes government  Mixed does not include government  No funding received |
|  | Notes |  |
| ]Content of reference | Focus of the audit | Maternal & perinatal  Maternal  Perinatal |
|  | Type of audit | [written as described in paper e.g. MDR, MDSR, perinatal death audit, obstetric audit, etc..] |
|  | Audit cycle explained | Described  Not described |
|  | Notes |  |
|  | History/timeline | Yes  No |
|  | Notes |  |
|  | Scale | National  Subnational  Selected Facilities  Combination Of Levels  Global  Multicountry  Other |
|  | Notes |  |
| Reference type | Document type | Academic journal article  Academic journal commentary  Academic review  Grey lit online  Grey lit not online  other |
|  | Methods section | Yes  No |
|  | Research Design | Quantitative  Qualitative  Mixed  NA |
|  | Level of Study | Macro  Meso  Micro  Combination |
|  | Notes |  |
| Resource support | Pilot study | Yes  No |
|  | Notes |  |
|  | Completely donor funded | Yes  No  Not mentioned |
|  | Partly funded | Yes  No  Not mentioned |
| Steps of the audit cycle described | Notification | Yes  No |
|  | Data collection | Yes  No |
|  | Review process | Yes  No |
|  | Recommendations | Yes  No |
|  | Evaluation | Yes  No |
|  | Full process described | Yes  No |
| Domain 1: Intervention/MPDSR | Executing audit | Described  Not described |
|  | Notes |  |
|  | Cost & funding | Described  Not described |
|  | Notes |  |
|  | Intervention source | Described  Not described |
|  | Notes |  |
|  | Evidence strength & quality | Described  Not described |
|  | Notes |  |
|  | Relative advantage | Described  Not described |
|  | Notes |  |
|  | Trialability | Described  Not described |
|  | Notes |  |
|  |  |  |
|  |  |  |
|  | Adaptability | Described  Not described |
|  | Notes |  |
|  | Complexity | Described  Not described |
|  | Notes |  |
| Domain 2: Outer Setting | Policy and planning | Described  Not described |
|  | Notes |  |
|  | Resource flows | Described  Not described |
|  | Notes |  |
|  | External actors | Described  Not described |
|  | Notes |  |
|  | Political prioritization | Described  Not described |
|  | Notes |  |
|  | Pressure to implement | Described  Not described |
|  | Notes |  |
|  | Linkages and network | Described  Not described |
|  | Notes |  |
| Domain 3: Inner Setting | Readiness to implement | Described  Not described |
|  | Notes |  |
|  | Team composition | Described  Not described |
|  | Notes |  |
|  | Incentive/rewards | Described  Not described |
|  | Notes |  |
|  | Team relationship | Described  Not described |
|  | Notes |  |
|  |  |  |
|  |  |  |
|  | Implementation culture and climate | Described  Not described |
|  | Notes |  |
|  | Engaged leaders | Described  Not described |
|  | Notes |  |
| Domain 4: Individuals | Tech skills & knowledge | Described  Not described |
|  | Notes |  |
|  | Self-efficacy | Described  Not described |
|  | Notes |  |
|  | Individual motivation | Described  Not described |
|  | Notes |  |
|  | Individual identification with intervention | Described  Not described |
|  | Notes |  |
|  | Individual orientation | Described  Not described |
|  | Notes |  |
|  |  |  |
|  |  |  |

## Table S3.4: Record of consultation process

| **Date** | **Event** | **Purpose** | **Participants** |
| --- | --- | --- | --- |
| September 19, 2018 | Countdown to 2030 Drivers Group meeting | To introduce idea of scoping review and presentation of draft conceptual framework | Asha George, David Sanders, Peter Waiswa, Luis Huicho, Shehla Zaidi, Josephine Borghi, Neha Singh, Anne Haakenstaad, Rajani Ved, Kent Buse, Kumanan Rasanathan, Rajat Khosla, Bernadette Daelmans and Troy Jacobs |
| November 7, 2018 | MPDSR TWG meeting | To introduce idea of scoping review and request for relevant literature | Allisyn Moran, Nathalie Roos, Ravi Jeganathan, Neena Khadka, Rebecca Levine, Mary Kinney, Hannah Blencowe, Subha Sri Balakrishnan, Matthews, Diane Morof, Robyn Churchill, Doris Chou, Louise Tina-Day, Sylvia Alford, Sara Nam. |
| 19-23 November 2018 | Maternal Perinatal Death Surveillance and Response Capacity Building: Joint WHO/UNFPA/UNICEF Workshop, | To introduce idea of scoping review and request for relevant literature | Over 100 participants from 32 countries (Angola, Benin, Botswana, Burkina Faso, Burundi, Cameroun, Chad, Congo Brazaville, Cote D’Ivoire, DRC, Eswatini, Ethiopia, Ghana, Guinea, Kenya, Liberia, Madagascar, Malawi, Mozambique, Namibia, Niger, Nigeria, Rwanda, Senegal, Sierra Leone, South Africa, South Sudan, Tanzania, Togo, Uganda, Zambia, Zimbabwe) from East and Southern Africa Ministries of Health, UNICEF, UNFPA and WHO country, regional and global offices |
| November 29, 2018 | Webinar on scoping review to MPDSR TWG | To present draft protocol and conceptual framework with discussion. Call for available literature | Luc de Bernis, Louise-Tina Day, Sabry Hamza, Asha George, Phillip Wanduru, David Walugembe |
| January 23, 2019 | Email exchange | To introduce idea of scoping review and request for relevant literature | Helen Smith, Marge Koblinksy |
| January 24, 2019 | Email exchange | To introduce idea of scoping review and request for relevant literature | Ank de Jonge |
| January 29, 2019 | Countdown to 2030 Drivers Group meeting | To update on status of review | Asha George, David Sanders, Peter Waiswa, Luis Huicho, Shehla Zaidi, Josephine Borghi, Neha Singh, Anne Haakenstaad, Rajani Ved, Kent Buse, Kumanan Rasanathan, Rajat Khosla, Bernadette Daelmans and Troy Jacobs |
| March 26, 2019 | Meeting (call) with members of the MPDSR TWG | To discuss on the scoping review protocol and conceptual framework as well as available literature | Sarah Nam and Ashiru Hamza |
| April 18, 2019 | Email exchange | To introduce idea of scoping review and request for relevant literature | Andre Lalonde, Sara Nam |
| June 10, 2019 | University of the Western Cape, School of Public Health, Journal club | To present protocol and conceptual framework with discussion. | Asha George, Phillip Wanduru, David Walugembe |
| December 9, 2019 | Countdown to 2030 Drivers Group meeting | To present revised framework | Asha George, David Sanders, Peter Waiswa, Luis Huicho, Shehla Zaidi, Josephine Borghi, Neha Singh, Anne Haakenstaad, Rajani Ved, Kent Buse, Kumanan Rasanathan, Rajat Khosla, Bernadette Daelmans and Troy Jacobs |
| April 28, 2020 | MPDSR TWG meeting | To share preliminary results of scoping review | Allisyn Moran, Nathalie Roos, Ravi Jeganathan, Neena Khadka, Rebecca Levine, Mary Kinney, Hannah Blencowe, Subha Sri Balakrishnan, Matthews, Diane Morof, Robyn Churchill, Doris Chou, Louise Tina-Day, Sylvia Alford, Sara Nam. |
