# Supplementary material for: Maternal and perinatal death surveillance and response in low- and middle-income countries: a scoping review of implementation factors
Source: Health Policy Plan. 2021 Mar 13;36(6):955–73. doi: 10.1093/heapol/czab011 (PMC8227470; doi:10.1093/heapol/czab011)
Supplement: czab011_Supp [file czab011_supp.zip › Supplementary 4.docx]

Supplementary 4: Results

Contents

[Table S4.1: Characteristics of included studies (ordered by year) 1](#_Toc52122318)

[Table S4.2: Summary of data points by domain, construct and lens 9](#_Toc52122319)

[Table S4.3: Summary of data points by domain and lens 10](#_Toc52122320)

[Table S4.4: Detailed results by construct 11](#_Toc52122321)

[Table S4.5. Components of the audit cycle described 33](#_Toc52122322)

## Table S4.1: Characteristics of included studies (ordered by year)

| **Author and year** | **Country of focus** | **Region of world** | **Country of first affiliation** | **Type of audit** | **Document type (research design)** | **Level of Study** | **Scale** |
| --- | --- | --- | --- | --- | --- | --- | --- |
| ***Studies*** |  |  |  |  |  |  |  |
| Manandhar 2004,  (Manandhar 2004) | Nepal | South Asia | Nepal | perinatal death audit | Academic journal article (not indicated) | Macro | Other |
| Day 2006,  (Day 2006) | Bangladesh | South Asia | Bangladesh | perinatal and maternal death audit | Grey literature not online (mixed) | Combination | Combination of levels |
| Mogobe et al. 2007,  (Mogobe et al. 2007) | Botswana | Sub-Saharan Africa | Botswana | maternal death and morbidity reviews | Academic journal article (quantitative) | Macro | National |
| Muffler et al. 2007,  (Muffler et al. 2007) | Morocco | Middle East & North Africa | Germany | mix | Academic journal article (mixed) | Combination | Combination of levels |
| Kongnyuy and van den Broek 2008,  (Kongnyuy and van den Broek 2008) | Malawi | Sub-Saharan Africa | UK | MDR | Academic journal article (qualitative) | Meso | National |
| Dumont et al. 2009,  (Dumont et al. 2009) | Senegal | Sub-Saharan Africa | Senegal | MDR | Academic journal article (mixed) | Combination | Selected facilities |
| Pearson et al. 2009,  (Pearson et al. 2009) | Multicountry | Sub-Saharan Africa | Kenya | MDR | Academic journal article (qualitative) | Macro | National |
| Richard et al. 2009,  (Richard et al. 2009) | Burkina Faso | Sub-Saharan Africa | Belgium | medical audit | Academic journal article (qualitative) | Combination | Selected facilities |
| Nyamtema et al. 2010,  (Nyamtema et al. 2010) | South Africa | Sub-Saharan Africa | Tanzania | maternal and perinatal death audits | Academic journal article (mixed) | Combination | Selected facilities |
| Bergh et al. 2011,  (Bergh et al. 2011) | Tanzania | Sub-Saharan Africa | South Africa | perinatal death audit program | Grey literature not online (qualitative) | Combination | National |
| Bakker et al. 2011,  (Bakker et al. 2011) | Malawi | Sub-Saharan Africa | Netherlands | obstetric Critical incident audit | Academic journal article (qualitative) | Combination | Subnational |
| Belizan et al. 2011,  (Belizan et al. 2011) | South Africa | Sub-Saharan Africa | South Africa | perinatal death audit program | Academic journal article (qualitative) | Combination | National |
| Nyamtema et al. 2011,  (Nyamtema et al. 2011) | Multicountry | Sub-Saharan Africa | Tanzania | maternal death and morbidity reviews | Academic journal article (mixed) | Micro | Selected facilities |
| Grellier and Shome 2011  (Grellier and Shome 2011) | Uganda | Mixed | UK | mix | Grey literature not online (mixed) | Combination | Combination of levels |
| Nam 2011  (Nam 2011) | Tanzania | Sub-Saharan Africa | UK | MDR | Grey literature not online (mixed) | Combination | Selected facilities |
| WHO 2011  (WHO 2011) | Lebanon | Middle East & North Africa | Lebanon | maternal, perinatal, neonatal health surveillance systems | Grey literature online (not indicated) | Macro | National |
| van Hamersveld et al. 2012,  (van Hamersveld et al. 2012) | Ghana | Sub-Saharan Africa | Tanzania | obstetric audit | Academic journal article (qualitative) | Meso | Selected facilities |
| Dartey 2012,  (Dartey 2012) | NA | Sub-Saharan Africa | South Africa | MDR | Grey literature online (qualitative) | Combination | Selected facilities |
| E4A 2012,  (E4A 2012) | Tanzania | NA | UK | MDSR | Grey literature online (not indicated) | Macro | National |
| Dumont et al. 2013,  (Dumont et al. 2013) | Senegal | Sub-Saharan Africa | France | MDR | Academic journal article (mixed) | Combination | Selected facilities |
| WHO 2013,  (WHO 2013) | Multicountry | Sub-Saharan Africa | Morocco | MDSR | Grey literature online (not indicated)online (not indicated) | Macro | National |
| Armstrong et al. 2014,  (Armstrong et al. 2014) | Tanzania | Sub-Saharan Africa | UK | MPDR | Academic journal article (qualitative) | Combination | Combination of levels |
| Balogun and Musoke 2014,  (Balogun and Musoke 2014) | Sudan | Sub-Saharan Africa | Sweden | MDR | Academic journal article (qualitative) | Combination | Combination of levels |
| Combs Thorsen et al. 2014,  (Combs Thorsen et al. 2014) | Malawi | Sub-Saharan Africa | Norway | MDR | Academic journal article (qualitative) | Combination | Selected facilities |
| Hofman and Mohammed 2014,  (Hofman and Mohammed 2014) | Nigeria | Sub-Saharan Africa | UK | MDR | Academic journal article (mixed) | Combination | Selected facilities |
| Lewis 2014b,  (Lewis 2014b) | Multicountry | International | UK | maternal death and near-death reviews | Academic journal article (not indicated) | Combination | Multicountry |
| Purandare et al. 2014,  (Purandare et al. 2014) | India | International | India | MDR | Academic journal article (mixed) | Macro | National |
| Rhoda et al. 2014,  (Rhoda et al. 2014) | South Africa | Sub-Saharan Africa | South Africa | perinatal death audit | Academic journal article (not indicated) | Combination | Combination of levels |
| WHO 2014a,  (WHO 2014a) | Indonesia | South Asia | India | mix | Grey literature online (qualitative) | Combination | Combination of levels |
| WHO 2014b,  (WHO 2014b) | Indonesia | East Asia & Pacific | Indonesia | maternal death reviews | Grey literature online (qualitative) | Combination | Combination of levels |
| WHO 2014c,  (WHO 2014c) | Nepal | South Asia | Nepal | MDR | Grey literature online (qualitative) | Combination | Combination of levels |
| WHO 2014d,  (WHO 2014d) | Sri Lanka | South Asia | Sri Lanka | mix | Grey literature online (qualitative) | Combination | Combination of levels |
| WHO 2014e,  (WHO 2014e) | Multicounty | International | NA | mix | Grey literature online (qualitative) | Combination | Combination of levels |
| Bayley et al. 2015,  (Bayley et al. 2015) | Malawi | Sub-Saharan Africa | UK | CLMDR | Academic journal article (mixed) | Meso | Subnational |
| Biswas et al. 2015,  (Biswas et al. 2015) | Bangladesh | South Asia | Sweden | facility maternal and neonatal death review | Academic journal article (qualitative) | Meso | Subnational |
| Kerber et al. 2015,  (Kerber et al. 2015) | Multicounty | International | USA | perinatal death audit | Academic journal article (qualitative) | Macro | Multicountry |
| Magoma et al. 2015  (Magoma et al. 2015) | Tanzania | Sub-Saharan Africa | Tanzania | MDR | Academic journal article (quantitative) | Micro | Selected facilities |
| Agaro et al. 2016,  (Agaro et al. 2016) | Uganda | Sub-Saharan Africa | Uganda | MPDR | Academic journal article (mixed) | Meso | Subnational |
| Bandali et al. 2016,  (Bandali et al. 2016) | Multicounty | International | UK | MDSR | Academic journal article (mixed) | Combination | Combination of levels |
| Hadush et al. 2016,  (Hadush et al. 2016) | Ethiopia | International | Ethiopia | MDSR | Grey literature online (not indicated) | Combination | Global |
| Ethiopia Federal Ministry of Health et al. 2016,  (Ethiopia Federal Ministry of Health et al. 2016) | Ethiopia | Sub-Saharan Africa | NA | MDSR | Grey literature online (not indicated) | Combination | National |
| WHO 2016,  (WHO 2016) | Maldives | South Asia | Maldives | MPDSR | Grey literature online (not indicated) | Combination | National |
| Abebe et al. 2017  (Abebe et al. 2017) | Ethiopia | Sub-Saharan Africa | Ethiopia | MDSR | Academic journal article (qualitative) | Combination | Combination of levels |
| Congo et al. 2017,  (Congo et al. 2017) | Burkina Faso | Sub-Saharan Africa | Burkina Faso | maternal death audits | Academic journal article (qualitative) | Combination | Subnational |
| de Kok et al. 2017,  (de Kok et al. 2017) | Nigeria | Sub-Saharan Africa | UK | MDR | Academic journal article (qualitative) | Meso | Selected facilities |
| Mutsigiri-Murewanhema et al. 2017,  (Mutsigiri-Murewanhema et al. 2017) | Zimbabwe | Sub-Saharan Africa | Zimbabwe | maternal mortality surveillance system | Academic journal article (mixed) | Meso | Selected facilities |
| Smith et al. 2017b,  (Smith et al. 2017b) | Kenya | Sub-Saharan Africa | UK | MDSR | Academic journal article (not indicated) | Macro | National |
| Smith et al. 2017c,  (Smith et al. 2017c) | Multicounty | International | UK | MDSR | Academic journal article (not indicated) | Macro | National |
| Tapesana et al. 2017,  (Tapesana et al. 2017) | Zimbabwe | sub-Saharan Africa | Zimbabwe | MDSR | Academic journal article (qualitative) | Meso | Combination of levels |
| Koblinsky et al. 2017,  (Koblinsky et al. 2017) | Indonesia | East Asia & Pacific | Indonesia | MPDSR | Grey literature not online (not indicated) | Combination | Combination of levels |
| Ministry of Health and Sanitation [Sierra Leone] 2017,  (Ministry of Health and Sanitation [Sierra Leone] 2017) | Sierra Leone | Sub-Saharan Africa | Sierra Leone | MDSR | Grey literature online (mixed) | Combination | National |
| Ethiopian Public Health Institute et al. 2017,  (Ethiopian Public Health Institute et al. 2017) | Ethiopia | Sub-Saharan Africa | Ethiopia | MDSR | Grey literature online (not indicated) | Macro | National |
| MCSP 2017c,  (MCSP 2017c) | Zimbabwe | Sub-Saharan Africa | USA | MPDSR | Grey literature online (qualitative) | Combination | Combination of levels |
| MCSP 2017b,  (MCSP 2017b) | Nigeria | Sub-Saharan Africa | USA | MPDSR | Grey literature online (qualitative) | Combination | Combination of levels |
| MCSP 2017a,  (MCSP 2017a) | Rwanda | Sub-Saharan Africa | USA | MPDSR | Grey literature online (qualitative) | Combination | Combination of levels |
| Karamagi et al. 2018,  (Karamagi et al. 2018) | Uganda | Sub-Saharan Africa | Uganda | MPDSR | Grey literature online (mixed) | Combination | Selected facilities |
| MCSP 2018,  (MCSP 2018) | Tanzania | Sub-Saharan Africa | USA | MPDSR | Grey literature online (qualitative) | Combination | Combination of levels |
| du Châtelet et al. 2019 [unpublished],  (du Châtelet et al. 2019 [unpublished]) | Sierra Leone | Sub-Saharan Africa | Sierra Leone | MDR | Grey literature not online (qualitative) | Combination | Multicountry |
| ***Commentaries*** |  |  |  |  |  |  |  |
| Kongnyuy and van den Broek 2009  (Kongnyuy and van den Broek 2009) | 2009 | International | UK | clinical audit | Academic journal commentary | Combination | Other |
| Baleta 2011  (Baleta 2011) | South Africa | Sub-Saharan Africa | South Africa | perinatal death audit program | Academic journal commentary | Macro | National |
| Buchmann 2014  (Buchmann 2014) | N/A | International | South Africa | perinatal death audit | Academic journal commentary | Combination | Combination of levels |
| De Brouwere et al. 2014  (De Brouwere et al. 2014) | Cameroon | Sub-Saharan Africa | Belgium | MDR | Academic journal commentary | Combination | National |
| Lewis 2014a  (Lewis 2014a) | N/A | International | UK | MDR | Academic journal commentary | Combination | Global |
| Scott and Danel 2016  (Scott and Danel 2016) | N/A | International | Canada | mix | Academic journal commentary | Macro | Multicountry |
| Koblinsky 2017  (Koblinsky 2017) | Kenya | Sub-Saharan Africa | USA | MDSR | Academic journal commentary | Macro | Multicountry |
| Smith et al. 2017a  (Smith et al. 2017a) | Kenya | International | UK | MDSR | Academic journal commentary | Macro | Combination of levels |
| ***Reviews*** |  |  |  |  |  |  |  |
| Pattinson et al. 2005  (Pattinson et al. 2005) | N/A | International | South Africa | maternal and perinatal death reviews | Academic review | Combination | Multicountry |
| Pattinson et al. 2009  (Pattinson et al. 2009) | N/A | International | South Africa | perinatal death audit | Academic review | Combination | Multicountry |
| Raven et al. 2011  (Raven et al. 2011) | N/A | International | UK | maternal and perinatal death reviews | Academic review | Combination | Combination of levels |
| Hussein et al. 2016  (Hussein et al. 2016) | Nigeria | Sub-Saharan Africa | UK | MDR and obstetric audit | Academic review | Combination | Selected facilities |
| Martin Hilber et al. 2016  (Martin Hilber et al. 2016) | N/A | Sub-Saharan Africa | Switzerland | mix | Academic review | Combination | Combination of levels |
| Biswas 2017  (Biswas 2017) | Bangladesh | South Asia | Bangladesh | MPDR | Academic review | Combination | National |

## Table S4.2: Summary of data points by domain, construct and lens

| **Number of constructs described by domain** | | | | |  |  | **Total number (%)** |
| --- | --- | --- | --- | --- | --- | --- | --- |
| *Domain 1: Intervention/MPDSR* | | | | | | | |
| Components and execution | Cost | Framing - Intervention source | Framing - Evidence strength & quality | Framing -Relative advantage | Trialability | Adaptability |  |
| 34 | 34 | 41 | 31 | 0 | 22 | 15 | 177 (29%) |
| *Domain 2: Outer Setting* | | | | | | | |
| Policy and planning | Resource support | External actors | Political prioritization | Pressure to implement | Linkages and networks between levels |  |  |
| 41 | 29 | 31 | 10 | 17 | 34 |  | 162 (27%) |
| *Domain 3: Inner Setting* | | | | | | | |
| Readiness to implement | Team composition and characteristics | Organizational incentive/rewards | Team relationships | Implementation culture & climate | Engaged leaders |  |  |
| 48 | 36 | 11 | 19 | 47 | 21 |  | 182 (30%) |
| *Domain 4: Individuals* | | | | | | | |
| Tech skills & knowledge | Self-efficacy | Individual motivation | Individual identification with intervention | Individual orientation to collaboration |  |  |  |
| 31 | 8 | 23 | 18 | 0 |  |  | 80 (13%) |

## Table S4.3: Summary of data points by domain and lens

| **Lens** | **Domain 1** | **Domain 2** | **Domain 3** | **Domain 4** | **total** | **% of total** |
| --- | --- | --- | --- | --- | --- | --- |
| Service delivery | 68 | 70 | 95 | 31 | 264 | 44% |
| Societal | 72 | 41 | 19 | 49 | 181 | 30% |
| Systems | 37 | 51 | 68 | 0 | 156 | 26% |
| *Total* | *177* | *162* | *182* | *80* | *601* |  |

## Table S4.4: Detailed results by construct

| **Domain** | **Construct** | **Records** | **Detailed findings** |
| --- | --- | --- | --- |
| Domain 1: Intervention/ MPDSR | Components and execution | Abebe et al. 2017,  Agaro et al. 2016,  Armstrong et al. 2014,  Bakker et al. 2011,  Balogun and Musoke 2014,  Bandali et al. 2016,  Bayley et al. 2015,  Belizan et al. 2011,  Bergh et al. 2011,  Biswas et al. 2015,  Combs Thorsen et al. 2014,  Congo et al. 2017,  Dartey 2012,  Day 2006,  Ministry of Health and Sanitation [Sierra Leone] 2017,  du Châtelet et al. 2019 [unpublished],  Hofman and Mohammed 2014,  Kerber et al. 2015,  Koblinsky et al. 2017,  MCSP 2017c,  MCSP 2017b,  MCSP 2017a,  MCSP 2018,  Muffler et al. 2007,  Mutsigiri-Murewanhema et al. 2017,  Nyamtema et al. 2010,  Purandare et al. 2014,  Rhoda et al. 2014,  Smith et al. 2017b,  Smith et al. 2017c,  Tapesana et al. 2017,  van Hamersveld et al. 2012,  WHO 2014d,  WHO 2013, | - Most studies explained the intervention process, or audit cycle, in theory (74%). Yet there was uneven reporting between the different components; only 29% reported on all components of the audit cycle. (Table S4:5). - Our mapping of the audit cycle steps found that over half of the studies described the data collection process (52%), the review process (53%), and the recommendation process (52%). Fewer records reported on the notification process and the evaluation of the process (or feedback loop) (40% for both). - Many studies reported on multiple steps (60%), and described the implementation factors of these steps, linked to other constructs in this framework (e.g. skills of health workers, cost, enabling environment). - There were no patterns observed between study types and or level of studies. None of the studies compared the implementation characteristics of the different audit cycle steps with each other. - The evolution of the intervention process over time: The studies prior to 2011 were all maternal and/or perinatal death reviews. A WHO regional report was the first to show expansion of the maternal and/or perinatal death reviews to include surveillance in 2011 (WHO 2011); The first study to present MPDSR was in another WHO regional report was the first to present a combined approach for MPDSR in 2016, (WHO 2016). From 2016, until 2018, seven of 21 studies used the term MPDSR to describe the intervention (WHO 2016, MCSP 2017c, MCSP 2017a, MCSP 2017b, MCSP 2018, Koblinsky et al. 2017, Karamagi et al. 2018); though most noted that the perinatal component was aspirational. Four studies, during this time period, still focused on maternal death review (without surveillance) (WHO 2014c, du Châtelet et al. 2019 [unpublished], de Kok et al. 2017, Congo et al. 2017). We did not find any differences in implementation factors between the different types of reviews, e.g. maternal death review, perinatal death audit, MDSR, or MPDSR. |
|  | Cost | Abebe et al. 2017,  Agaro et al. 2016,  Armstrong et al. 2014,  Balogun and Musoke 2014,  Bandali et al. 2016,  Belizan et al. 2011,  Congo et al. 2017,  Dartey 2012,  Day 2006,  du Châtelet et al. 2019 [unpublished],  Hadush et al. 2016,  Ethiopia Federal Ministry of Health et al. 2016,  Hofman and Mohammed 2014,  Kerber et al. 2015,  Koblinsky et al. 2017,  Kongnyuy and van den Broek 2008,  Magoma et al. 2015  MCSP 2017c,  MCSP 2017a,  Mutsigiri-Murewanhema et al. 2017,  Nyamtema et al. 2011,  Nyamtema et al. 2010,  Pearson et al. 2009,  Rhoda et al. 2014,  Richard et al. 2009,  Smith et al. 2017b,  Smith et al. 2017c,  Tapesana et al. 2017,  van Hamersveld et al. 2012,  WHO 2014d,  WHO 2014b,  WHO 2014e,  WHO 2014c,  WHO 2016, | - Resource support for M/PDSR was noted as an important facilitator for implementation (Kerber et al. 2015, Smith et al. 2017c, WHO 2016); yet the literature showed mixed findings on whether specifically allocated resources are needed for M/PDSR and if so, how much and how it is budgeted (Magoma et al. 2015, Pearson et al. 2009, Koblinsky et al. 2017, Bandali et al. 2016, Rhoda et al. 2014, Koblinsky et al. 2017, WHO 2014d, Belizan et al. 2011, Balogun and Musoke 2014, Hadush et al. 2016, Kongnyuy and van den Broek 2008, WHO 2014b, WHO 2014c, Hofman and Mohammed 2014, Baleta 2011, Congo et al. 2017, MCSP 2017a, Magoma et al. 2015, Koblinsky et al. 2017). - Costs relating to the audit and reporting process, such as collecting data, meeting related costs (i.e. transport, specific training, running secretariat, time), information systems, etc, were often described as a barrier (Abebe et al. 2017, Agaro et al. 2016, Armstrong et al. 2014, Balogun and Musoke 2014, Bandali et al. 2016, Congo et al. 2017, Dartey 2012, Hadush et al. 2016, Ethiopia Federal Ministry of Health et al. 2016, Hofman and Mohammed 2014, Kerber et al. 2015, MCSP 2017c, MCSP 2017a, Mutsigiri-Murewanhema et al. 2017, Nyamtema et al. 2011, Nyamtema et al. 2010,Rhoda et al. 2014, Smith et al. 2017b, Smith et al. 2017c, Tapesana et al. 2017, van Hamersveld et al. 2012, WHO 2016, Koblinsky et al. 2017, du Châtelet et al. 2019 [unpublished], Richard et al. 2009, Pearson et al. 2009, WHO 2014b, WHO 2014c, WHO2014e). Overall human resources were considered as part of the cost to the implementation process, such as staff workload, staff shortages, staff turnover and staff skills (3, Bakker et al. 2011, Dartey 2012, De Brouwere et al. 2014, Ethiopia Federal Ministry of Health et al. 2016, Hofman and Mohammed 2014, Kerber et al. 2015, Koblinsky 2017, Lewis 2014b, MCSP 2017c, Mutsigiri-Murewanhema et al. 2017, Pattinson et al. 2009, Smith et al. 2017b, WHO 2014e, Richard et al. 2009). - Several studies specifically mentioned the challenge of not having funds to implement recommendations from the audit process (Kongnyuy and van den Broek 2008, MCSP 2017c, Smith et al. 2017b, van Hamersveld et al. 2012, Koblinsky et al. 2017, du Châtelet et al. 2019 [unpublished], WHO 2014e). - Only one study reported that the cost of notification of a maternal death was justifiable (Tapesana et al. 2017). - Several studies specifically mentioned the challenge of not having funds to implement recommendations from the audit process (Kongnyuy and van den Broek 2008, MCSP 2017c, Smith et al. 2017b, van Hamersveld et al. 2012, Koblinsky et al. 2017, du Châtelet et al. 2019 [unpublished], WHO 2014e). This is illustrated by this quote: "You can recommend something and administrators say that they have no money.” (van Hamersveld et al. 2012) Given these experiences, some argue that a minimum level of human and material resources is required before the system implements M/PDSR (Richard et al. 2009, Koblinsky 2017). - A study from South Africa estimated the annual cost of running the Perinatal Priority Identify Programme at $35,000 per year, which included software program maintenance and development costs, office running costs, collating data, reports and attendance at subnational and national meetings (Pattinson et al. 2009). A study from Cameroon estimated US$ 1.5 million for training of ten regional MDR teams and projected an annual running cost of these regional MDR committees at $Belizan et al. 2011,0 per year (De Brouwere et al. 2014). A study from Zimbabwe provided a full breakdown of the total running cost per institutional maternal death notified (USD$246) including staff time, communication and fuel expenses (for sending completed forms and for health workers to attend review meeting)costs including human resource (Tapesana et al. 2017). |
|  | Framing - Intervention source | Abebe et al. 2017,  Agaro et al. 2016,  Armstrong et al. 2014,  Bakker et al. 2011,  Balogun and Musoke 2014,  Bandali et al. 2016,  Belizan et al. 2011,  Biswas et al. 2015,  Congo et al. 2017,  Dartey 2012,  Ministry of Health and Sanitation [Sierra Leone] 2017,  Dumont et al. 2009,  Ethiopia Federal Ministry of Health et al. 2016,  Ethiopian Public Health Institute et al. 2017  Hofman and Mohammed 2014,  Kerber et al. 2015,  Kongnyuy and van den Broek 2008,  Lewis 2014b,  MCSP 2017c,  MCSP 2017b,  MCSP 2017a,  MCSP 2018,  Mogobe et al. 2007,  Mutsigiri-Murewanhema et al. 2017,  Nyamtema et al. 2011,  Purandare et al. 2014,  Rhoda et al. 2014,  Smith et al. 2017c,  WHO 2011  WHO 2013,  WHO 2016,  Koblinsky et al. 2017,  Day 2006,  du Châtelet et al. 2019 [unpublished],  Richard et al. 2009,  Bergh et al. 2011,  WHO 2014a,  WHO 2014b,  WHO 2014e,  WHO 2014d,  WHO 2014c, | - The identified records did not explore stakeholder perceptions of legitimacy related to whether the intervention was internally or externally developed. - A study from South Africa found respondents felt the interventions was “top down” without ownership at the facility level (Belizan et al. 2011, Bergh et al. 2011); as was found in another study from Sudan (Balogun and Musoke 2014) - The framing of the intervention was described in a number of studies as government initiated (3, Agaro et al. 2016, Balogun and Musoke 2014, Biswas et al. 2015, Ministry of Health and Sanitation [Sierra Leone] 2017, Ethiopian Public Health Institute et al. 2017 Ethiopia Federal Ministry of Health et al. 2016, MCSP 2017b, WHO 2014e, WHO 2014d, WHO 2014c), externally driven by partners (Purandare et al. 2014, Rhoda et al. 2014, Smith et al. 2017c, Mutsigiri-Murewanhema et al. 2017, WHO 2013, Koblinsky et al. 2017, Day 2006, du Châtelet et al. 2019 [unpublished], Bergh et al. 2011, Dumont et al. 2009, WHO 2011, WHO 2016, Richard et al. 2009, WHO 2014a, WHO2014b), or legitimate due to the embedded nature of the intervention (Bakker et al. 2011, Dartey 2012, Mogobe et al. 2007, Congo et al. 2017). - Some countries have applied and adapted their approaches from the global WHO guidelines (Abebe et al. 2017, Biswas et al. 2015, Smith et al. 2017c, Scott and Danel 2016, WHO 2014b, Armstrong et al. 2014, Hofman and Mohammed 2014, MCSP 2017c, MCSP 2017a, MCSP 2018, Kongnyuy and van den Broek 2008, Lewis 2014b,), with some studies recognizing the importance of global guidelines in standardizing national practice (Smith et al. 2017b, Scott and Danel 2016). One study initiated from within a facility without influence from external partners (including Ministry of Health) (Nyamtema et al. 2011). |
|  | Framing - Evidence strength & quality | Abebe et al. 2017,  Agaro et al. 2016,  Armstrong et al. 2014,  Bakker et al. 2011,  Bandali et al. 2016,  Belizan et al. 2011,  Biswas et al. 2015,  Dartey 2012,  Ethiopian Public Health Institute et al. 2017,  Hofman and Mohammed 2014,  Kerber et al. 2015,  Lewis 2014b,  MCSP 2017c,  MCSP 2017b,  MCSP 2017a,  MCSP 2018,  Mutsigiri-Murewanhema et al. 2017,  Nyamtema et al. 2011,  Rhoda et al. 2014,  WHO 2016,  Day 2006,  du Châtelet et al. 2019 [unpublished],  Bergh et al. 2011,  WHO 2014a,  WHO 2014e,  WHO 2014d,  WHO 2014c,  Bayley et al. 2015,  Bakker et al. 2011,1  Nyamtema et al. 2010,  Muffler et al. 2007, | A number of studies reported that MPDSR addressed resolved critical gaps in quality of care (Abebe et al. 2017, Agaro et al. 2016, Armstrong et al. 2014, Bandali et al. 2016, Dartey 2012, Hofman and Mohammed 2014, Kerber et al. 2015, Magoma et al. 2015, MCSP 2017a, Mutsigiri-Murewanhema et al. 2017, Nyamtema et al. 2010, Rhoda et al. 2014, WHO 2016, Day 2006, du Châtelet et al. 2019 [unpublished], WHO 2014a, WHO 2014e, WHO 2014d, WHO 2014c, Bakker et al. 2011, Bayley et al. 2015, Belizan et al. 2011, Biswas et al. 2015, Ethiopia Federal Ministry of Health et al. 2016, Lewis 2014b, MCSP 2017b, MCSP 2018, Muffler et al. 2007, Bergh et al. 2011). Though few of these studies documented these changes with evidence beyond perceptions of those interviewed or the authors (Abebe et al. 2017, Bandali et al. 2016, Nyamtema et al. 2011, Day 2006, du Châtelet et al. 2019 [unpublished], WHO 2014a, WHO 2014b, Belizan et al. 2011, Bergh et al. 2011, MCSP 2018). Two studies (one report; one article) from Ethiopia observed that once MDSR started, the level of documentation improved resulting in better communication and organized care, ultimately leading to more buy-in by stakeholders in the process (Abebe et al. 2017, Ethiopian Public Health Institute et al. 2017).  In the various studies, stakeholders perceived that MPDSR led to some changes that addressed critical gaps in quality of care. The actions included addressing gaps in supplies like blood and other equipment (Bandali et al. 2016, Lewis 2014b, Day 2006, du Châtelet et al. 2019 [unpublished],), addressing human resource shortages and capacity (Day 2006, Ethiopian Public Health Institute et al. 2017, Agaro et al. 2016), improving referral and emergency transport (Lewis 2014b, Nyamtema et al. 2011, WHO 2014e, WHO 2016), data utilization and thus better district planning (Muffler et al. 2007, Biswas 2017). While these changes were reported, there was little evidence documenting these changes and/or if they were sustainable. |
|  | Framing - Relative advantage | None | No studies described relative advantage |
|  | Trialability | Bandali et al. 2016,  Biswas et al. 2015,  Hofman and Mohammed 2014,  Lewis 2014b,  MCSP 2017a,  MCSP 2018,  Rhoda et al. 2014,  WHO 2016,  Day 2006,  WHO 2014a,  WHO 2014e,  WHO 2014c,  Bayley et al. 2015,  Dumont et al. 2009,  Ethiopia Federal Ministry of Health et al. 2016,  Kongnyuy and van den Broek 2008,  Richard et al. 2009,  WHO 2014b,  Dumont et al. 2013,  Hadush et al. 2016,  Grellier and Shome 2011  Nam 2011 | The literature reflects the WHO recommendation for a phased approach of implementing MPDSR, with many studies reporting on small scale implementation efforts (Abebe et al. 2017, Day 2006, De Brouwere et al. 2014, Hadush et al. 2016, WHO 2014b, WHO 2016, Ethiopia Federal Ministry of Health et al. 2016, WHO 2014e, WHO 2014c, Dumont et al. 2009, Pattinson et al. 2009, Rhoda et al. 2014, Belizan et al. 2011, Biswas et al. 2015, Bayley et al. 2015, Lewis 2014b, Hofman and Mohammed 2014, Hussein et al. 2016, Kongnyuy and van den Broek 2008, Lewis 2014a, Kongnyuy and van den Broek 2009, MCSP 2017a, MCSP 2018, Koblinsky 2017) There were nine studies identified specifically as pilots, most conducted at the facility level (Dumont et al. 2009, Richard et al. 2009, Biswas et al. 2015, Day 2006, Nam 2011, Hofman and Mohammed 2014, Nyamtema et al. 2011, Bayley et al. 2015,) and one at subnational level (Bandali et al. 2016,). All but one of the studies involved the Ministry of Health in partnership with support from a development partner or foreign academic institution.  Approaches and results varied across these pilots. Some demonstrated that with initial external support to start the process, facility-based maternal and/or perinatal death audit can be implemented in resource limited settings, with positive acceptance from health staff (Biswas et al. 2015, Hofman and Mohammed 2014, Nyamtema et al. 2011, Day 2006). In Malawi, a pilot study tested “community-linked maternal death review” and found positive results with increased death notifications, richer data, greater stakeholder engagement and more actions taken (Bayley et al. 2015). In Nigeria, Evidence4Action pilot tested a scorecard with State Ministries of Health to track MDSR progress and found the process to be an effective accountability tool (Bandali et al. 2016). Local leadership was noted as a critical enabling factor across several studies (Hofman and Mohammed 2014, Dumont et al. 2009, Richard et al. 2009).  Other pilots demonstrated that the death audit process can be destabilizing or even threatening, especially in settings where staff are not used to self-evaluation and critical review (Dumont et al. 2009, Richard et al. 2009). The study from Burkina Faso even took efforts to prevent potential negative experiences by introducing and initiating the facility-based death review process first with midwives to familiarize them with the process and strengthen their confidence and ownership. Yet the endline evaluation found that 50% of the midwives reported the process as repressive by those in charge (Richard et al. 2009).  The challenge of sustained implementation beyond projects was recognized in several studies, not just the pilots, and there was no reporting of modifications or expansion after these pilots (Hofman and Mohammed 2014, Muffler et al. 2007, Grellier and Shome 2011, Nam 2011, WHO 2014e). For example, the Saving Mothers and Newborns project of FIGO, which had some success in introducing and strengthening maternal and perinatal death audits across ten countries (Kenya, Nigeria, Uganda, Kosovo, Moldova, Ukraine, Haiti, Peru, Uruguay, and Pakistan) (Grellier and Shome 2011), failed to see sustainable practice after the project ended (Nam 2011). One record noted that coordination declined at the end of the project (WHO 2014e).  Half of the reviews and commentaries (7/14) discuss the importance of pilots and/or the phased approach when implementing MPDSR (Biswas 2017, De Brouwere et al. 2014, Hussein et al. 2016, Koblinsky 2017, Kongnyuy and van den Broek 2009, Lewis 2014a, Pattinson et al. 2009). A commentary reflecting on the FIGO LOGIC project in Cameroon indicated the tools and trainers were in place to move from pilot to expansion, but there were no funds for training and coaching which were key factors identified as enablers (De Brouwere et al. 2014). |
|  | Adaptability | Lewis 2014b,  Rhoda et al. 2014,  WHO 2014e,  WHO 2014b,  Abebe et al. 2017,  Bakker et al. 2011,  Belizan et al. 2011,  Kerber et al. 2015,  Bergh et al. 2011,  Muffler et al. 2007,  Balogun and Musoke 2014,  Congo et al. 2017,  Smith et al. 2017c,  de Kok et al. 2017,  Smith et al. 2017b, | Studies that reflected adaptability around implementation of MPDSR recognized the notion of “different sites, different modalities” (Belizan et al. 2011) whereby the process varied between facilities, subnational and national levels (Belizan et al. 2011, Congo et al. 2017, de Kok et al. 2017, WHO 2014b, Smith et al. 2017c, Scott and Danel 2016, WHO 2014e). Variations included different drivers of the process e.g. facility manager, head of department, midwife, clinical outreach person, etc; nature of review meetings e.g. frequency, standalone vs integrated, format; and composition of participants.  Overtime, processes have adapted and changed in a number of settings (Muffler et al. 2007, Pearson et al. 2009, Belizan et al. 2011, Rhoda et al. 2014) such as a shift in culture from a blame to a learning environment due to continuous and improved practice of audits overtime (Bakker et al. 2011).  Examples:   - Ethiopia’s process encountered a change in overall management when MDSR became the responsibility of the Public Health Emergency Management directorate activities rather than the Ministry of Health’s Maternal and Child Health directorate after only one year of implementation, creating delays and confusion between staff on roles and responsibilities (Abebe et al. 2017). - A South African study reported that facilities determined the key role players or drivers, therefore the individuals responsible varied (Belizan et al. 2011, Bergh et al. 2011). - A study from Nigeria compared different MDR facility-level meetings showing that the process and approach can slightly vary due to different role players (de Kok et al. 2017). - Subnational variation, including oversight and surveillance, (Rhoda et al. 2014, WHO 2014b, Congo et al. 2017) as well as national variations in processes (WHO 2014e, Smith et al. 2017c) were also described. - A study from Burkina Faso reported the presentation of findings from the audit meetings took place across all health districts but how they presented (method and frequency) varied (Congo et al. 2017).   While variability between processes across facilities assumes local adaption of the intervention, we did not find any studies that identified which elements were core verses peripheral to change.  The lack of the overall system being able to adapt to the local needs identified through the review process prevented uptake of MDR in the South Sudan study (Balogun and Musoke 2014). |
| Domain 2: Individual | Technical skills & Knowledge | Rhoda et al. 2014,  WHO 2014e,  Abebe et al. 2017,  Bakker et al. 2011,  Belizan et al. 2011,  Kerber et al. 2015,  Bergh et al. 2011,  Muffler et al. 2007,  Balogun and Musoke 2014,  Congo et al. 2017,  Smith et al. 2017c,  Smith et al. 2017b,  Biswas et al. 2015,  MCSP 2017a,  MCSP 2018,  Day 2006,  WHO 2014c,  Dumont et al. 2009,  Ethiopia Federal Ministry of Health et al. 2016,  Kongnyuy and van den Broek 2008,  Richard et al. 2009,  Agaro et al. 2016,  Armstrong et al. 2014,  Dartey 2012,  MCSP 2017b,  Mutsigiri-Murewanhema et al. 2017,  du Châtelet et al. 2019 [unpublished],  Ministry of Health and Sanitation [Sierra Leone] 2017,  Purandare et al. 2014,  Tapesana et al. 2017,  van Hamersveld et al. 2012, | Many records reflected on technical skills and knowledge of facility staff to complete MPDSR processes (Agaro et al. 2016, Armstrong et al. 2014, Bakker et al. 2011, Belizan et al. 2011, Rhoda et al. 2014, Dartey 2012, Kerber et al. 2015, Kongnyuy and van den Broek 2008, Lewis 2014a, MCSP 2017b, MCSP 2017a, MCSP 2018, Congo et al. 2017, Purandare et al. 2014, Smith et al. 2017b, Smith et al. 2017c, Tapesana et al. 2017, van Hamersveld et al. 2012, WHO 2014e). Critical skills required for MPDSR include data entry and analysis as well as ability to facilitate meaningful and blame-free discussions during audit meetings (Kerber et al. 2015). Some studies report that people had the skills necessary to complete the audit process (Rhoda et al. 2014, Belizan et al. 2011, Dartey 2012, Bakker et al. 2011); other studies report mixed results among respondents (Tapesana et al. 2017, Day 2006, WHO 2014c); and many studies identified the lack of skills overall as a barrier by multiple studies (Agaro et al. 2016, Congo et al. 2017, Kongnyuy and van den Broek 2009, Hussein et al. 2016, MCSP 2018, Balogun and Musoke 2014, Kongnyuy and van den Broek 2008, Mutsigiri-Murewanhema et al. 2017).  Specific skills gaps included data entry or poor record keeping (Biswas et al. 2015, Ministry of Health and Sanitation [Sierra Leone] 2017, Richard et al. 2009, Balogun and Musoke 2014, Kongnyuy and van den Broek 2008, Purandare et al. 2014, Biswas et al. 2015, Ethiopia Federal Ministry of Health et al. 2016, Dumont et al. 2009, Ministry of Health and Sanitation [Sierra Leone] 2017,), poor identification (including cause of death analysis) and reporting of deaths (WHO 2014e, Armstrong et al. 2014, Ministry of Health and Sanitation [Sierra Leone] 2017, Dumont et al. 2009, MCSP 2017b, MCSP 2017a, MCSP 2018,), limited use of data for routine analysis (Armstrong et al. 2014, MCSP 2017a), and overall unfamiliarity with the audit process (van Hamersveld et al. 2012, Congo et al. 2017, Agaro et al. 2016, Kongnyuy and van den Broek 2008, Smith et al. 2017b, Smith et al. 2017c). One study noted the challenge of finding people who are able to know how to run these meetings effectively (Smith et al. 2017b,). The lack of skills overall were identified as a barrier by multiple studies (Agaro et al. 2016, Congo et al. 2017, Kongnyuy and van den Broek 2009, Hussein et al. 2016, MCSP 2018, Balogun and Musoke 2014, Kongnyuy and van den Broek 2008, Mutsigiri-Murewanhema et al. 2017) and confirmed by a previous review (Raven et al. 2011). Most records made broad based statements around lack of skills; though some linked the lack of knowledge on the reporting process to poor compliance and incomplete reporting through the system (Smith et al. 2017c). Few studies actually assessed the level of knowledge (Tapesana et al. 2017, Day 2006,van Hamersveld et al. 2012, Richard et al. 2009), and these studies used different methods and questions to assess technical skills and knowledge of individuals.  Skills development takes time and goes beyond one training session. One study from South Africa found that: *“It took one day of training but on average 3–6 months before management understood the value of PPIP and up to 3 years before staff members fully appreciated the full benefit that PPIP provided to a facility.”(Rhoda et al. 2014)* |
|  | Self-efficacy | Abebe et al. 2017,  Belizan et al. 2011,  Muffler et al. 2007,  Balogun and Musoke 2014,  Richard et al. 2009,  Armstrong et al. 2014,  Tapesana et al. 2017,  van Hamersveld et al. 2012, | Individual confidence to implement MPDSR was explored in a few studies (Belizan et al. 2011, Richard et al. 2009, Muffler et al. 2007, Armstrong et al. 2014, Abebe et al. 2017, Tapesana et al. 2017, Balogun and Musoke 2014, van Hamersveld et al. 2012). In some cases, staff were confident to implement with supportive supervision, appropriate tools and oversight from management or health specialists (Belizan et al. 2011, Richard et al. 2009, Muffler et al. 2007, Armstrong et al. 2014); whereas other studies showed mixed levels of confidence among study participants (Abebe et al. 2017, Tapesana et al. 2017,) or even that the lack of confidence made it difficult to implement the intervention (Balogun and Musoke 2014, van Hamersveld et al. 2012). |
|  | Individual motivation | Abebe et al. 2017,  Balogun and Musoke 2014,  Biswas et al. 2015,  Dartey 2012,  Dumont et al. 2009,  Ethiopia Federal Ministry of Health et al. 2016,  Karamagi et al. 2018,  Kerber et al. 2015,  Kongnyuy and van den Broek 2008,  Manandhar 2004,  MCSP 2017c,  MCSP 2017a,  Mutsigiri-Murewanhema et al. 2017,  Purandare et al. 2014,  Smith et al. 2017b,  Smith et al. 2017c,  van Hamersveld et al. 2012,  WHO 2013,  Day 2006,  Richard et al. 2009,  Bergh et al. 2011,  WHO 2014b,  WHO 2014e, | Studies that examined individual motivation mostly identified extrinsic factors, such as seeking to improve quality of care (WHO 2014e, Muffler et al. 2007, WHO 2014c, Belizan et al. 2011), adhering to expectation from sub-national teams (Kongnyuy and van den Broek 2008, Belizan et al. 2011), gaining skills or knowledge (van Hamersveld et al. 2012, Bakker et al. 2011, Armstrong et al. 2014,), or incentives (WHO 2014c). Positive outcomes from the MPDSR process also motivated health workers (Abebe et al. 2017, Bakker et al. 2011, Belizan et al. 2011, Biswas et al. 2015, WHO 2014e). The lack of resources to support M/PDSR processes as well as the non-action of MPDSR related recommendations were specifically cited as a de-motivating factor for staff (Agaro et al. 2016, MCSP 2017c, Richard et al. 2009, Kongnyuy and van den Broek 2008, Muffler et al. 2007). The hierarchical nature of meetings also appear to link to motivation of personnel to participate in the process (Dumont et al. 2009, Kerber et al. 2015, van Hamersveld et al. 2012,). The literature also revealed that some perceive the process as time consuming and arduous, resulting in inefficiencies in the process and lack of commitment to implement (Smith et al. 2017b, Armstrong et al. 2014, de Kok et al. 2017).  A number of studies reflected that individuals found the MPDSR process to be helpful, especially for learning (WHO 2014e, Abebe et al. 2017, Bakker et al. 2011, Biswas et al. 2015, Congo et al. 2017, WHO 2014c, Belizan et al. 2011, van Hamersveld et al. 2012, du Châtelet et al. 2019 [unpublished], Dartey 2012, MCSP 2017b, Richard et al. 2009, Bergh et al. 2011).  *“health workers perceived audit … not merely [as] a system of evaluation of care with the objective to implement system improvements, but more importantly [as] a direct learning opportunity.” (Bakker et al. 2011)*  More intrinsic motivation was reflected in some studies that found individual appreciation of the MPDSR as enabling self-reflection and learning around self-improvement (Dartey 2012, Lewis 2014b, Bakker et al. 2011, Armstrong et al. 2014, van Hamersveld et al. 2012). MPDSR may be linked to professionalism of maternity care itself (Belizan et al. 2011, Biswas et al. 2015, Richard et al. 2009). Lewis’ commentary highlights the importance of ‘continual moral pressure’ on successful implementation, which calls for individual level consciousness for self-improvement in order to improve health care services (Lewis 2014a). One study demonstrated the underlying value of life when they acknowledged, “one minute silence for dead babies and mothers in [a] meeting” (Day 2006). We found that some literature recognized that individual motivation and ownership of MPDSR may also be influenced by inner setting elements, such as culture (de Kok et al. 2017) and team structures (Dumont et al. 2009, Kerber et al. 2015).  In enabling environments, individuals were motivated, enthusiastic, engaged (du Châtelet et al. 2019 [unpublished], Bakker et al. 2011) and acknowledged the individual benefits of implementing MPDSR, such as career development, increased cohesiveness, increased cooperation and professional accountability among others (de Kok et al. 2017, Dartey 2012, MCSP 2017b). Despite appreciation of the system wide related goals of implementing MPDSR, much of the emphasis was on the direct/personal benefits accrued from implementing MPDSR (Bakker et al. 2011). This calls for further efforts to sensitize actors about the linkages between the individual and system wide benefits of implementing MPDSR.  A study from Burkina Faso demonstrates how individual motivation is complex as motives are not always clear: *"They [midwives] rated it as the principal promoter of change in their working practice after morning staff meetings, training sessions and clinical guidelines. It is not always clear if they changed their practice with the conscience that it will improve management of the woman or only by fear of blame during the audit session (Richard et al. 2009)”*  One commentary noted that *“The success of audit largely depends on the motivation of the healthcare providers themselves. If they are able to evaluate the care they are giving, and willing and able to give praise where this is due, as well as make amendments where needed, then this should lead to improved motivation, ownership and sense of responsibility for delivering good quality care.”(Kongnyuy and van den Broek 2009)* |
|  | Individual identification with intervention | Biswas et al. 2015,  Dumont et al. 2009,  Ethiopia Federal Ministry of Health et al. 2016,  Kerber et al. 2015,  MCSP 2017c,  MCSP 2017a,  Purandare et al. 2014,  Smith et al. 2017b,  Smith et al. 2017c,  van Hamersveld et al. 2012,  WHO 2013,  Day 2006,  Richard et al. 2009,  Ethiopian Public Health Institute et al. 2017  Armstrong et al. 2014,  Rhoda et al. 2014,  Tapesana et al. 2017,  Grellier and Shome 2011 | The importance of ownership of and commitment to the intervention was noted by a number of studies (Abebe et al. 2017, Bandali et al. 2016, Hadush et al. 2016, Lewis 2014b, Richard et al. 2009, WHO 2014e, WHO 2014c, Belizan et al. 2011, de Kok et al. 2017, Dartey 2012, Armstrong et al. 2014, Biswas et al. 2015, Kerber et al. 2015, E4A 2012, Bergh et al. 2011). Two records reflected on the importance of maintaining and balancing external and internal stakeholder confidence and commitment to the process (Hadush et al. 2016, E4A 2012). Ensuring local buy in or ownership was identified as an enabler (3, Belizan et al. 2011). Health workers who are committed to their jobs and to quality improvement were found more willing to adapt to change and accept the MPDSR (Lewis 2014b, Richard et al. 2009, WHO 2014e, Belizan et al. 2011, Bergh et al. 2011). As supported by a multi-country report from the South-East Asia Region: *“The commitment of physicians and supervisors is found to be a strength of the system; they have been encouraged by the fact that recommendations made at the audit meeting have been used as inputs for district planning, and have resulted in tangible improvements in the health system.” (WHO 2014e)*  Ownership of the intervention though may come over time as people see the benefits of change (Belizan et al. 2011, Richard et al. 2009, Day 2006, Bergh et al. 2011). One study from Ethiopia mentioned a shift in individual willingness to complete case notes accurately since it was seen as having a useful purpose with MDSR rather than being an additional burden (Abebe et al. 2017).  Lack of ownership prevents effective implementation (van Hamersveld et al. 2012, Dumont et al. 2009, de Kok et al. 2017, Muffler et al. 2007, Mogobe et al. 2007). For example, a study from Nigeria found that the lack of personal accountability for an honest process resulted in shifting responsibility or "Passing the buck" to other staff. (de Kok et al. 2017). This quote reflects the consequence when there is no ownership or commitment to the intervention:  *"Performing clinical audits demands that, health care providers engage in critical self evaluation irrespective of their position. Staff had difficulty in accepting professional responsibility and clinicians in willing to change practices and habits. If hierarchy and poor team spirit resulted in inflexibility, there was little chance of realizing the first aim of clinical audit, namely, to promote change. When curiosity and willingness to change were dominated by fears of being held responsible for errors or of having one’s professional competence questioned, deficiencies in the quality of care could not be identified and audits failed." Muffler et al. 2007,*  Another quote: *“There is little experience from other countries on incentives and rewards for those involved in reporting a maternal death or in the MDR, but there is anecdotal evidence that health professionals participating in MDRs have a sense of professional satisfaction and pride.” WHO 2014c* |
|  | Individual orientation to collaboration | None | The scoping review found that little is known about how individual leadership traits and critical thinking or problem solving skills support implementation.  One commentary described this professionalism as a set of values that includes integrity, compassion, altruism, continuous improvement, excellence and working in partnership with members of the wider healthcare team (Lewis 2014a). |
| Domain 3: Inner setting | Readiness for Implementation | Biswas et al. 2015,  Dumont et al. 2009,  Ethiopia Federal Ministry of Health et al. 2016,  Kerber et al. 2015,  MCSP 2017c,  MCSP 2017a,  Purandare et al. 2014,  Smith et al. 2017c,  WHO 2013,  Day 2006,  Richard et al. 2009,  Ethiopian Public Health Institute et al. 2017  Armstrong et al. 2014,  Rhoda et al. 2014,  Tapesana et al. 2017,  Abebe et al. 2017,  Balogun and Musoke 2014,  Dartey 2012,  Karamagi et al. 2018,  Kongnyuy and van den Broek 2008,  Manandhar 2004,  Mutsigiri-Murewanhema et al. 2017,  Bergh et al. 2011,  WHO 2014b,  WHO 2014e,  Hofman and Mohammed 2014,  Agaro et al. 2016,  Bakker et al. 2011,  Bandali et al. 2016,  Belizan et al. 2011,  Congo et al. 2017,  Ministry of Health and Sanitation [Sierra Leone] 2017,  Hadush et al. 2016,  Lewis 2014b,  Magoma et al. 2015  MCSP 2017b,  MCSP 2018,  Mogobe et al. 2007,  Nyamtema et al. 2011,  Nyamtema et al. 2010,  WHO 2016,  Koblinsky et al. 2017,  du Châtelet et al. 2019 [unpublished],  Muffler et al. 2007,  E4A 2012,  WHO 2014a,  WHO 2014d,  WHO 2014c, | The formation and or existence of MPDSR committees greatly facilitated the implementation of MPDSR (Abebe et al. 2017, Agaro et al. 2016, Bakker et al. 2011, Bandali et al. 2016, Rhoda et al. 2014, Tapesana et al. 2017, Koblinsky et al. 2017, Day 2006, MCSP 2017c, du Châtelet et al. 2019 [unpublished], Ministry of Health and Sanitation [Sierra Leone] 2017, Ethiopia Federal Ministry of Health et al. 2016, Magoma et al. 2015, Nyamtema et al. 2011, Dartey 2012, WHO 2014b, WHO 2014c, Hadush et al. 2016, Kerber et al. 2015), especially when functional at multiple levels (WHO 2014a, WHO 2014d, Karamagi et al. 2018, MCSP 2017c, MCSP 2017b, MCSP 2018). At the national level, MPDSR committees were noted in a number of studies (Koblinsky et al. 2017, du Châtelet et al. 2019 [unpublished], Mogobe et al. 2007, Ethiopian Public Health Institute et al. 2017 MCSP 2017b, Manandhar 2004, WHO 2016, WHO 2014a, WHO 2014d, Karamagi et al. 2018). In most cases, there were clear organizational and managerial arrangements for the national MPDSR committees (Koblinsky et al. 2017,953, MCSP 2017b, Mogobe et al. 2007, Ethiopian Public Health Institute et al. 2017 Manandhar 2004). Yet, in some countries, national committees lack clear structure and appeared ineffective, such as in Sierra Leone (du Châtelet et al. 2019 [unpublished],). A number of studies mention sub-national committees as an enabler for implementation (Hofman and Mohammed 2014, Nyamtema et al. 2010, 953, MCSP 2017c, MCSP 2017b, MCSP 2018, WHO 2013, WHO 2014a, WHO 2014d, Karamagi et al. 2018); though this may not mean implementation at facility level, especially in settings where the intervention is still being introduced (MCSP 2017a,). A few studies highlighted the existence of MPDSR committees at community (WHO 2014a, WHO 2014b, Bayley et al. 2015).  A designated focal person enables implementation, though the assigned individuals ranged from facility manager, to a designed midwife, to a regional specialist (Abebe et al. 2017, Muffler et al. 2007, WHO 2014b, WHO 2014d, Belizan et al. 2011, Smith et al. 2017c, Ministry of Health and Sanitation [Sierra Leone] 2017,WHO 2016, Richard et al. 2009, Bakker et al. 2011, MCSP 2017c, MCSP 2017b, Rhoda et al. 2014, Kerber et al. 2015). The lack of a focal person was identified as a barrier towards implementation (Mogobe et al. 2007, Ministry of Health and Sanitation [Sierra Leone] 2017,Balogun and Musoke 2014). Other tangible inputs towards implementation are mentioned in the literature, such as regularly scheduled meetings (WHO 2014e, Hadush et al. 2016, Bandali et al. 2016, Belizan et al. 2011, Dumont et al. 2009, MCSP 2017c, Dartey 2012) or establishing “audit charters” (e.g. agreed codes of ethics to follow during audit process) (Congo et al. 2017, Richard et al. 2009, MCSP 2017c); but not investigated in detail as an enabler. Available tools and appropriate forms for MPDSR was a recognized input in many records (Koblinsky et al. 2017, Balogun and Musoke 2014, Bandali et al. 2016, Tapesana et al. 2017,MCSP 2017b, WHO 2014a, WHO 2014e, WHO 2014c, Kongnyuy and van den Broek 2008, Ministry of Health and Sanitation [Sierra Leone] 2017, WHO 2016, Dumont et al. 2009, Nyamtema et al. 2011, Richard et al. 2009, Dartey 2012, Biswas et al. 2015, MCSP 2017c, MCSP 2017b, Purandare et al. 2014, Belizan et al. 2011); with the lack of tools noted as a barrier in other studies (Abebe et al. 2017, Dumont et al. 2009, 3, Hofman and Mohammed 2014, MCSP 2017a, MCSP 2018).  The importance of training on MPDSR processes at national, subnational and facility levels was highlighted in a number of studies (Abebe et al. 2017, Armstrong et al. 2014, MCSP 2017b, MCSP 2017c, Belizan et al. 2011, WHO 2014c, Smith et al. 2017c, Ethiopia Federal Ministry of Health et al. 2016, WHO 2016, Dumont et al. 2009, 301, Nyamtema et al. 2011,), ranging from once-off trainings to committee members or data captures (Belizan et al. 2011) to refresher or on-going trainings and supportive supervision (WHO 2016, Belizan et al. 2011, Rhoda et al. 2014,) to trainers of trainers (Ethiopia Federal Ministry of Health et al. 2016) to pre-service trainings (Armstrong et al. 2014). The lack of training was identified as an inhibitor in some studies (Ministry of Health and Sanitation [Sierra Leone] 2017, van Hamersveld et al. 2012, WHO 2014e, Nyamtema et al. 2010, Mutsigiri-Murewanhema et al. 2017, Balogun and Musoke 2014). Several records identified health management inputs that hinder effective implementation of MPDSR including shortage and capacity of health workers (Balogun and Musoke 2014, Bakker et al. 2011, WHO 2014e, WHO 2014c, Nyamtema et al. 2011, Richard et al. 2009, E4A 2012, Mutsigiri-Murewanhema et al. 2017, Biswas et al. 2015, MCSP 2017b, Dartey 2012, Congo et al. 2017, Agaro et al. 2016, Mutsigiri-Murewanhema et al. 2017, Nyamtema et al. 2010, van Hamersveld et al. 2012,) and disengaged leadership and inadequate management capacity (3, Dumont et al. 2009, Smith et al. 2017a, Hofman and Mohammed 2014, de Kok et al. 2017, van Hamersveld et al. 2012, WHO 2014d, WHO 2014b, du Châtelet et al. 2019 [unpublished], Koblinsky et al. 2017, MCSP 2018, Pattinson et al. 2005, Kerber et al. 2015, Balogun and Musoke 2014, Mutsigiri-Murewanhema et al. 2017, van Hamersveld et al. 2012, Nyamtema et al. 2010, Balogun and Musoke 2014). |
|  | Team composition and characteristics | Biswas et al. 2015,  Dumont et al. 2009,  Kerber et al. 2015,  MCSP 2017c,  MCSP 2017a,  Day 2006,  Richard et al. 2009,  Armstrong et al. 2014,  Rhoda et al. 2014,  Dartey 2012,  Karamagi et al. 2018,  Kongnyuy and van den Broek 2008,  Mutsigiri-Murewanhema et al. 2017,  WHO 2014b,  WHO 2014e,  Hofman and Mohammed 2014,  Bakker et al. 2011,  Bandali et al. 2016,  Congo et al. 2017,  Ministry of Health and Sanitation [Sierra Leone] 2017,  Lewis 2014b,  MCSP 2017b,  MCSP 2018,  Mogobe et al. 2007,  Nyamtema et al. 2011,  Nyamtema et al. 2010,  Koblinsky et al. 2017,  Muffler et al. 2007,  WHO 2014d,  WHO 2014c,  Smith et al. 2017b,  van Hamersveld et al. 2012,  Grellier and Shome 2011  Bayley et al. 2015,  de Kok et al. 2017,  Pearson et al. 2009, | Establishing a multidisciplinary audit committee, including doctors, midwives, nurses and managers, is listed as a step in the WHO guidelines and hence national guidelines. As such, many records described the MPDSR committees/teams as being multidisciplinary comprising various cadres of health workers at facility level and external stakeholders from ministries of health and MPDSR implementing partners at subnational and national levels (Armstrong et al. 2014, Bandali et al. 2016, Rhoda et al. 2014, Muffler et al. 2007, Koblinsky et al. 2017, Day 2006, Grellier and Shome 2011, MCSP 2017c, MCSP 2017b, MCSP 2018, WHO 2014b, WHO 2014e, WHO 2014d, WHO 2014c, Lewis 2014b, Smith et al. 2017b, de Kok et al. 2017, Hofman and Mohammed 2014, Mutsigiri-Murewanhema et al. 2017, Nyamtema et al. 2011, Nyamtema et al. 2010, van Hamersveld et al. 2012, Richard et al. 2009, Dartey 2012, Karamagi et al. 2018, Bakker et al. 2011, Biswas et al. 2015, Congo et al. 2017, Bayley et al. 2015, Mogobe et al. 2007, Ministry of Health and Sanitation [Sierra Leone] 2017). Many studies recorded the number of members in review committees; which varied depending on level and size of facility (Bakker et al. 2011, du Châtelet et al. 2019 [unpublished],Nyamtema et al. 2010, Bandali et al. 2016, Koblinsky et al. 2017, WHO 2014e, WHO 2014b, Lewis 2014a, MCSP 2017b, MCSP 2017a, MCSP 2017c, Koblinsky 2017, MCSP 2018, Hofman and Mohammed 2014, Kerber et al. 2015, Mogobe et al. 2007, Biswas et al. 2015, Dartey 2012). The composition of teams are an important component of implementation in order to ensure multiple perspectives are considered and shared learning occurs. Restricted participation was identified as a barrier (Congo et al. 2017, Dumont et al. 2009, de Kok et al. 2017, Bayley et al. 2015) especially when there is low participation of nurses (Bakker et al. 2011, Dumont et al. 2009) and support staff such as transport officers and pharmacist (Bakker et al. 2011) as well as management (van Hamersveld et al. 2012,) and heads of departments (Dumont et al. 2009). One pilot study started implementation only with the midwives and auxiliary midwives only in order to establish a culture of evaluation in a blame-free setting and only broadened membership after they were comfortable with the practice to include other hospital personnel, such as medical doctors, laboratory technicians, pharmacist and administrators (Richard et al. 2009). Challenges around creating multi-disciplinary teams included high staff turnover or competing priorities (MCSP 2017a, Kongnyuy and van den Broek 2008, Pearson et al. 2009), lack of interest by staff (van Hamersveld et al. 2012), and hierarchical nature of meetings (Dumont et al. 2009). One study noted that the multidisciplinary nature of these teams led to tensions among the teams; thereby affecting the implementation process (MCSP 2017a) |
|  | Organizational incentives & rewards (or disincentives/sanctions) | Dumont et al. 2009,  Richard et al. 2009,  Bakker et al. 2011,  MCSP 2017b,  MCSP 2018,  Muffler et al. 2007,  WHO 2014c,  Abebe et al. 2017,  Balogun and Musoke 2014,  Agaro et al. 2016,  du Châtelet et al. 2019 [unpublished], | The provision of incentives was noted in a number of studies, including refreshments (Dumont et al. 2009,), extra training (Armstrong et al. 2014, WHO 2014c, Ethiopia Federal Ministry of Health et al. 2016, MCSP 2017b, Hofman and Mohammed 2014, Nyamtema et al. 2011, Ministry of Health and Sanitation [Sierra Leone] 2017, Balogun and Musoke 2014, WHO 2014e, Mutsigiri-Murewanhema et al. 2017, Hussein et al. 2016, Agaro et al. 2016, van Hamersveld et al. 2012), financial motivation, such as per diems, and transport (Dumont et al. 2009, Balogun and Musoke 2014, Bakker et al. 2011, MCSP 2017b, MCSP 2018, Richard et al. 2009). A number of records identified the lack of such incentives as a barrier (Dumont et al. 2009, Balogun and Musoke 2014, Bakker et al. 2011, MCSP 2017b, MCSP 2018, van Hamersveld et al. 2012, Agaro et al. 2016, Congo et al. 2017, Hussein et al. 2016). One study reported that facility staff felt there was no need for financial compensation (Richard et al. 2009). Even though we did not find any studies that systematically examined the impact of these incentives, a number of studies included organizational incentives as recommendations for strengthening implementation, even just the provision of food or tea (Agaro et al. 2016, MCSP 2017c). Some studies recognized the negative consequences of incentives when projects are terminated, resulting in demotivation (Agaro et al. 2016, van Hamersveld et al. 2012). There were no studies that examined the use of sanctions for lack of implementation. |
|  | Team relationship | Dumont et al. 2009,  Richard et al. 2009,  Bakker et al. 2011,  MCSP 2017b,  Muffler et al. 2007,  WHO 2014c,  Abebe et al. 2017,  Kerber et al. 2015,  MCSP 2017a,  Dartey 2012,  WHO 2014e,  Congo et al. 2017,  WHO 2014d,  van Hamersveld et al. 2012,  de Kok et al. 2017,  Ethiopia Federal Ministry of Health et al. 2016,  Purandare et al. 2014,  Bergh et al. 2011,  Belizan et al. 2011, | MPDSR implementation positively and negatively affected and is affected by the nature of communication, collaboration and networking within and across teams and among stakeholders involved in the implementation process. Most studies that reported on the impact of communication on MPDSR implementation focused on the relationships among health facility staff and surrounding clinics (Congo et al. 2017, van Hamersveld et al. 2012, Agaro et al. 2016, Hofman and Mohammed 2014, MCSP 2017b, MCSP 2018, Muffler et al. 2007, Purandare et al. 2014). The positive effects of communication and collaboration include engagement and considered opinions of various stakeholders including local experts, different cadres of staff, as well as communities. The MPDSR process itself was found to nurture team spirit and collaboration (WHO 2014d, Purandare et al. 2014). For example, “sharing regular updates on the program’s progress ensured timely help and kept the team motivated to deliver high-level performance.”(Purandare et al. 2014) In contexts where a teamwork approach to implementing MPDSR was adopted, there was consensus, inclusiveness, delegation of responsibility and continuity of the MPDSR implementation processes (MCSP 2018,du Châtelet et al. 2019 [unpublished],MCSP 2017b,Agaro et al. 2016,Hofman and Mohammed 2014,Dartey 2012, Muffler et al. 2007). These benefits accrued teams that invested deliberate efforts and strategies such as mentorship, adherence to guidelines, protocols as well as upholding certain norms and values to nurture a conducive atmosphere (Agaro et al. 2016,Hussein et al. 2016, MCSP 2018,du Châtelet et al. 2019 [unpublished],de Kok et al. 2017,Lewis 2014b,van Hamersveld et al. 2012). Strong communication, involvement and support from hospital management was also found to strengthen team relationships (Belizan et al. 2011, Muffler et al. 2007, Dartey 2012,). The lack of communication and coordination across teams, including poorly functioning teams, came across as a formidable barrier in other records (Congo et al. 2017, Dumont et al. 2009, Balogun and Musoke 2014, WHO 2014c, MCSP 2017a, Ethiopia Federal Ministry of Health et al. 2016).  The existence of hierarchies within teams and across various contexts was reported to have positively influenced team relationships through provision of leadership and mentorship (Dumont et al. 2009, Bakker et al. 2011, de Kok et al. 2017). Although more records showed the negative influence of professional hierarchies between health cadres, notably the silencing of the more junior staff and nurses in the process (Kerber et al. 2015, 959, de Kok et al. 2017, Dumont et al. 2009, Richard et al. 2009, Muffler et al. 2007, Armstrong et al. 2014). Structural hierarchies also constrained the performance of teams in cases where the senior members were absent or unable to perform their duties (van Hamersveld et al. 2012, Hofman and Mohammed 2014, de Kok et al. 2017, Abebe et al. 2017). Not many studies have examined the effect of hierarchies and teamwork on implementation of MPDSR beyond the health facility level. The few that have attempted only described the institutional reporting structures rather than the inner team dynamic (MCSP 2017c, MCSP 2017b, MCSP 2017a, MCSP 2018). |
|  | Implementation culture and climate | Richard et al. 2009,  Bakker et al. 2011,  MCSP 2017b,  Muffler et al. 2007,  Abebe et al. 2017,  Kerber et al. 2015,  MCSP 2017a,  Dartey 2012,  WHO 2014e,  Congo et al. 2017,  Dumont et al. 2009,  WHO 2014d,  van Hamersveld et al. 2012,  de Kok et al. 2017,  Bergh et al. 2011,  Belizan et al. 2011,  MCSP 2018,  Balogun and Musoke 2014,  Agaro et al. 2016,  du Châtelet et al. 2019 [unpublished],  MCSP 2017c,  Armstrong et al. 2014,  Rhoda et al. 2014,  Karamagi et al. 2018,  Kongnyuy and van den Broek 2008,  Mutsigiri-Murewanhema et al. 2017,  Hofman and Mohammed 2014,  Bandali et al. 2016,  Ministry of Health and Sanitation [Sierra Leone] 2017,  Lewis 2014b,  Mogobe et al. 2007,  Nyamtema et al. 2011,  Nyamtema et al. 2010,  Koblinsky et al. 2017,  Bayley et al. 2015,  Smith et al. 2017c, | Some records demonstrated how MPDSR functions well in settings with a culture of accountability, learning and improvement (WHO 2014d, Bandali et al. 2016, Belizan et al. 2011, Biswas et al. 2015, Manandhar 2004, Richard et al. 2009, de Kok et al. 2017). “Ideally, MDR meetings are conducted in a relatively egalitarian culture accepting of self-reflection and constructive criticism” (de Kok et al. 2017). A culture of trust was nurtured by strong leadership and continuous re-assurance of a “blame-free culture” (Kerber et al. 2015, du Châtelet et al. 2019 [unpublished], Grellier and Shome 2011, Belizan et al. 2011,). Open and enabling environments, which encourage active participation of all participations during meetings, was also found to improve implementation (MCSP 2017b, Dartey 2012).  *The audit method that encourages examination of practices, collegiality, documentation of decisions and evaluation of the results clashes with the bureaucratic organisation culture of the health system. Only a major reorganisation of the care system with increased professionalism and with caregivers taking responsibility for their actions will bring about this culture of quality. (ref Richard et al. 2009)*  A blame culture and punitive measures against frontline health providers was widely reported as a barrier to the implementation of the MPDSR across several context. The most common reasons cited were feeling threatened during the review meetings (Muffler et al. 2007, Balogun and Musoke 2014, 012, Dumont et al. 2009) and fearing legal action or punitive repercussions (3, WHO 2014b, WHO 2014a, Kongnyuy and van den Broek 2008, Smith et al. 2017b, Ethiopia Federal Ministry of Health et al. 2016, Agaro et al. 2016,Ministry of Health and Sanitation [Sierra Leone] 2017,WHO 2016, Hofman and Mohammed 2014, Mutsigiri-Murewanhema et al. 2017, Nyamtema et al. 2010, van Hamersveld et al. 2012); although some records noted a general culture of blame around MDPSR (Koblinsky et al. 2017, MCSP 2017a). The blame culture was reported among individual as well as between levels of the health system and between units (obstetrics vs pediatrics). Fear of punitive measures also was shown to influence the quality of the review process itself in that participants didn’t want to reveal gaps in case management (Muffler et al. 2007). A number of records found mixed reporting whereby some individuals or facilities, depending on study type, reported both a blame-free culture whilst also demonstrating or reporting a blame culture (Armstrong et al. 2014, Tapesana et al. 2017, Day 2006, MCSP 2018, MCSP 2017c, Lewis 2014b, Smith et al. 2017c, Congo et al. 2017, Bakker et al. 2011, du Châtelet et al. 2019 [unpublished]). The existence of blame culture and its effect at the subnational and national levels is however not adequately documented (de Kok et al. 2017). The identified reviews and commentaries in the scoping review also found fear of blame a common barrier to implementation (Raven et al. 2011, Kerber et al. 2015, 448, Scott and Danel 2016, Kongnyuy and van den Broek 2009, Lewis 2014a).  The continued prevalence of the blame culture may partially be attributed to the language and interpretation of the initial implementation. This quote from a study in Sierra Leone demonstrates this point: *“Fear of blame developed under the former maternal deaths audits (MDAs), which focused on establishing responsibilities when flaws occurred. The recent re-branding of meetings from “audits” to “reviews” aimed to emphasise this new blame-free approach.” (du Châtelet et al. 2019 [unpublished])* Although many guidelines have been updated, the previous interpretation of M/PDSR implementation continues to serve as basis for justifying fear of litigation among some health workers (van Hamersveld et al. 2012,du Châtelet et al. 2019 [unpublished],Agaro et al. 2016). One factor contributing to the fear amongst health workers was the absence of a strong MPDSR legal framework across all levels although the explicit aspects under fear for litigation are not exhaustively described (Koblinsky et al. 2017, WHO 2014e, Agaro et al. 2016,). Amidst this however, studies have described fear for litigation as having a positive effect on the implementation climate as form of accountability (Abebe et al. 2017, Bakker et al. 2011, MCSP 2017c). At the facility level, other strategies identified to minimizing acrimony, avoiding blame and recriminations included the mandatory attendance of audit meetings as well as codes of conduct or “audit charters” (Dartey 2012, MCSP 2017a, MCSP 2017b, MCSP 2017a, Richard et al. 2009, Dumont et al. 2009, Lewis 2014b).  Better data and reporting improves communication across the health system as well as between team members (Abebe et al. 2017).  *“MPDSR is a long and relatively complex health systems strengthening process that ultimately may reduce maternal and perinatal deaths but the evidence for such is weak at this point. It is a relatively new process (2013), and it will take time to know if it succeeds and the characteristics of the places where it succeeds.” (Koblinsky et al. 2017)* |
|  | Engaged leaders | Richard et al. 2009,  Bakker et al. 2011,  Muffler et al. 2007,  Kerber et al. 2015,  MCSP 2017a,  WHO 2014d,  van Hamersveld et al. 2012,  de Kok et al. 2017,  Bergh et al. 2011,  Belizan et al. 2011,  MCSP 2018,  du Châtelet et al. 2019 [unpublished],  MCSP 2017c,  Rhoda et al. 2014,  Hofman and Mohammed 2014,  Lewis 2014b,  Koblinsky et al. 2017,  Dumont et al. 2009,  Ethiopia Federal Ministry of Health et al. 2016,  Purandare et al. 2014,  WHO 2014b, | Many studies claim that strong leadership is required for successful implementation (Koblinsky et al. 2017, WHO 2014d, Kerber et al. 2015, Lewis 2014b, MCSP 2017c, MCSP 2017a, Purandare et al. 2014, Rhoda et al. 2014, van Hamersveld et al. 2012, Belizan et al. 2011, Bergh et al. 2011); while other studies recognize that the lack of leadership is a barrier (Ethiopia Federal Ministry of Health et al. 2016, du Châtelet et al. 2019 [unpublished], Muffler et al. 2007, Koblinsky et al. 2017). The lack of leaderships may be attributed to the limited institutionalization of MPDR across community, facility, sub-national and national levels across several LMICS (Hofman and Mohammed 2014).  At a national level, change agents may include individuals within the Ministries of Health (Balogun and Musoke 2014, Koblinsky et al. 2017), professional associations and partners such as UNFPA, WHO (WHO 2013, MCSP 2017c). At a sub-national level, leadership was identified as an enabler (MCSP 2017c, MCSP 2017b,MCSP 2017a, MCSP 2018, Kongnyuy and van den Broek 2008) as well as a barrier (Dumont et al. 2009, MCSP 2018, WHO 2014b, Dumont et al. 2009, Nam 2011). As noted in one study from Uganda: “DMOs have many competing priorities and the lack of district leadership/ownership hindered institutionalization of MDR (Nam 2011).” Agents of change the health facility level include individual health workers such as obstetricians, midwives, heads of maternity units or collective efforts of QI teams/MPDR committees (MCSP 2018, Hofman and Mohammed 2014). In addition to spearheading MPDSR elated roles, these individuals often have additional responsibilities such as being in-charges of department/units (Lewis 2014b, van Hamersveld et al. 2012, WHO 2014e, Dumont et al. 2009).  Only a few studies dive deep into understanding the attributes of strong leaderships or the perceived quality of leaders by others. Champions or agents of changes are described as highly motivated individuals (Belizan et al. 2011, Bergh et al. 2011, Lewis 2014b, Rhoda et al. 2014, Pattinson et al. 2009, WHO 2014e, WHO 2014c), although these studies do not investigate what motivates these leaders specifically. The important role of facilitation came out in a number of studies in terms of having a good chairperson or a person who is able to steer the conversation to be blame-free and productive (Bakker et al. 2011, de Kok et al. 2017, Dumont et al. 2009, Hofman and Mohammed 2014, Richard et al. 2009). One study said that “‘true leaders’ of the audit session … usually are the first to ask questions and start discussions. (Bakker et al. 2011)” |
| Domain 4: Outer setting | Policy and planning | Muffler et al. 2007,  Kerber et al. 2015,  MCSP 2017a,  WHO 2014d,  Bergh et al. 2011,  Belizan et al. 2011,  MCSP 2018,  du Châtelet et al. 2019 [unpublished],  MCSP 2017c,  Hofman and Mohammed 2014,  Lewis 2014b,  Koblinsky et al. 2017,  Ethiopia Federal Ministry of Health et al. 2016,  Purandare et al. 2014,  WHO 2014b,  MCSP 2017b,  Abebe et al. 2017,  Dartey 2012,  WHO 2014e,  Congo et al. 2017,  Balogun and Musoke 2014,  Agaro et al. 2016,  Armstrong et al. 2014,  Karamagi et al. 2018,  Kongnyuy and van den Broek 2008,  Mutsigiri-Murewanhema et al. 2017,  Bandali et al. 2016,  Ministry of Health and Sanitation [Sierra Leone] 2017,  Nyamtema et al. 2011,  Bayley et al. 2015,  Smith et al. 2017c,  WHO 2014c,  Smith et al. 2017b,  Pearson et al. 2009,  WHO 2013,  Tapesana et al. 2017,  Hadush et al. 2016,  Magoma et al. 2015  WHO 2016,  E4A 2012,  WHO 2014a, | Policy and planning for MPDSR includes related polices and guidelines, including national plans, death notification requirements and legal protection. National plans relating to M/PDSR may come in the form of a policy, guideline, or law as standalone documents or integrated components of wider national policies for maternal and newborn health. The scoping review identified studies that demonstrated integrated policy approaches (Bandali et al. 2016, Smith et al. 2017c, Tapesana et al. 2017, Agaro et al. 2016, Baleta 2011, Pearson et al. 2009,), as well as standalone national policies on maternal and/or perinatal deaths notification (Abebe et al. 2017, Balogun and Musoke 2014, Bayley et al. 2015, Dartey 2012, Ministry of Health and Sanitation [Sierra Leone] 2017,Koblinsky 2017, MCSP 2018, Pearson et al. 2009) and national M/PDSR related guidelines (Ethiopia Federal Ministry of Health et al. 2016, Dartey 2012, Ministry of Health and Sanitation [Sierra Leone] 2017, Bandali et al. 2016, Hofman and Mohammed 2014, MCSP 2017a, Karamagi et al. 2018, Kongnyuy and van den Broek 2008, Magoma et al. 2015, MCSP 2018, Nyamtema et al. 2011, MCSP 2017c, Mutsigiri-Murewanhema et al. 2017, MCSP 2017b, Smith et al. 2017b, De Brouwere et al. 2014, 59, Pearson et al. 2009). The number of countries reporting the existence of related policies and technical guidance has increased overtime (Bandali et al. 2016, Kerber et al. 2015, Smith et al. 2017b, Scott and Danel 2016). Some records note the limitations of this tracking process beyond national level (Bandali et al. 2016, Hadush et al. 2016, Kerber et al. 2015, Smith et al. 2017b, Pearson et al. 2009), while other studies report that the existence of national guidelines does not necessarily reflect subnational or facility level implementation (Bandali et al. 2016, Kerber et al. 2015, Magoma et al. 2015, Smith et al. 2017b, Muffler et al. 2007, Pearson et al. 2009). Other records found that the lack of national guidelines hinders implementation (Balogun and Musoke 2014, Magoma et al. 2015, Lewis 2014b, Scott and Danel 2016, Grellier and Shome 2011). While not sufficient to ensure implementation, national guidelines may help to unify fragmented MPDSR related processes, institutionalize practice (Belizan et al. 2011) and inform the process e.g. how to set up a committee (Nyamtema et al. 2011, Pearson et al. 2009). There may also be a lag time from policy adoption to implementation (Smith et al. 2017b). Only one study from India mentioned digital innovation in terms of the need of a clear road map for MPDSR related software implementation (Purandare et al. 2014).  Six records described the presence of a legal framework or protocol around death notification which obligates clinicians and managers to report on the deaths to a central system (Dartey 2012, Kerber et al. 2015, Lewis 2014a, Smith et al. 2017c, Pearson et al. 2009). Obligatory notification may demonstrate maternal mortality as a government priority adding additional pressure on practitioners (Mutsigiri-Murewanhema et al. 2017, Scott and Danel 2016). Even in settings with a legal framework on death notification, studies flagged that underreporting is often experienced and not all deaths are actually reviewed (Pearson et al. 2009,). Some articles report that legal measures linked to the M/PDSR process, particularly around liability and punitive measures hindered implementation (see inner setting; ref Koblinsky 2017, Lewis 2014a, Hadush et al. 2016). Smith and colleagues lay out what legal frameworks or safeguards entitle, and report that little information on such frameworks is available to countries (Smith et al. 2017c,). A brief prepared by Evidence 4 Action in 2012 clearly describes and summarizes the legal challenges around establishing and implementing a MDSR system, including confidentiality, liability and ethical considerations, and recommendations for overcoming these challenges in terms of establishing guidelines and legal reform (E4A 2012). |
|  | Resource support | Muffler et al. 2007,  MCSP 2017a,  WHO 2014d,  Bergh et al. 2011,  Belizan et al. 2011,  MCSP 2018,  du Châtelet et al. 2019 [unpublished],  Hofman and Mohammed 2014,  Lewis 2014b,  Purandare et al. 2014,  WHO 2014b,  Abebe et al. 2017,  Dartey 2012,  WHO 2014e,  Congo et al. 2017,  Agaro et al. 2016,  Bandali et al. 2016,  Smith et al. 2017c,  WHO 2014c,  Pearson et al. 2009,  WHO 2013,  Tapesana et al. 2017,  WHO 2016,  WHO 2014a,  Dumont et al. 2009,  Biswas et al. 2015,  Day 2006,  Grellier and Shome 2011  Nam 2011 | Resource support for M/PDSR was noted as an important component for implementation (Kerber et al. 2015, Smith et al. 2017c, WHO 2016); yet the literature showed mixed findings on whether specifically allocated resources are needed for M/PDSR and if so, how much and how it is budgeted. Some records recommended a budget line for MPDSR, at the different levels (Magoma et al. 2015, Pearson et al. 2009), with documented success in Burkina Faso, South Africa, Sri Lanka and Indonesia (Koblinsky et al. 2017, Bandali et al. 2016, Rhoda et al. 2014, Koblinsky et al. 2017, WHO 2014d, Belizan et al. 2011). In South Africa, Belizan and colleagues found that facilities with sustained perinatal death audit practice used their own resources (Belizan et al. 2011). The lack of budgets for MPDSR was reported to impede implementation in other records (Balogun and Musoke 2014, Hadush et al. 2016, Kongnyuy and van den Broek 2008, WHO 2014b, WHO 2014c, Hofman and Mohammed 2014, Baleta 2011). National or regional budgets specific to M/PDSR or for overall health services, were not reported to increase spending for M/PDSR in studies from Nigeria, Tanzania and Indonesia (Congo et al. 2017, MCSP 2017a, Magoma et al. 2015, Koblinsky et al. 2017).  In countries with an established MPDSR, government commitment and involvement is also evident through financial support for review activities, and administrative support through hosting secretariats and coordinating bodies within government institutions, such as in Malaysia and South Africa (Bandali et al. 2016, Smith et al. 2017c, Koblinsky et al. 2017, Pearson et al. 2009). Some studies linked levels of funding to political commitment and buy-in from government to MNH (Abebe et al. 2017, Smith et al. 2017c, Pearson et al. 2009,).  The importance of international mobilization of resources for MPDSR with different stakeholder groups (Hofman and Mohammed 2014,) and development partner support (Purandare et al. 2014, De Brouwere et al. 2014, Smith et al. 2017b, 953, Day 2006, Pearson et al. 2009) was noted. Support from external actors, such as donors and development partners, assisted in filling some of the budgetary gaps faced by MPDSR (WHO 2016, Koblinsky et al. 2017, Grellier and Shome 2011). Some studies showed that the audit process relied entirely on donor support (Congo et al. 2017, Hofman and Mohammed 2014, WHO 2014b), and a number of studies acknowledged that this cannot sustain practice (Congo et al. 2017, De Brouwere et al. 2014, Smith et al. 2017c, Grellier and Shome 2011, WHO 2014e, Day 2006, du Châtelet et al. 2019 [unpublished], Pearson et al. 2009). The literature recognizes the importance of international mobilization of resources for MPDSR (Purandare et al. 2014, Hofman and Mohammed 2014, De Brouwere et al. 2014, Smith et al. 2017b, 953, Day 2006, Pearson et al. 2009, WHO 2016, Koblinsky et al. 2017, Grellier and Shome 2011, Congo et al. 2017, Hofman and Mohammed 2014, WHO 2014b), and that dependence on development partners cannot sustain practice (Congo et al. 2017, De Brouwere et al. 2014, Smith et al. 2017c, Grellier and Shome 2011, WHO 2014e, Day 2006, du Châtelet et al. 2019 [unpublished], Pearson et al. 2009),  One study reported that MPDSR itself was used to mobilize resources for the process (Hofman and Mohammed 2014).    “Without government commitment and funds to scale-up countries are unable to continue to strengthening capacity of staff at all levels to conduct MDR – i.e. training on the MDR method in all facilities, and training for assessors on completing MDR forms, maternal death classification (using ICDMM) and formulating recommendations.” (Smith et al. 2017c) |
|  | External actors | Muffler et al. 2007,  MCSP 2017a,  Bergh et al. 2011,  Belizan et al. 2011,  MCSP 2018,  du Châtelet et al. 2019 [unpublished],  Hofman and Mohammed 2014,  Purandare et al. 2014,  Abebe et al. 2017,  Congo et al. 2017,  Agaro et al. 2016,  Bandali et al. 2016,  Smith et al. 2017c,  Pearson et al. 2009,  WHO 2016,  WHO 2014a,  Dumont et al. 2009,  Biswas et al. 2015,  Day 2006,  Grellier and Shome 2011  Nam 2011  MCSP 2017c,  Koblinsky et al. 2017,  MCSP 2017b,  Balogun and Musoke 2014,  Kongnyuy and van den Broek 2008,  Ministry of Health and Sanitation [Sierra Leone] 2017,  Bayley et al. 2015,  Smith et al. 2017b,  Richard et al. 2009,  Bakker et al. 2011, | Some studies recognized external actors participation in the national MPDSR process e.g. professional association members and UN agency representatives (De Brouwere et al. 2014, Hofman and Mohammed 2014, Koblinsky 2017, Lewis 2014b, WHO 2014e, WHO 2014d); while other studies noted this engagement was lacking (Bandali et al. 2016, Ministry of Health and Sanitation [Sierra Leone] 2017). A few studies cautioned against expanding external engagement (Bandali et al. 2016, Ministry of Health and Sanitation [Sierra Leone] 2017), such as to private sector (Balogun and Musoke 2014), communities, civil society and local authorities (Tapesana et al. 2017), partially due to legal risks (WHO 2014e, du Châtelet et al. 2019 [unpublished]); whereas other studies encouraged engagement with private sector, community, professional associations and others (du Châtelet et al. 2019 [unpublished], Pearson et al. 2009, Bayley et al. 2015, Hadush et al. 2016, Kerber et al. 2015). The level of engagement and roles varied by study including support for developing guidelines and tools, providing training and ongoing technical assistance, and resource support.   - support developing subnational or facility guidelines (Hofman and Mohammed 2014, Biswas et al. 2015, Smith et al. 2017c, MCSP 2017b, Dumont et al. 2009) (commentary/review Biswas 2017, Lewis 2014a) - support developing national guidelines (MCSP 2017b, Smith et al. 2017c, Smith et al. 2017b, Pearson et al. 2009) (commentary Biswas 2017) - Support developing tools MCSP 2017b, Hofman and Mohammed 2014, Biswas et al. 2015, Dumont et al. 2009, Richard et al. 2009, Nam 2011, Purandare et al. 2014) (commentary Biswas 2017) - Subnational committee formation and training (Agaro et al. 2016, Dumont et al. 2009, Richard et al. 2009, Nam 2011, Smith et al. 2017b, Agaro et al. 2016, MCSP 2017c, MCSP 2017b,) (commentary Biswas 2017, De Brouwere et al. 2014) - Facility level training (Dumont et al. 2009, Nam 2011, Agaro et al. 2016, MCSP 2017b, Smith et al. 2017c,) (commentary/review Lewis 2014a, Pattinson et al. 2009) - National resource support (MCSP 2017b, Smith et al. 2017c, Smith et al. 2017b, Pearson et al. 2009,) (commentary/review Lewis 2014a, Pattinson et al. 2009) - Subnational resource support (MCSP 2017b) - Facility level resource support (Smith et al. 2017c) - Overall health system support (Bakker et al. 2011, Smith et al. 2017c, Smith et al. 2017b, Dumont et al. 2009, Koblinsky et al. 2017) (commentary/review Biswas 2017, Lewis 2014a, Pattinson et al. 2009) - National or subnational technical assistance (Bandali et al. 2016, Smith et al. 2017c, Smith et al. 2017b, Dumont et al. 2009, Koblinsky et al. 2017, 959, Pearson et al. 2009, Richard et al. 2009, Biswas et al. 2015, du Châtelet et al. 2019 [unpublished], Day 2006, Grellier and Shome 2011, Kongnyuy and van den Broek 2008, Ministry of Health and Sanitation [Sierra Leone] 2017,) (commentary/review Biswas 2017, Lewis 2014a) - active participation in training and oversight (Agaro et al. 2016, Bakker et al. 2011, Lewis 2014a, Smith et al. 2017a, Pearson et al. 2009) to resource support (WHO 2014e, WHO 2016).     At a subnational or facility level, strong national actors have an external influence on MPDSR processes, and successful implementation can be attributed to MOH, often with strong national committee (Bayley et al. 2015, MCSP 2017a, MCSP 2018, Smith et al. 2017a, Smith et al. 2017b, du Châtelet et al. 2019 [unpublished], Pearson et al. 2009). As articulated by Pearson and colleagues (2009): “A national interest group and dedicated national committee supported by the government and professional bodies are a critical driving force behind MDR in those countries where progress is being made” (Pearson et al. 2009,). Our review showed that many country governments through their ministries of health have endorsed MPDSR and played a role in the development of guidelines and roll out of implementation, such as Ethiopia (3), Tanzania (Armstrong et al. 2014), Sudan (Balogun and Musoke 2014), Bangladesh (Biswas 2017), Burkina Faso (Congo et al. 2017), Cameroon (De Brouwere et al. 2014), India (Purandare et al. 2014,), and Uganda (Agaro et al. 2016), and South Africa (Belizan et al. 2011). In some instances, strong national ownership was seen as “top down approaches” (Balogun and Musoke 2014, Belizan et al. 2011).  At a national level, some studies acknowledged MPDSR was externally driven by WHO, UNFPA and donor agencies, though supported/endorsed by the Government (Smith et al. 2017c, De Brouwere et al. 2014, Bandali et al. 2016, Biswas et al. 2015), and many countries adapted their guidelines from the global guidance provided by WHO (Abebe et al. 2017, Biswas et al. 2015). At national, subnational and facility levels, development partners (UN agencies and NGOs) and professional associations have played a role in both supporting implementation processes e.g. developing guidelines, training facility staff and mobilizing resources as well as pressuring governments (mostly at national level) to implement (Agaro et al. 2016, Bakker et al. 2011, Biswas et al. 2015, Lewis 2014a, MCSP 2017a, Purandare et al. 2014, Smith et al. 2017b, Smith et al. 2017a, Smith et al. 2017c, WHO 2014e, Koblinsky et al. 2017, Pearson et al. 2009). For example, in Nigeria, “Saving Newborn Lives project of Save the Children, in collaboration with the Nigerian Society of Neonatal Medicine (NISONM), advocated for the integration of stillbirths and neonatal deaths into the MDSR system, and supported the development of an integrated MPDSR guideline and tools with the FMOH in 2015” (MCSP 2017a). External actors also supported the process of designing and implementing electronic information systems in India (Purandare et al. 2014). The absence of external actor engagement as a barrier to implementation was noted in some studies (Balogun and Musoke 2014, Bandali et al. 2016, Belizan et al. 2011, Ministry of Health and Sanitation [Sierra Leone] 2017, Tapesana et al. 2017).  The different types of community links to facility-based MPDSR are mentioned but not studied (Armstrong et al. 2014, Ethiopia Federal Ministry of Health et al. 2016, Dartey 2012, WHO 2014d, du Châtelet et al. 2019 [unpublished], WHO 2014a, WHO 2014b, WHO 2014e, Abebe et al. 2017, Hofman and Mohammed 2014, Kerber et al. 2015, Kongnyuy and van den Broek 2008, Magoma et al. 2015, MCSP 2017c, WHO 2014e, du Châtelet et al. 2019 [unpublished], Lewis 2014a, Pattinson et al. 2009, Bayley et al. 2015, Hadush et al. 2016). A number of studies discussed the different types of community links to facility-based MPDSR (Armstrong et al. 2014, Ethiopia Federal Ministry of Health et al. 2016, Dartey 2012, WHO 2014d, du Châtelet et al. 2019 [unpublished], WHO 2014a, WHO 2014b, WHO 2014e,). Community consultation and engagement on the response portion of MPDSR improved implementation of actions in some studies (Abebe et al. 2017, Hofman and Mohammed 2014, Kerber et al. 2015, Kongnyuy and van den Broek 2008, Magoma et al. 2015, MCSP 2017c, WHO 2014e, du Châtelet et al. 2019 [unpublished]). Community-reviews, led by medical doctors, have demonstrated promise, but need to be carefully planned in order to engage the right people, depending on the context, and ensure blame free environment (Lewis 2014a, Pattinson et al. 2009). One study found increased commitment by health workers to fulfil their commitments when they publicize their plans at the community feedback meetings (Bayley et al. 2015). The Community-linked maternal death review tested in Malawi showed potential to use the partnership of communities and health facility representatives to investigate and respond to maternal deaths (Bayley et al. 2015, Hadush et al. 2016). Low levels of community engagement or participation in MPDSR process were also recognized as a barrier (Ministry of Health and Sanitation [Sierra Leone] 2017, MCSP 2017a, MCSP 2018, Tapesana et al. 2017), with specific challenges noted around data collection of deaths in the community (Agaro et al. 2016, Balogun and Musoke 2014, Kerber et al. 2015, Mogobe et al. 2007,). Facility staff may fear punitive measures by the community for deaths; likewise communities are reluctant to report deaths due to fear of punitive measures by local systems, such as bylaws preventing women from delivering at home in Sierra Leone (E4A 2012, Ministry of Health and Sanitation [Sierra Leone] 2017).  Maintaining stakeholder confidence and commitment has been recommended for implementation (Hadush et al. 2016). Yet, the nature of external actor involvement and the impact on the process requires more systematically investigation and likely depends on context.  Other reviews also identified the various roles of external actors including involvement of developmental partners in the initial implementation phases, support in developing national guidelines, tools, formation of committees, technical assistance and trainings (Biswas 2017, Pattinson et al. 2009). Lewis’ commentary also unpacked the role of external actors on implementation based on the experience from the implementation of FIGO’s LOGIC project (Lewis 2014a). |
|  | Political prioritization | MCSP 2017a,  MCSP 2018,  du Châtelet et al. 2019 [unpublished],  Abebe et al. 2017,  Agaro et al. 2016,  Bandali et al. 2016,  Day 2006,  MCSP 2017b,  WHO 2013,  Kerber et al. 2015, | National political commitment and government leadership were mentioned in a number of studies and alluded to possible pressure on the health system to implement MPDSR (Abebe et al. 2017, Bandali et al. 2016, Biswas 2017, Kerber et al. 2015, Day 2006, MCSP 2017b, MCSP 2017a, MCSP 2018, du Châtelet et al. 2019 [unpublished]). Abebe and colleagues described the politicization of maternal deaths linked to national pride to achieve the Millennium Development Goals in Ethiopia as a driving factor for health workers to implement of MDSR to prevent future deaths (Abebe et al. 2017). A study from Sierra Leone also found national and subnational stakeholders to believe that political prioritization of maternal health was a pre-prerequisite to implementation: “many respondents thought that for a successful implementation of MDRs, political prioritisation of maternal health within health and government planning was needed” (du Châtelet et al. 2019 [unpublished]). Gaps relating to actual political prioritization of MPDSR remain glaring as reflected in the challenges of inadequate funding for MPDSR across all health system levels (du Châtelet et al. 2019 [unpublished], Agaro et al. 2016).  Global commitments to the MDGs or regional commitments, such as the African Union’s initiative CARMMA, may have led to additional pressure on national governments to implement (Kerber et al. 2015, WHO 2013, Bandali et al. 2016), but this was not systematically assessed. |
|  | Pressure to implement | du Châtelet et al. 2019 [unpublished],  Agaro et al. 2016,  Bandali et al. 2016,  Day 2006,  WHO 2013,  Kerber et al. 2015,  Muffler et al. 2007,  Bergh et al. 2011,  Belizan et al. 2011,  Smith et al. 2017c,  Biswas et al. 2015,  Grellier and Shome 2011  Nam 2011  Bayley et al. 2015,  Smith et al. 2017b,  Rhoda et al. 2014,  WHO 2011 | Few studies specifically looked at perceptions around pressure to implement although some studies allude to pressure on governments to implement (Agaro et al. 2016, Bandali et al. 2016, Smith et al. 2017c, Smith et al. 2017b, Muffler et al. 2007, du Châtelet et al. 2019 [unpublished], Day 2006, Grellier and Shome 2011, WHO 2013, WHO 2011). Regular reporting to WHO and other agencies on policy uptake could be seen another form of pressure or accountability mechanism (Pearson et al. 2009, 953, Bandali et al. 2016, Kerber et al. 2015, Smith et al. 2017c). Implementation pressure was especially noted in the pilot studies (Bayley et al. 2015, Grellier and Shome 2011). At the facility level, peer pressure for system wide uptake came in the form of outreach visits from regional specialists and reporting requirements by sub-national structures (Belizan et al. 2011, Biswas 2017, Biswas et al. 2015, Rhoda et al. 2014). "The [facility death review] system encouraged healthcare providers to report each facility death in the system followed by a review” (Biswas et al. 2015). The lack of national and sub-national pressure to implement was also recognized as barrier to implementation (Balogun and Musoke 2014, Nam 2011). The pressure to implement within the team was identified in one study from South Africa where “a critical mass receptive to the potential benefits of using PPIP” was shown to facilitate commitment to implementation at hospital level (Belizan et al. 2011). |
|  | Linkages and networks between levels | du Châtelet et al. 2019 [unpublished],  Agaro et al. 2016,  Bandali et al. 2016,  Day 2006,  Kerber et al. 2015,  Muffler et al. 2007,  Bergh et al. 2011,  Belizan et al. 2011,  Smith et al. 2017c,  Bayley et al. 2015,  Smith et al. 2017b,  Rhoda et al. 2014,  MCSP 2017a,  MCSP 2018,  Abebe et al. 2017,  MCSP 2017b,  Hofman and Mohammed 2014,  Congo et al. 2017,  Pearson et al. 2009,  MCSP 2017c,  Balogun and Musoke 2014,  Kongnyuy and van den Broek 2008,  Ministry of Health and Sanitation [Sierra Leone] 2017,  Richard et al. 2009,  WHO 2014d,  Dartey 2012,  WHO 2014c,  Ethiopia Federal Ministry of Health et al. 2016,  Armstrong et al. 2014,  Nyamtema et al. 2011,  Hadush et al. 2016,  Magoma et al. 2015  Nyamtema et al. 2010,  Ethiopian Public Health Institute et al. 2017 | The level of connectedness and networks between health system levels, different sites and different role players influences implementation from a systems lens. Overall communication across levels was described in a number of studies as occurring, e.g. notification forms shared, dissemination of findings and actions, data and information shared through clear communication channels (Bayley et al. 2015, Magoma et al. 2015, 117, Richard et al. 2009, MCSP 2017b, Ethiopia Federal Ministry of Health et al. 2016, Belizan et al. 2011, Rhoda et al. 2014, Dartey 2012, WHO 2014d). A couple of studies found that MDPSR processes strengthened communication across the levels of the health system (Abebe et al. 2017, Belizan et al. 2011, Bergh et al. 2011) and between stakeholders (Bandali et al. 2016, Bayley et al. 2015, MCSP 2018). When implemented well, supportive supervision was found to facilitate MPDSR implementation (Abebe et al. 2017, Kongnyuy and van den Broek 2008, Dartey 2012, 5Bayley et al. 2015,). When implemented poorly, supportive supervision can have damaging effects: “the secretariat attending meetings and documenting case files (photocopies) seems to have negative impact on staff's willingness to cooperate” (Smith et al. 2017b). When not implemented at all, there is weak collaboration and partnerships between subnational and facility level MPDSR teams (Agaro et al. 2016, Smith et al. 2017b, Muffler et al. 2007).  Implementation was supported by existing strong communication channels (MCSP 2017c, Nyamtema et al. 2011, Smith et al. 2017c) and well-defined pathways around the flow of data and information relating to MPDSR (Belizan et al. 2011, Rhoda et al. 2014, Dartey 2012). The lack of connectivity was identified as a barrier to implementation in other studies, even when systems and guidelines were in place (Agaro et al. 2016, Balogun and Musoke 2014, Hofman and Mohammed 2014, Nyamtema et al. 2010, Ethiopia Federal Ministry of Health et al. 2016, Armstrong et al. 2014, Ministry of Health and Sanitation [Sierra Leone] 2017, MCSP 2017a, MCSP 2018, du Châtelet et al. 2019 [unpublished]). The absence of an adequate and coherent framework to guide both local and national communication/dissemination of MPSR recommendations was a barrier to communication beyond the health facility level (Agaro et al. 2016, du Châtelet et al. 2019 [unpublished], Dumont et al. 2009, MCSP 2018, MCSP 2017b, Koblinsky et al. 2017). As result, there exists lack of clarity of roles and duplication of activities among stakeholders at the sub-national and national levels (MCSP 2018, WHO 2014e, du Châtelet et al. 2019 [unpublished]). Good communication between health staff in facilities (MCSP 2018), as well as between facilities (Kongnyuy and van den Broek 2008, Day 2006) about MPDSR related findings and actions, also supported implementation efforts. Poor communication between staff hindered implementation in other studies (WHO 2014c, du Châtelet et al. 2019 [unpublished]).  The linkage into existing health system structures may also influence implementations, such as integrating the surveillance into other health programming or integrating activities into other MNH programs (Abebe et al. 2017, Bandali et al. 2016, Biswas 2017, Pearson et al. 2009). Vertically designed programs prevented uptake and sustainability, as demonstrated in Sudan overall program (Balogun and Musoke 2014). |

## Table S4.5. Components of the audit cycle described

| **Reference (Author, year)** | **Type of audit** | **Audit cycle explained in theory** | **Full audit process described** | **Notification**  **described** | **data collection**  **described** | **review process**  **described** | **Recommendations**  **described** | **Evaluation**  **described** | **multiple steps described** |
| --- | --- | --- | --- | --- | --- | --- | --- | --- | --- |
| Abebe et al. 2017, | MDSR | yes | no | no | yes | yes | yes | no | yes |
| Agaro et al. 2016, | MPDR | no | no | no | no | no | yes | no | no |
| Armstrong et al. 2014, | MPDR | yes | yes | yes | yes | yes | yes | yes | yes |
| Bakker et al. 2011, | obstetric Critical incident audit | yes | no | no | no | yes | yes | no | yes |
| Balogun and Musoke 2014, | MDR | yes | yes | yes | yes | yes | yes | yes | yes |
| Bandali et al. 2016, | MDSR | yes | no | yes | no | no | yes | yes | yes |
| Bayley et al. 2015, | CLMDR | yes | yes | yes | yes | yes | yes | yes | yes |
| Belizan et al. 2011, | perinatal death audit program | yes | yes | yes | yes | yes | yes | yes | yes |
| Bergh et al. 2011, | perinatal death audit | yes | yes | yes | yes | yes | yes | yes | yes |
| Biswas et al. 2015, | facility maternal and neonatal death review | yes | no | yes | yes | yes | yes | no | yes |
| Combs Thorsen et al. 2014, | MDR | yes | yes | yes | yes | yes | yes | yes | yes |
| Congo et al. 2017, | maternal death audits | yes | no | no | no | yes | yes | yes | yes |
| Dartey 2012, | MDR | yes | yes | yes | yes | yes | yes | yes | yes |
| Day 2006, | perinatal and maternal death audit | yes | yes | yes | yes | yes | yes | yes | yes |
| de Kok et al. 2017, | MDR | yes | no | no | no | no | no | no | no |
| Ministry of Health and Sanitation [Sierra Leone] 2017, | MDSR | yes | yes | yes | yes | yes | yes | yes | yes |
| du Châtelet et al. 2019 [unpublished], | maternal death review | yes | no | no | yes | yes | yes | yes | yes |
| Dumont et al. 2013, | MDR | no | no | no | no | no | no | no | no |
| Dumont et al. 2009, | MDR | yes | no | no | no | no | no | no | no |
| Hadush et al. 2016, | MDSR | no | no | no | no | no | no | no | no |
| E4A 2012, | MDSR | no | no | no | no | no | no | no | no |
| Ethiopia Federal Ministry of Health et al. 2016, | MDSR | yes | no | no | no | no | no | no | no |
| Ethiopian Public Health Institute et al. 2017 | MDSR | yes | no | no | no | no | no | no | no |
| Grellier and Shome 2011 | mix | no | no | no | no | no | no | no | no |
| Hofman and Mohammed 2014, | MDR | yes | no | yes | yes | yes | no | no | yes |
| Karamagi et al. 2018, | MPDSR | no | no | no | no | no | no | no | no |
| Kerber et al. 2015, | perinatal death audit | yes | yes | no | yes | yes | yes | yes | yes |
| Koblinsky et al. 2017, | MPDSR | yes | no | no | yes | yes | yes | yes | yes |
| Kongnyuy and van den Broek 2008, | maternal death reviews | yes | no | no | no | no | no | no | no |
| Lewis 2014b, | maternal death and near-death reviews | yes | no | no | no | no | no | no | no |
| Magoma et al. 2015 | maternal death reviews | no | no | no | no | no | no | no | no |
| Manandhar 2004, | perinatal death audit | yes | no | no | yes | yes | no | no | yes |
| MCSP 2017c, | MPDSR | yes | yes | yes | yes | yes | yes | yes | yes |
| MCSP 2017b, | MPDSR | yes | yes | yes | yes | yes | yes | yes | yes |
| MCSP 2017a, | MPDSR | yes | yes | yes | yes | yes | yes | yes | yes |
| MCSP 2018, | MPDSR | yes | yes | yes | yes | yes | yes | yes | yes |
| Mogobe et al. 2007, | maternal death and morbidity reviews | no | no | yes | yes | no | no | no | yes |
| Muffler et al. 2007, | mix | yes | no | no | yes | yes | yes | no | yes |
| Mutsigiri-Murewanhema et al. 2017, | maternal mortality surveillance system | no | no | yes | yes | no | no | no | yes |
| Nam 2011 | maternal death reviews | no | no | no | no | no | no | no | no |
| Nyamtema et al. 2011, | maternal death and morbidity reviews | no | no | no | no | no | no | no | no |
| Nyamtema et al. 2010, | maternal and perinatal death audits | yes | no | no | no | yes | yes | yes | yes |
| Pearson et al. 2009, | maternal death reviews | no | no | no | no | no | no | no | no |
| Purandare et al. 2014, | maternal death reviews | no | no | yes | yes | no | no | no | yes |
| Rhoda et al. 2014, | perinatal death audit | yes | no | no | yes | yes | yes | no | yes |
| Richard et al. 2009, | medical audit | yes | no | no | no | no | no | no | no |
| Smith et al. 2017b, | MDSR | yes | yes | yes | yes | yes | yes | yes | yes |
| Smith et al. 2017c, | MDSR | yes | no | no | yes | yes | yes | yes | yes |
| Tapesana et al. 2017, | MDSR | yes | no | yes | yes | yes | no | no | yes |
| van Hamersveld et al. 2012, | obstetric audit | yes | no | no | yes | yes | yes | yes | yes |
| WHO 2011 | maternal, perinatal, neonatal health surveillance systems | no | no | no | no | no | no | no | no |
| WHO 2013, | MDSR | yes | no | yes | no | yes | yes | no | yes |
| WHO 2016, | MPDSR | yes | no | no | no | no | no | no | no |
| WHO 2014a, | mix | yes | no | no | no | no | no | no | no |
| WHO 2014b, | maternal death reviews | yes | no | no | no | no | no | no | no |
| WHO 2014e, | mix | yes | no | no | no | no | no | no | no |
| WHO 2014c, | maternal death review | no | no | no | no | no | no | no | no |
| WHO 2014d, | mix | yes | yes | yes | yes | yes | yes | yes | yes |
|  |  |  |  |  |  |  |  |  |  |
| ***Total (yes)*** |  | ***43*** | ***16*** | ***23*** | ***30*** | ***31*** | ***30*** | ***23*** | ***35*** |
| ***% (yes)*** |  | ***74%*** | ***28%*** | ***40%*** | ***52%*** | ***53%*** | ***52%*** | ***40%*** | ***60%*** |

REFERENCES

Abebe, B., Busza, J., Hadush, A., Usmael, A., Zeleke, A. B., Sita, S., Hailu, S. & Graham, W. J. 2017. 'We identify, discuss, act and promise to prevent similar deaths': a qualitative study of Ethiopia's Maternal Death Surveillance and Response system. *BMJ Glob Health,* 2**,** e000199.

Agaro, C., Beyeza-Kashesya, J., Waiswa, P., Sekandi, J. N., Tusiime, S., Anguzu, R. & Kiracho, E. E. 2016. The conduct of maternal and perinatal death reviews in Oyam District, Uganda: a descriptive cross-sectional study. *BMC Womens Health,* 16**,** 38.

Armstrong, C. E., Lange, I. L., Magoma, M., Ferla, C., Filippi, V. & Ronsmans, C. 2014. Strengths and weaknesses in the implementation of maternal and perinatal death reviews in Tanzania: perceptions, processes and practice. *Trop Med Int Health,* 19**,** 1087-95.

Bakker, W., van den Akker, T., Mwagomba, B., Khukulu, R., van Elteren, M. & van Roosmalen, J. 2011. Health workers' perceptions of obstetric critical incident audit in Thyolo District, Malawi. *Trop Med Int Health,* 16**,** 1243-50.

Baleta, A. 2011. South Africa takes steps to reduce perinatal mortality. *The Lancet,* 377**,** 1303-1304.

Balogun, H. A. & Musoke, S. B. 2014. *The barriers of maternal death review implementation in Sudan - a qualitative assessment* Masters of Medical Science, Karolinska Institutet.

Bandali, S., Thomas, C., Hukin, E., Matthews, Z., Mathai, M., Ramachandran Dilip, T., Roos, N., Lawley, R., Igado, O. & Hulton, L. 2016. Maternal Death Surveillance and Response Systems in driving accountability and influencing change. *Int J Gynaecol Obstet,* 135**,** 365-371.

Bayley, O., Chapota, H., Kainja, E., Phiri, T., Gondwe, C., King, C., Nambiar, B., Mwansambo, C., Kazembe, P., Costello, A., Rosato, M. & Colbourn, T. 2015. Community-linked maternal death review (CLMDR) to measure and prevent maternal mortality: a pilot study in rural Malawi. *BMJ Open,* 5**,** e007753.

Belizan, M., Bergh, A. M., Cilliers, C., Pattinson, R. C., Voce, A. & Synergy, G. 2011. Stages of change: A qualitative study on the implementation of a perinatal audit programme in South Africa. *BMC Health Serv Res,* 11**,** 243.

Bergh, A. M., Pattinson, R., Belizan, M., Cilliers, C., Jackson, D., Kerber, K., Philpott, R. H., Voce, A. & for the Synergy Group 2011. Completing the audit cycle for quality care in perinatal, newborn and child health. Pretoria: Medical Research Council of South Africa.

Biswas, A. 2017. Shifting paradigm of maternal and perinatal death review system in Bangladesh: A real time approach to address sustainable developmental goal 3 by 2030. *F1000Res,* 6**,** 1120.

Biswas, A., Rahman, F., Eriksson, C., Halim, A. & Dalal, K. 2015. Facility Death Review of Maternal and Neonatal Deaths in Bangladesh. *PLoS One,* 10**,** e0141902.

Buchmann, E. J. 2014. Towards greater effectiveness of perinatal death audit in low- and middle-income countries. *BJOG,* 121 Suppl 4**,** 134-6.

Combs Thorsen, V., Sundby, J., Meguid, T. & Malata, A. 2014. Easier said than done!: methodological challenges with conducting maternal death review research in Malawi. *BMC Med Res Methodol,* 14**,** 29.

Congo, B., Sanon, D., Millogo, T., Ouedraogo, C. M., Yameogo, W. M. E., Meda, Z. C. & Kouanda, S. 2017. Inadequate programming, insufficient communication and non-compliance with the basic principles of maternal death audits in health districts in Burkina Faso: a qualitative study. *Reprod Health,* 14**,** 121.

Dartey, A. F. 2012. *The role of midwives in the implementation of maternal death review (MDR) in health facilities in Ashanti region, Ghana.* University of the Western Cape.

Day, L. T. 2006. Evaluation of Perinatal and Maternal Death Audit in pilot trained facilities in Bangladesh. Dinajpur, Bangladesh: Internal report for Saving Newborn Lives, Save the Children.

De Brouwere, V., Delvaux, T. & Leke, R. J. 2014. Achievements and lessons learnt from facility-based maternal death reviews in Cameroon. *BJOG,* 121 Suppl 4**,** 71-4.

de Kok, B., Imamura, M., Kanguru, L., Owolabi, O., Okonofua, F. & Hussein, J. 2017. Achieving accountability through maternal death reviews in Nigeria: a process analysis. *Health Policy Plan*.

du Châtelet, A., Zamboni, K., Fornah, F., Yilla, M. & Nam, S. 2019 [unpublished]. Barriers and enablers to the implementation of Maternal Death Reviews to improve quality of care in Sierra Leone. *draft paper*.

Dumont, A., Fournier, P., Abrahamowicz, M., Traore, M., Haddad, S., Fraser, W. D. & group, Q. r. 2013. Quality of care, risk management, and technology in obstetrics to reduce hospital-based maternal mortality in Senegal and Mali (QUARITE): a cluster-randomised trial. *Lancet,* 382**,** 146-57.

Dumont, A., Tourigny, C. & Fournier, P. 2009. Improving obstetric care in low-resource settings: implementation of facility-based maternal death reviews in five pilot hospitals in Senegal. *Hum Resour Health,* 7**,** 61.

E4A 2012. Maternal death surveillance and response systems: overcoming legal challenges and creating an enabling environment. MDSR Action Network.

Ethiopia Federal Ministry of Health, WHO & E4A 2016. National Report on MDSR Data from 2006-2007 EFY. Addis Ababa, Ethiopia: Ethiopian Public Health Institute.

Ethiopian Public Health Institute, Ethiopia Federal Ministry of Health, WHO & E4A 2017. 2nd Ethiopian National Maternal Death Surveillance and Response (MDSR) report. Addis Ababa, Ethiopia: Ethiopian Public Health Institute.

Grellier, R. & Shome, P. 2011. FIGO Saving mothers and newborn project: Summary evaluation. Options.

Hadush, A., Ibro, A., Abebe, B., Huszar, I., Lawley, R. & Graham, W. 2016. Global experience with Maternal Death Surveillance and Response: building for the long-term. E4A Ethiopia implemented by WHO, the University of Aberdeen and Options with funding from the UK Department for International Development and the Bill and Melinda Gates Foundation.

Hofman, J. J. & Mohammed, H. 2014. Experiences with facility-based maternal death reviews in northern Nigeria. *Int J Gynaecol Obstet,* 126**,** 111-4.

Hussein, J., Hirose, A., Owolabi, O., Imamura, M., Kanguru, L. & Okonofua, F. 2016. Maternal death and obstetric care audits in Nigeria: a systematic review of barriers and enabling factors in the provision of emergency care. *Reprod Health,* 13**,** 47.

Karamagi, E., Sensalire, S., Chakura, A. & Rahimzai, M. 2018. Maternal Perinatal Death Surveillance Review (MPDSR): Strengthening Reviews to Save More Lives in Uganda Uganda: USAID ASSIST Project.

Kerber, K. J., Mathai, M., Lewis, G., Flenady, V., Erwich, J. J., Segun, T., Aliganyira, P., Abdelmegeid, A., Allanson, E., Roos, N., Rhoda, N., Lawn, J. E. & Pattinson, R. 2015. Counting every stillbirth and neonatal death through mortality audit to improve quality of care for every pregnant woman and her baby. *BMC Pregnancy Childbirth,* 15 Suppl 2**,** S9.

Koblinsky, M. 2017. Maternal Death Surveillance and Response: A Tall Order for Effectiveness in Resource-Poor Settings. *Glob Health Sci Pract,* 5**,** 333-337.

Koblinsky, M., Kaptiningsih, A. & Fitriyani. 2017. Indonesia: Reducing maternal & perinatal deaths through MPDSR – mapping the possibilities. Draft report for USAID prepared by Management Systems International.

Kongnyuy, E. & van den Broek, N. 2009. Audit for maternal and newborn health services in resource-poor countries. *BJOG: An International Journal of Obstetrics & Gynaecology,* 116**,** 7-10.

Kongnyuy, E. J. & van den Broek, N. 2008. The difficulties of conducting maternal death reviews in Malawi. *BMC Pregnancy Childbirth,* 8**,** 42.

Lewis, G. 2014a. The cultural environment behind successful maternal death and morbidity reviews. *Bjog,* 121 Suppl 4**,** 24-31.

Lewis, G. 2014b. Emerging lessons from the FIGO LOGIC initiative on maternal death and near-miss reviews. *Int J Gynaecol Obstet,* 127 Suppl 1**,** S17-20.

Magoma, M., Massinde, A., Majinge, C., Rumanyika, R., Kihunrwa, A. & Gomodoka, B. 2015. Maternal death reviews at Bugando hospital north-western Tanzania: a 2008-2012 retrospective analysis. *BMC Pregnancy Childbirth,* 15**,** 333.

Manandhar, D. S. 2004. Perinatal death audit. *Kathmandu Univ Med J (KUMJ),* 2**,** 375-83.

Martin Hilber, A., Blake, C., Bohle, L. F., Bandali, S., Agbon, E. & Hulton, L. 2016. Strengthening accountability for improved maternal and newborn health: A mapping of studies in Sub-Saharan Africa. *Int J Gynaecol Obstet,* 135**,** 345-357.

MCSP 2017a. Assessment of Maternal and Perinatal Death Surveillance and Response Implementation in Nigeria. Washington, DC: Maternal Child Survival Program.

MCSP 2017b. Assessment of Maternal and Perinatal Death Surveillance and Response Implementation in Rwanda. Washington, DC: Maternal Child Survival Program.

MCSP 2017c. Assessment of Maternal and Perinatal Death Surveillance and Response Implementation in Zimbabwe. Washington, DC: Maternal Child Survival Program.

MCSP 2018. Assessment of Maternal and Perinatal Death Surveillance and Response (MPDSR) Implementation in Kagera and Mara Region, Tanzania. Washington, DC: Maternal Child Survival Program.

Ministry of Health and Sanitation [Sierra Leone] 2017. Maternal Death Surveillance and Response: Annual Report 2016. Free Town, Sierra Leone: Directorate of Reproductive & Child Health, Ministry of Health and Sanitation [Sierra Leone].

Mogobe, K. D., Tshiamo, W. & Bowelo, M. 2007. Monitoring maternity mortality in Botswana. *Reprod Health Matters,* 15**,** 163-71.

Muffler, N., Trabelssi Mel, H. & De Brouwere, V. 2007. Scaling up clinical audits of obstetric cases in Morocco. *Trop Med Int Health,* 12**,** 1248-57.

Mutsigiri-Murewanhema, F., Mafaune, P. T., Juru, T., Gombe, N. T., Bangure, D., Mungati, M. & Tshimanga, M. 2017. Evaluation of the maternal mortality surveillance system in Mutare district, Zimbabwe, 2014-2015: a cross sectional study. *Pan Afr Med J,* 27**,** 204.

Nam, S. 2011. Final Evaluation: FIGO Saving Mothers and Newborns Project in Uganda: Reduction of Maternal and Newborn Mortality in Uganda. Options.

Nyamtema, A. S., de Jong, A. B., Urassa, D. P. & van Roosmalen, J. 2011. Using audit to enhance quality of maternity care in resource limited countries: lessons learnt from rural Tanzania. *BMC Pregnancy Childbirth,* 11**,** 94.

Nyamtema, A. S., Urassa, D. P., Pembe, A. B., Kisanga, F. & van Roosmalen, J. 2010. Factors for change in maternal and perinatal audit systems in Dar es Salaam hospitals, Tanzania. *BMC Pregnancy Childbirth,* 10**,** 29.

Pattinson, R., Kerber, K., Waiswa, P., Day, L. T., Mussell, F., Asiruddin, S. K., Blencowe, H. & Lawn, J. E. 2009. Perinatal mortality audit: counting, accountability, and overcoming challenges in scaling up in low- and middle-income countries. *Int J Gynaecol Obstet,* 107 Suppl 1**,** S113-21, S121-2.

Pattinson, R. C., Say, L., Makin, J. D. & Bastos, M. H. 2005. Critical incident audit and feedback to improve perinatal and maternal mortality and morbidity. *Cochrane Database Syst Rev***,** CD002961.

Pearson, L., deBernis, L. & Shoo, R. 2009. Maternal death review in Africa. *Int J Gynaecol Obstet,* 106**,** 89-94.

Purandare, C., Bhardwaj, A., Malhotra, M., Bhushan, H. & Shah, P. K. 2014. Every death counts: electronic tracking systems for maternal death review in India. *Int J Gynaecol Obstet,* 127 Suppl 1**,** S45-9.

Raven, J., Hofman, J., Adegoke, A. & Van Den Broek, N. 2011. Methodology and tools for quality improvement in maternal and newborn health care. *International Journal of Gynecology and Obstetrics,* 114**,** 4-9.

Rhoda, N. R., Greenfield, D., Muller, M., Prinsloo, R., Pattinson, R. C., Kauchali, S. & Kerber, K. 2014. Experiences with perinatal death reviews in South Africa--the Perinatal Problem Identification Programme: scaling up from programme to province to country. *BJOG,* 121 Suppl 4**,** 160-6.

Richard, F., Ouedraogo, C., Zongo, V., Ouattara, F., Zongo, S., Gruenais, M. E. & De Brouwere, V. 2009. The difficulty of questioning clinical practice: experience of facility-based case reviews in Ouagadougou, Burkina Faso. *BJOG,* 116**,** 38-44.

Scott, H. & Danel, I. 2016. Accountability for improving maternal and newborn health. *Best Practice & Research: Clinical Obstetrics & Gynaecology,* 36**,** 45-56.

Smith, H., Ameh, C., Godia, P., Maua, J., Bartilol, K., Amoth, P., Mathai, M. & van den Broek, N. 2017a. Authors' Response to Editorial: Maternal Death Surveillance and Response: A Tall Order for Effectiveness in Resource-Poor Settings. *Glob Health Sci Pract,* 5**,** 697-698.

Smith, H., Ameh, C., Godia, P., Maua, J., Bartilol, K., Amoth, P., Mathai, M. & van den Broek, N. 2017b. Implementing Maternal Death Surveillance and Response in Kenya: Incremental Progress and Lessons Learned. *Glob Health Sci Pract,* 5**,** 345-354.

Smith, H., Ameh, C., Roos, N., Mathai, M. & Broek, N. V. D. 2017c. Implementing maternal death surveillance and response: a review of lessons from country case studies. *BMC Pregnancy Childbirth,* 17**,** 233.

Tapesana, S., Chirundu, D., Juru, T., Shambira, G., Gombe, N. T. & Tshimanga, M. 2017. Evaluation of the Maternal Death Surveillance and Response System, Sanyati, Zimbabwe 2017. *Texila International Journal of Public Health,* 5.

van Hamersveld, K. T., den Bakker, E., Nyamtema, A. S., van den Akker, T., Mfinanga, E. H., van Elteren, M. & van Roosmalen, J. 2012. Barriers to conducting effective obstetric audit in Ifakara: a qualitative assessment in an under-resourced setting in Tanzania. *Trop Med Int Health,* 17**,** 652-7.

WHO 2011. Summary report on the consultative meeting on strengthening maternal, perinatal and neonatal health surveillance systems, Beirut, Lebanon, 28–30 October 2010. Beirut, Lebanon: World Health Organization Regional Office for the Eastern Mediterranean.

WHO 2013. Summary report on the regional meeting on maternal death surveillance and response, Rabat, Morocco, 7–9 October 2013. Rabat, Morocco: World Health Organization Regional Office for the Eastern Mediterranean.

WHO 2014a. Case Study India: Study on the implementation of maternal death review in five countries in the South-East Asia Region of the World Health Organization. New Dehli, India: World Health Organization for South-East Asia.

WHO 2014b. Case Study Indonesia: Study on the implementation of maternal death review in five countries in the South-East Asia Region of the World Health Organization. New Dehli, India: World Health Organization for South-East Asia.

WHO 2014c. Case Study Nepal: Study on the implementation of maternal death review in five countries in the South-East Asia Region of the World Health Organization. New Dehli, India: World Health Organization for South-East Asia.

WHO 2014d. Case Study Sri Lanka: Study on the implementation of maternal death review in five countries in the South-East Asia Region of the World Health Organization. New Dehli, India: World Health Organization for South-East Asia.

WHO 2014e. Study on the implementation of maternal death review in five countries in the South-East Asia Region of the World Health Organization. New Dehli, India: World Health Organization for South-East Asia.

WHO 2016. Strengthening Country Capacity on Maternal and Perinatal Death Surveillance and Response: Report of a South-East Asia Regional Meeting , 16-18 February 2016. Maldives: World Health Organization Regional Office for South-East Asia.
