# Supplementary material for: Maternal and perinatal death surveillance and response in low- and middle-income countries: a scoping review of implementation factors
Source: Health Policy Plan. 2021 Mar 13;36(6):955–73. doi: 10.1093/heapol/czab011 (PMC8227470; doi:10.1093/heapol/czab011)
Supplement: czab011_Supp [file czab011_supp.zip › Table 1.docx]

**Table 1: Overview of search strategy components**

| Summary of search terms | - ("maternal mortality" OR "perinatal death" OR "maternal death" OR "perinatal mortality" OR "fetal mortality" OR "stillbirth") **AND** (“audit” OR "surveillance and response"). |
| --- | --- |
| Concept component | - All forms of maternal and perinatal death review including obstetric audit, MPDSR, MDSR, MDR - Limited to studies or perspectives that identify factors that influence the implementation process - Excluded near miss audits as well as other forms of maternal and perinatal death surveillance e.g. confidential inquiries, social autopsy, and verbal autopsy.* |
| Context component | - Limited to low and middle income countries listed by the World Bank in 2018. |

*For definitions of these terms, please see Lewis (2014).
