# Supplementary material for: Maternal and perinatal death surveillance and response in low- and middle-income countries: a scoping review of implementation factors
Source: Health Policy Plan. 2021 Mar 13;36(6):955–73. doi: 10.1093/heapol/czab011 (PMC8227470; doi:10.1093/heapol/czab011)
Supplement: czab011_Supp [file czab011_supp.zip › Table 2.docx]

**Table 2: Results of search by source**

| **Database** | **Number of articles** |
| --- | --- |
| *PubMed* | *434* |
| *CINAHL* | *264* |
| *SCOPUS* | *658* |
| *Web of Science* | *432* |
| *JSTOR* | *214* |
| *LILACS* | *7* |
| **Database search** | **2009** |
| *MDSR Network* | *16* |
| *WHO IRIS* | *50* |
| *Google* | *29* |
| **Online search** | **95** |
| *Consultation* | *8* |
| *Reference list* | *10* |
| **Additional search** | **18** |
| Total identified | 2122 |
| Duplicates | 1095 |
| **TOTAL SCREENED** | **1027** |
