# Supplementary material for: Maternal and perinatal death surveillance and response in low- and middle-income countries: a scoping review of implementation factors
Source: Health Policy Plan. 2021 Mar 13;36(6):955–73. doi: 10.1093/heapol/czab011 (PMC8227470; doi:10.1093/heapol/czab011)
Supplement: czab011_Supp [file czab011_supp.zip › Table 3.docx]

**Table 3: Synthesis of results by theoretical conceptual framework**

| **Domain** | **Construct** | **# Records described*** | **Summary of results** |
| --- | --- | --- | --- |
| Domain 1: Intervention/ MPDSR | Components and execution: steps of the audit cycle described and reported on by level (a descriptive analysis) | 34 | - ¾ of papers described the audit cycle (43/58); but only 17 studies described all steps of the audit cycle; half of the papers reported data collection, the review process and implementation of the recommendations (52%, 53%, 52%, respectively); notification and evaluation received the least amount of attention (39% each)   Literature reflects evolution of intervention over time i.e. clinical audits to maternal and/or perinatal death reviews to MDSR to MPDSR. |
|  | Cost relating to the audit process including collecting data, meeting related costs such as transport, specific training, running secretariat, time | 34 | - Described as funds for training, transport and dissemination of results; human resources such as staff workload, staff shortages, staff turnover and staff skills - Few studies reported on budgets and actual costs; where studied, no standard costing approach used - Barrier identified as limited financial resources (without quantification) |
|  | Framing - Intervention source: ownership of implementation guideline and stakeholder perceptions on whether the intervention is externally or internally developed | 41 | - Described as government initiated, externally driven by partners, or embedded in the system - One study reported as “top down” approach being problematic - Reported that countries adapt from the global WHO guidelines applying and adapting the recommendations to their context, but not explored - Stakeholder perceptions of legitimacy not explored |
|  | Framing - Evidence strength & quality: Evidence supporting the belief that the intervention will have desired outcomes (reduced mortality; changes undertaken to improve quality of care / "response") | 31 | - Described from the perspective of stakeholders that MPDSR resolves critical gaps in quality of care but little documentation of actual changes made |
|  | Framing - Relative advantage: Perception of the advantage of implementing the intervention versus an alternative solution | 0 | - Not described |
|  | Trialability: Ability to test/ pilot the intervention on a small scale, learn and revise if warranted | 22 | - Described a phased approach, but little documentation of learning from the phasing - Identified 9 pilot studies, most conducted at facility level (only one was at sub-national level); no reporting of modifications or expansion after these pilots - Enablers included local leadership and initial external support - Barriers included sustained implementation beyond projects |
|  | Adaptability: Degree to which an intervention can be tailored to meet the needs of an organization (core vs. peripheral elements) | 15 | - Described as MPDSR processes adapting and changing over time but no evidence to show which factors were essential vs peripheral to change - Variations in implementation observed across facilities in same country, sub-national levels and countries with different drivers of the process or frequency of review meetings |
| Domain 2: Individual | Technical skills & Knowledge: Individual staff knowledge and competencies including skills for data collection and data use | 31 | - Described as important to complete MPDSR process, with most studies making broad based statements about skills. - Barriers included record keeping, data entry, identification and reporting of deaths, use of data for routine analysis, and familiarity with audit process - Level of knowledge assessed in four studies |
|  | Self-efficacy: Individual belief in their own capabilities to execute courses of action to achieve implementation goals. | 8 | - Described with mixed results on individual confidence to implement MPDSR (e.g. confident or not). - Enablers included supportive supervision, appropriate tools and oversight from management or health specialists. |
|  | Individual motivation: A broad construct related to factors that motivate individuals to implement both extrinsic and intrinsic | 23 | - Described extrinsic motivation as measures to improve quality of care, adhering to expectation from sub-national teams, gaining skills or knowledge and incentives - Described intrinsic motivation as consciousness for self-improvement linked to the underlying value of life - De-motivating factors included lack of resources to support M/PDSR processes, lack of implementation of MPDSR related recommendations, hierarchical nature of meetings, the process perceived as time consuming and arduous |
|  | Individual identification with intervention: A broad construct related to how individuals perceive the intervention, and their relationship and degree of commitment to the sustained use of the intervention. | 18 | - Described as important but not explored adequately - Enablers included link between individual commitment to jobs and general quality improvement as well as individuals seeing the benefit of process improving quality over time - Barrier included “passing the buck” to other staff |
|  | Individual orientation to collaboration: Personal traits such as tolerance of ambiguity, team player, flexibility, problem solving, critical thinking | 0 | - Not described |
| Domain 3: Inner setting | Readiness for Implementation: Tangible and immediate indicators of organizational commitment to its decision to implement an intervention | 48 | - Enablers described as formation and or existence of MPDSR committees, a designated focal person, regularly scheduled meetings, available tools and appropriate forms for MPDSR, and “audit charters”, training - Barriers described as shortage and capacity of health workers and disengaged leadership and inadequate management capacity |
|  | Team composition and characteristics including who comprises the team e.g. size, interdisciplinary nature, membership regulation | 36 | - Described as multidisciplinary, though some studies noted low participation of nurses. - Barriers identified included high staff turnover, competing priorities, lack of interest by staff, and hierarchical nature of meetings. |
|  | Organizational incentives & rewards (or disincentives/sanctions) such as goal-sharing awards, performance reviews/promotions, training, tea or the consequences | 11 | - Enabler described as refreshments, extra training, financial motivation (per diems), and transportation. - Described removal of funding that financed incentives as a demotivating factor - Not adequately investigated for impact |
|  | Team relationship: nature and quality of communication within audit team (including hierarchies, mentorship, teamwork, and management) | 19 | - Described as both positively and negatively affected by the nature of communication, collaboration, management and networking within and across teams and among stakeholders involved in the implementation process - Enablers included continuous engagement, a teamwork approach, support from hospital management, invested deliberate efforts and strategies such as mentorship, as well as upholding certain norms and values to nurture a conducive atmosphere - Teamwork approach involving consensus building, inclusiveness, delegation of responsibility and continuity of MPDSR as important factors - Hierarchies within teams can both positively and negatively influence relationships. |
|  | Implementation culture and climate: explanation of environment including organizational culture, learning climate, if there are things mentioned that are tensions/triggers for change | 47 | - Enabler described as an implementation culture of accountability, learning and improvement; effective strategies included the mandatory attendance of audit meetings as well as codes of conduct or “audit charters” - Barriers described as a blame culture and punitive measures against frontline health providers - Blame culture explored at individual level, as well as between levels of the health system and between units with mixed observations around blame-free and blame culture. |
|  | Engaged leaders: Individuals who have formal or informal influence on the attitudes and beliefs of their colleagues with respect to implementing the intervention or on the implementation process overall, e.g. “champions” or “agents of change” | 21 | - Described as a critical factor for successful implementation - Strong leaders are described as highly motivated individuals who can facilitate the process well - Individual traits and motivations not investigated |
| Domain 4: Outer setting | Policy and planning: MPDSR policy and guidelines, Death notification requirements, Legal mandate, litigation/legal protection | 41 | - Described as the type of policy or guideline in place ie integrated, standalone and M/PDSR related guidelines; few studies reported on the presence of a legal framework or protocol around death notification - Descried as implementation factor the uptake of national policies and technical guidance and the presence of legal framework or protocol around death notification, but not explored |
|  | Resource support: funding or resource support for MPDSR (e.g. sponsors, budgets) | 29 | - Described as funding source e.g. government budget line, government commitment, development partner support - Barrier to implementation included lack of a budget - Budgets linked to spending explored in some studies with mixed findings |
|  | External actors: The role of external actors on the process (e.g. Local party, Union affiliations, Professional associations, Community organisations) as well as community or CHW engagement and participation in MPDSR | 31 | - Described as the roles of key external actors, including national government, international development partners, professional associations and civil society, having influence at a subnational or facility level from strong national or subnational actors and influence at a national level from externally partners e.g. WHO, UNFPA and donor agencies - Supportive supervision reported as an implementation factor - Barrier identified as absence of external actor engagement - The role of development partners (UN agencies and NGOs) and professional associations at all levels described and explored, e.g. developing guidelines, training facility staff and mobilizing resources as well as pressuring governments (mostly at national level) to implement - Engagements with private sector, communities, civil society and local authorities described but not explored adequately |
|  | Political prioritization: national mobilization and awareness of issue | 10 | - Described as pressure to implement MPDSR but not explored adequately |
|  | Pressure: to implement from actors and other implementers | 17 | - Described as peer pressure for system wide uptake especially from sub-national structures to facility level - Barrier identified as lack of national and sub-national pressure to implement - Perceptions around pressure to implement explored by only one study |
|  | Linkages and networks between levels: Level of connectedness and networks with other health system levels, organizations and therefore openness or resistance to change | 34 | - Described as the level of connectedness and networks between health system levels, different sites and different role players influences implementation - Enablers identified as existing strong communication channels between and within levels; well-defined pathways around the flow of data and information relating to MPDSR; and well implemented supportive supervision - Barrier identified as lack of an adequate and coherent guidance or framework to channel communication of MPDSR recommendations across levels |

* See Supplementary 3, Table S3.4 for references to records by construct
