# Supplementary material for: Maternal and perinatal death surveillance and response in low- and middle-income countries: a scoping review of implementation factors
Source: Health Policy Plan. 2021 Mar 13;36(6):955–73. doi: 10.1093/heapol/czab011 (PMC8227470; doi:10.1093/heapol/czab011)
Supplement: czab011_Supp [file czab011_supp.zip › Box 1.docx]

Box 1: Overview of the conceptual implementation framework for MPDSR

The theoretical conceptual framework developed for this review is adapted from the Consolidated Framework for Implementation Research (Damschroder et al. 2009), and well described in the protocol paper (Kinney et al. 2019).

The visual of the framework (Figure 1) shows that MPDSR functions at multiple levels of the health system – national, subnational, facility (and for some countries community level components are included in the process). The communication system and inter-connectedness between the different levels is an important component of M/PDSR since the process is a reporting mechanism moving continuously from bottom up – facility to national – and also from top down – national to facility. It also shows that there is MDPSR in theory, e.g. how it should work based on guidelines, and that there is MPDSR in practice, e.g. how it actually works.

The framework includes three different lenses through which to understand and measure health system drivers of women’s and children’s health (George and al. 2019 ). A service delivery lens includes the tangible inputs needed for MPDSR implementation; a societal lens includes constructs that focus on social understanding and relationships; and a systems lens includes constructs that emphasis change dynamics, which includes adaptive learning to contexts in ways that are not always anticipated. The factors within each domain are categories by lens, which are denoted by grey-shading in the figure.

The framework considers four domains with 24 constructs in total:

- Intervention: The first domain is MPDSR or any related form of maternal and/or perinatal death review or audit. Factors within this domain for MPDSR include the components of the audit cycle and costs relating to the audit process from a service delivery lens, framing of the intervention source, evidence strength and quality and relative advantage from a societal lens, and the perceived ability to test and adapt it from a systems lens.
- Individual: The next domain considers the characteristics of the individuals involved in implementation. From a service delivery lens, factors include their technical skills and knowledge; from a societal lens, factors include their self-efficacy, motivations and identification with the intervention; and from a systems lens, factors include their ability to move from orientation to collaboration.
- Inner setting: The third domain considers factors internal to the organization. From a service delivery lens, this includes the readiness to implement, team composition and characteristics, and incentives to implement; from a societal lens, this includes team relationships; and from a systems lens, this includes the organizational culture and implementation climate, and engagement of leaders (often called “champions”).
- Outer setting: The final domain considers factors external to the organization that influence implementation of MPDSR. These factors include policy and planning and resource support or funding for MPDSR from a service delivery lens; the role of external actors (such as professional association) and political prioritization from a societal lens; and from the pressures to implement and the linkages and networks between levels from a systems lens.

Supplementary 2 further describes the framework and includes an overview of how the framework was adapted and evolved during the data extraction and analysis process of the scoping review.
