# Supplementary figures and images for: Maternal and perinatal death surveillance and response in low- and middle-income countries: a scoping review of implementation factors
Source: Health Policy Plan. 2021 Mar 13;36(6):955–73. doi: 10.1093/heapol/czab011 (PMC8227470; doi:10.1093/heapol/czab011)

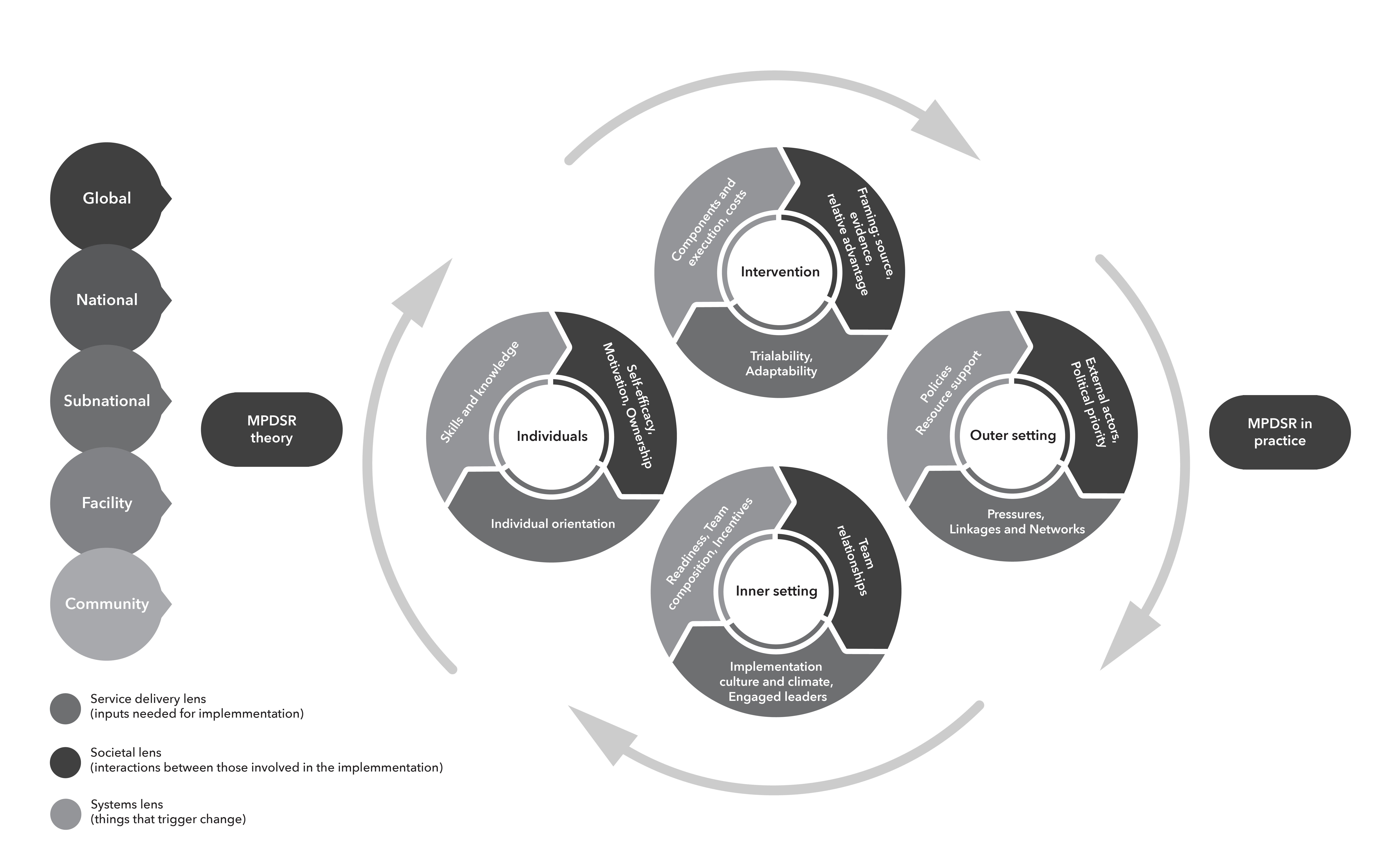

Supplement: czab011_Supp [file czab011_supp.zip › High res figure framework.jpg]

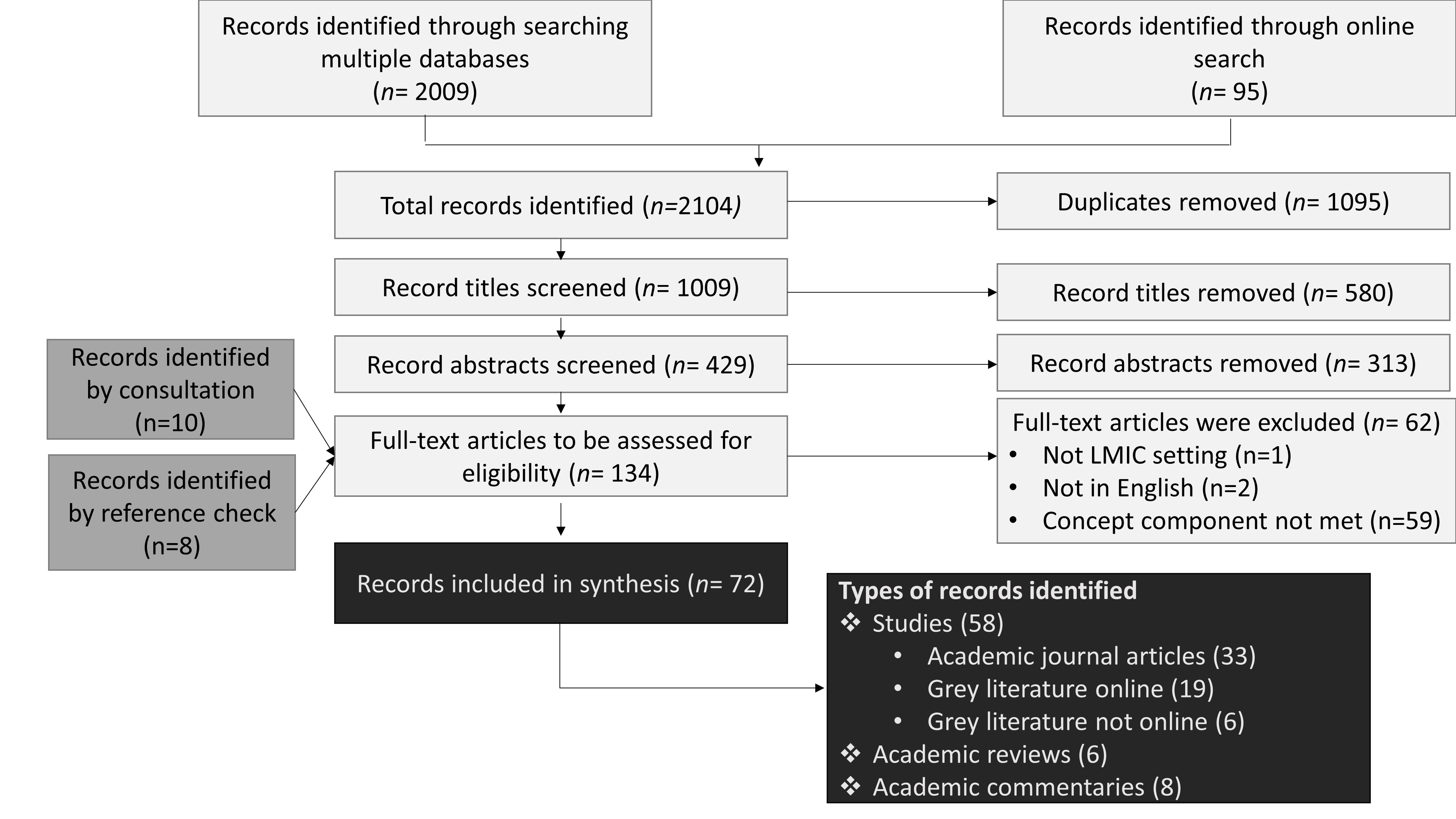

Supplement: czab011_Supp [file czab011_supp.zip › Figure 2.jpg]
